# Pulse Dipolar Electron Paramagnetic Resonance Spectroscopy Reveals Buffer-Modulated Cooperativity of Metal-Templated Protein Dimerization

**DOI:** 10.1021/acs.jpclett.2c01719

**Published:** 2022-08-17

**Authors:** Maria Oranges, Joshua L. Wort, Miki Fukushima, Edoardo Fusco, Katrin Ackermann, Bela E. Bode

**Affiliations:** EaStCHEM School of Chemistry and Biomedical Sciences Research Complex, Centre of Magnetic Resonance, University of St Andrews, North Haugh, St. Andrews KY16 9ST, U.K.

## Abstract

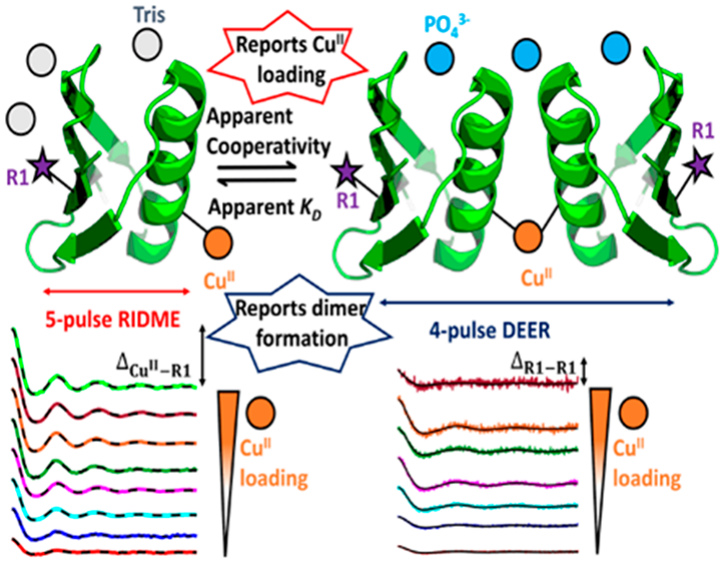

Self-assembly of protein monomers directed by metal ion coordination constitutes a promising strategy for designing supramolecular architectures complicated by the noncovalent interaction between monomers. Herein, two pulse dipolar electron paramagnetic resonance spectroscopy (PDS) techniques, pulse electron–electron double resonance and relaxation-induced dipolar modulation enhancement, were simultaneously employed to study the Cu^II^-templated dimerization behavior of a model protein (*Streptococcus* sp. group G, protein G B1 domain) in both phosphate and Tris-HCl buffers. A cooperative binding model could simultaneously fit all data and demonstrate that the cooperativity of protein dimerization across α-helical double-histidine motifs in the presence of Cu^II^ is strongly modulated by the buffer, representing a platform for highly tunable buffer-switchable templated dimerization. Hence, PDS enriches the family of techniques for monitoring binding processes, supporting the development of novel strategies for bioengineering structures and stable architectures assembled by an initial metal-templated dimerization.

Self-assembly of protein monomeric units is of great interest in supramolecular complex design,^1−3^ but because of the noncovalency of quaternary structural interactions, mimicking their functionality for the synthesis of new protein complexes is demanding.^3^ Metal coordination is used as a driving force for the assembly of protein monomers, leading to a degree of multimerization that depends on the metal ion coordination geometry.^4−6^ Different strategies employed the engineering of chelating motifs of natural^7^ and unnatural^8^ amino acid residues, the incorporation of non-natural ligands onto protein surfaces,^9,10^ and the construction of hybrid coordination motifs,^10^ resulting in metal-induced increased stability of the multimer due to higher metal binding affinity. Protein–protein interfaces nucleated by metal coordination have led to the formation of two- and three-dimensional crystalline protein lattices^11,12^ and novel functional materials.^13^ Of particular interest is the formation of metal-bridged dimers because they are considered to be the precursor of more complex assemblies.^3^ Several techniques such as X-ray crystallography, nuclear magnetic resonance (NMR), and sedimentation velocity have extensively characterized these binding processes.^5,7,14^ Pulse dipolar electron paramagnetic resonance spectroscopy (PDS) has been employed to characterize metal motifs that induce polymerization^15^ and the conformational flexibility of supramolecular polymers.^16^ Moreover, PDS has recently emerged as an excellent complementary tool for studying metal ion binding equilibria with submicromolar sensitivity.^17−21^

The four-pulse DEER^22−24^ (double electron–electron resonance) and the five-pulse RIDME^25,26^ (relaxation-induced dipolar modulation enhancement) experiments (for pulse sequences, see section 1.3 of the Supporting Information) allow detection of the weak dipolar interaction between paramagnetic centers, which is characterized by modulation with the dipolar frequency (ω_AB_) that encodes the interspin distance, *r*_AB_. The modulation depth (Δ) of these traces (i.e., the amplitude between the signal intensity at time zero and the time when the signal is entirely damped in the limit of negligible intermolecular decay) informs the number of coupled spins.^27^ In previous studies, pulse electron–electron double resonance (PELDOR, mainly in the form of the four-pulse DEER experiment) and RIDME were employed individually to monitor the metal-templated dimerization of a nitroxide-labeled terpyridine-based ligand model system using different divalent metal ions as templates^19−21^ and proved the feasibility of monitoring binding events at cryogenic temperatures, with the modulation depth informing on the degree of binding at a given metal:ligand ratio.^20^

Here, PELDOR and RIDME were employed complementarily to study the metal-templated dimerization of a protein model system, the B1 immunoglobulin-binding domain of protein G of *Streptococcus* sp. group G (GB1). Double-histidine (dHis) motifs are incorporated as artificial metal-binding sites,^28^ making this system particularly suitable for this study. Previous works have used this system as a biological model for PDS studies,^18,28−30^ improving the precision and accuracy of distance measurements due to the increased rigidity of Cu^II^-chelate spin-labels through bipedal attachment.^18,28^ The I6R1/K28H/Q32H and I6H/N8H/K28R1 constructs (Figure 1) were selected for this work as they were used in previous studies;^18,31,32^ however, their propensity for metal-templated dimerization was unexplored. Screening revealed that only the I6R1/K28H/Q32H construct gave an appreciable PELDOR modulation depth when bound to Cu^II^ and Zn^II^ (see section 2.1 of the Supporting Information and dataset^45^), suggesting that metal-templated dimerization may occur only across the α-helix motif and not across the β-sheet motif. This disparity is potentially explained by steric effects, as well as reduced apparent binding affinity of Cu^II^ at the β-sheet motif. Additionally, the modulation depth was maximized for the Cu^II^ series, which is not entirely surprising because it is consistent with the finding of metal-induced stabilization of the α-helix motif^33,34^ via histidine residues and higher apparent affinity for Cu^II^ than for Zn^II^ and other metal ions, per the Irving–Williams series.^34^

**Figure 1:**
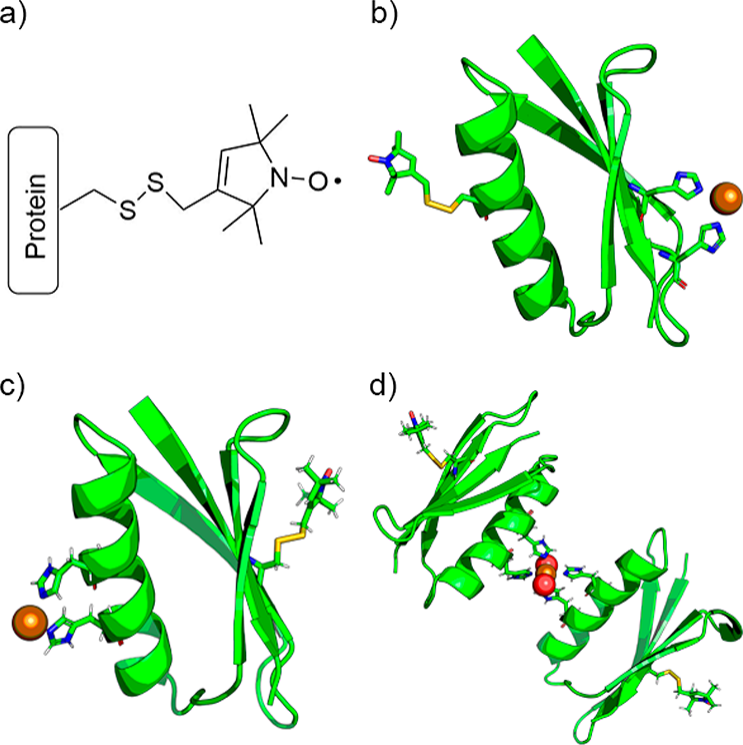
Representations of (a) the nitroxide spin-label MTSL conjugated to a cysteine residue, resulting in the R1 side chain; of the monomers for constructs (b) I6H/N8H/K28R1 and (c) I6R1/K28H/Q32H with Cu^II^ ions shown as bronze spheres and R1 side chains and dHis motifs shown as sticks; and of (d) the putative metal-templated dimeric structure. The axial solvent ligands are shown as red spheres.

The PELDOR method measures the intermolecular nitroxide–nitroxide (R1–R1) distances within the metal-templated dimer and therefore provides direct information about dimer formation. The modulation depth, Δ_R1–R1_, depends on the dipolar interaction between nitroxide moieties of each GB1 monomer. The RIDME experiment measures the intramolecular metal–nitroxide distances (M–R1) and is a reporter of all metal-bound species. The modulation depth, Δ_M–R1_ provides information about the formation of dimers coordinated around the metal template and fractional saturation of the metal-binding site, the dHis motif. To characterize these binding equilibria, a cooperative binding model (see section 1.7 of the Supporting Information) was used to fit simultaneously Δ_R1–R1_ and Δ_M–R1_ values.^35^ This allows simultaneous characterization of an apparent dissociation constant (*K*_D_) for initial metal binding and an apparent cooperativity factor (α) for the metal-templated dimerization event. Determination of the true dissociation constant and cooperativity factor is obscured because the precise Cu^II^ concentration available for binding cannot be quantified, because of the competition with the buffer and unspecific Cu^II^ binding at the protein surface. Such effects are not treated by the binding model, and instead, apparent thermodynamic parameters are extracted.

First, measurements were performed as a nine-point pseudotitration series (where each data point was a discrete sample) for Cu^II^, in the presence of phosphate buffer [150 mM NaCl, 42.4 mM Na_2_HPO_4_, and 7.6 mM KH_2_PO_4_ (pH 7.4)], having been used extensively for previous EPR methodological work involving GB1 and Cu^II^-NTA.^30−32^Figure 2 shows the background-corrected traces for RIDME and PELDOR measurements of the phosphate buffer series. The corresponding validated distance distributions yielded significant peaks (i.e., above the noise floor) at ∼2.5 and ∼5.0 nm, respectively (see section 2.3 of the Supporting Information).

**Figure 2:**
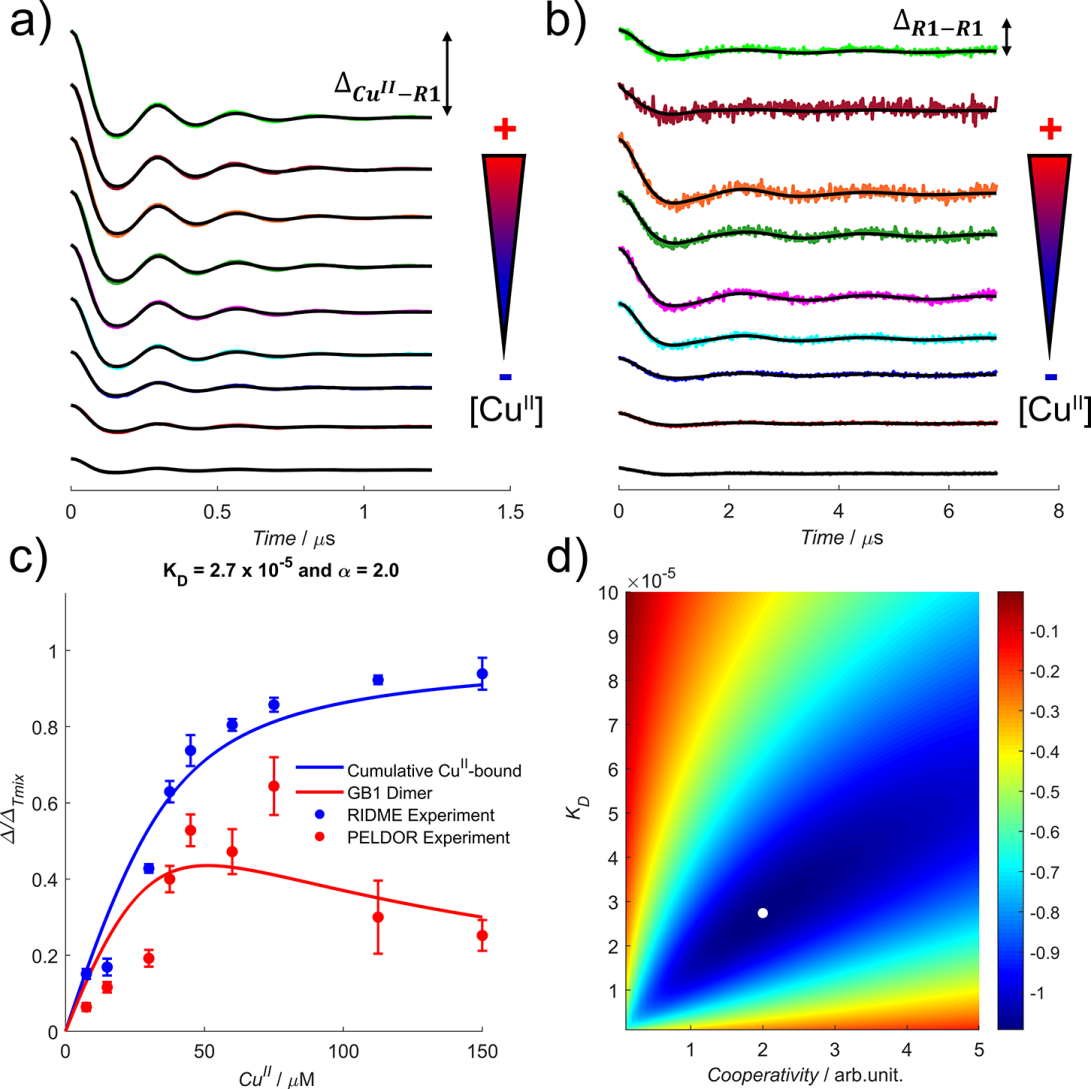
In phosphate buffer, (a) deconvoluted RIDME and (b) PELDOR background-corrected traces with the modulation depth Δ indicated, (c) bivariate fitted modulation depth profiles as a function of Cu^II^ concentration for RIDME (blue scatter) and PELDOR (red scatter) data, and (d) the corresponding error contour, for the phosphate buffer series. Data in panels a and b are offset vertically to aid visualization, and the direction of increasing Cu^II^ concentration is indicated by the arrows. The 95% confidence intervals of RIDME and PELDOR modulation depths in panel c are shown as the blue and red error bars, respectively. The fitted apparent α and *K*_D_ values in panel d are indicated by the white dot.

In Figure 2, the Δ_M–R1_ behavior was consistent with a reduced apparent binding affinity of Cu^II^ for the dHis motif in phosphate buffer, with <80% occupancy at a metal:protein ratio of 1:1. Indeed, Δ_M–R1_ was systematically lower for the phosphate buffer series than for the Tris-HCl buffer series (Supporting Information and *vide infra* for details). Interestingly, the Δ_R1–R1_ behavior suggested that in phosphate buffer, metal-templated dimer formation was optimized at a metal:protein ratio of 1:1 and persists even above a stoichiometric Cu^II^ concentration. Replicate measurements of the phosphate buffer series (see the Supporting Information) reproduced this observation, and the fitted parameters (α = 2, and *K*_D_ = 2.7 × 10^–5^) further supported the positive cooperativity of dimerization. This affinity agrees with reported literature values for the binding of Cu^II^ to histidine residues on a solvent-exposed α-helix.^36,37^

Aware of the possible precipitation of Cu^II^ in the presence of phosphate salts,^38^ we chose a second buffer by preparing a concentration series of CuCl_2_ in Good’s buffers and examining which retained free Cu^II^ in solution under alkali conditions via CW-EPR measurements (see section 2.4 of the Supporting Information). CuCl_2_ precipitated in PBS and MOPS buffers, but Tris-HCl buffer [150 mM NaCl and 20 mM Tris-HCl (pH 7.4)] retained Cu^II^ in solution. For this reason, Tris-HCl was adopted for this study. Interestingly, CW-EPR data showed the phosphate buffer retained ∼15–60% of Cu^II^ in solution when in the presence of 2 equiv of imidazole or 0.5 equiv of K28H/Q32H GB1 (see the Supporting Information). Leaving a solution of CuCl_2_ in phosphate buffer to equilibrate led to negligible available Cu^II^ (i.e., subsequent incubation with protein yielded very poor PELDOR modulation depths). From these observations, we hypothesized that the changing availability of metal ions in solution would modify the binding equilibria reflected by the modulation depths of our measurements.

Measurements were then performed as an eight-point pseudotitration series for Cu^II^ in the presence of Tris-HCl buffer. Figure 3 shows the background-corrected traces for RIDME and PELDOR measurements of the Tris-HCl buffer series.

**Figure 3:**
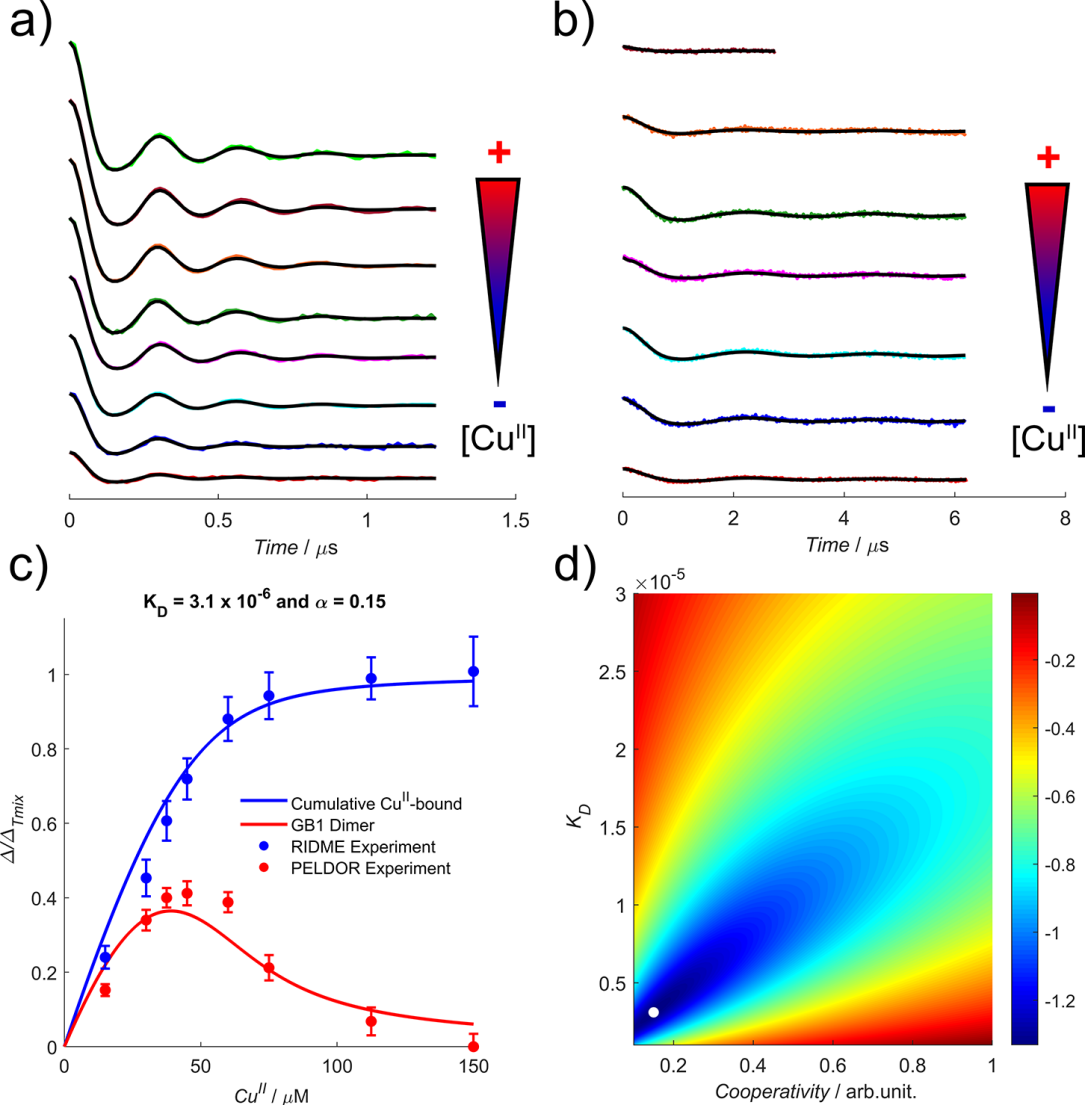
In Tris-HCl buffer, (a) deconvoluted RIDME and (b) PELDOR background-corrected traces, (c) bivariate fitted modulation depth profiles as a function of Cu^II^ concentration for RIDME (blue scatter) and PELDOR (red scatter) data, and (d) the corresponding error contour, for the Tris-HCl buffer series. Data in panels a and b are offset vertically to aid visualization, and the direction of increasing Cu^II^ concentration is indicated by the arrows. The 95% confidence intervals of RIDME and PELDOR modulation depths in panel c are shown as the blue and red error bars, respectively. The fitted apparent α and *K*_D_ values in panel d are indicated by the white dot.

In Figure 3, the Δ_M–R1_ behavior indicated a high binding affinity of Cu^II^ for the dHis motif, with >90% occupancy at a metal:protein ratio of 1:1. The Δ_R1–R1_ behavior suggested that metal-templated dimer formation was optimized at a metal:protein ratio of 4:5, with a Δ_R1–R1_ marginally higher than that at a metal:protein ratio of 1:2 (see the Supporting Information), but was abolished entirely above stoichiometric ratios of Cu^II^, where either (i) significant cutting of data was required for processing or (ii) the detected echo was free of dipolar modulation. Replicates of metal:protein ratios of 3:5 and 4:5 (see the Supporting Information) showed optimized dimer formation at a metal:protein ratio of 3:5, which is consistent with a negative cooperativity mode of templated dimerization (i.e., the initial dHis motif binding event outcompetes the formation of the dimer construct), and the global fitting of the *K*_D_ and α parameters supports it further. Exploratory simulations validated the robustness of the cooperative binding model (see section 2.5 of the Supporting Information) and indicated a strongly negative cooperativity parameter (α = 0.15) and a *K*_D_ of 3.1 × 10^–6^.

Despite the imperfect agreement between the fitted *K*_D_ and α parameters and the phosphate series experimental data manifest by Cu^II^ precipitating from solution, bivariate fitting of repeat measurements (see the Supporting Information) indicated that the positive cooperativity and reduced initial binding affinity compared to those of the Tris-HCl buffer series were reproducible. Additionally, scaling the experimental Cu^II^ concentration by 0.65 for the phosphate and 0.85 for the Tris-HCl buffer series yielded global root-mean-square deviation minima upon reprocessing (see the Supporting Information). This observation is consistent with the CW-EPR data (see the Supporting Information) for the phosphate buffer series showing that only ∼60% of Cu^II^ is retained in the solution, while for the Tris-HCl buffer series, scaling by a factor of 0.85 corresponds to a shift in optimized dimer formation from a metal:protein ratio of 3:5 to 1:2, as expected for negative cooperativity.

These observations can be rationalized by the strong negative cooperativity for the Tris-HCl buffer (the metal-templated dimer formation would be disfavored, i.e., the initial metal binding event outcompetes the formation of the templated dimer) by considering Tris-HCl interacts strongly with Cu^II^,^39^ retaining it in solution. This maximizes the effective Cu^II^ concentration that can bind dHis motifs, and because for templated dimer formation, one monomer must have an unoccupied dHis motif, Tris-HCl buffer disfavors this. On the contrary, precipitation of Cu^II^ as Cu_3_(PO_4_)_2_ reduces the effective Cu^II^ concentration in solution.^38^ The lower availability of Cu^II^ reduces the apparent affinity of dHis motifs; fewer dHis motifs are occupied, and metal-templated dimer formation is favored.

It is important to note that additional low-affinity Cu^II^-binding sites on the protein surface, precipitation in alkaline pH, and complexation by buffer components are all potentially limiting the available Cu^II^ for ligation by one or two dHis motifs. In this context, the study of, e.g., morpholine-based buffers effective under mildly acidic conditions (such as MES) may be interesting for maximizing the availability of Cu^II^.

Positive cooperativity in metal-templated dimerization is considered to be rare,^40^ while negative cooperativity characterizes many systems.^41−44^ The potential utility of the α-helical dHis motif for protein–protein interface nucleation by metal binding is already well-known;^7,33,34^ however, the observation that cooperativity of templated dimerization is modulated by the buffer provides an additional handle for manipulation of the binding equilibrium. We serendipitously observed that in the presence of phosphate buffer, Cu^II^-templated dimerization demonstrated apparent positive cooperativity, while in Tris-HCl buffer, this templated dimerization displayed strongly negative cooperativity behavior.

Additionally, to the best of our knowledge, this is the first pulse dipolar EPR methodology to extract apparent cooperativity and *K*_D_ parameters by global fitting of nitroxide–nitroxide PELDOR and Cu^II^–nitroxide RIDME modulation depths. The results also showcase the robustness and accuracy of PDS in monitoring equilibrium processes and in detecting variations of cooperativity mode while changing buffer conditions (J. L. Wort et al., manuscript in preparation). Finally, this methodology can be easily complemented by other PDS measurements (for instance, by Cu^II^–Cu^II^ RIDME modulation depths or comparison of validated distance distributions) to study the cooperativity behavior of more complex systems with nonspecific binding contributions (i.e., of Cu^II^ binding away from the dHis motifs), which are notoriously difficult to disentangle without overfitting by other techniques such as isothermal titration calorimetry.

## Supporting Information Available

Experimental Procedures, including construct design, expression, and purification, EPR sample preparation, pulse EPR measurement parameters, continuous-wave EPR measurement parameters, pulse dipolar EPR and data processing and validations, continuous-wave EPR data processing, analytical cooperative binding model, and in silico modeling of the metal-templated dimer; Results and Discussion, including screening of dimer formation for different constructs and templates, inversion recovery measurements, validated PELDOR and RIDME measurements, continuous-wave EPR measurements, estimating *K*_D_ and cooperativity (α) parameters, and in silico modeling of the metal-templated dimer; and References (PDF)
Transparent Peer Review report available (PDF)

## Notes

The research data supporting this publication can be accessed at 10.17630/bacc1277-fbfb-440f-bbc2-303aa11cd7e5.
