# Supplementary material for: Pulse Dipolar Electron Paramagnetic Resonance Spectroscopy Reveals Buffer-Modulated Cooperativity of Metal-Templated Protein Dimerization
Source: J Phys Chem Lett. 2022 Aug 17;13(33):7847–52. doi: 10.1021/acs.jpclett.2c01719 (PMC9421889; doi:10.1021/acs.jpclett.2c01719)
Supplement: Supplementary file 1 — jz2c01719_si_001.pdf [file jz2c01719_si_001.pdf]

Pulse Dipolar Electron Paramagnetic Resonance Spectroscopy Reveals Buffer Modulated Cooperativity of Metal Templated Protein Dimerization.

Supplementary Material

*Maria Oranges,[†] Joshua L. Wort, Miki Fukushima, Edoardo Fusco, Katrin Ackermann, and Bela E. Bode**

EaStCHEM School of Chemistry and Biomedical Sciences Research Complex, Centre of Magnetic Resonance, University of St. Andrews, North Haugh, St. Andrews, KY16 9ST, U.K.

[†]Current address: Department of Chemical and Biological Physics, Weizmann Institute of Science, Rehovot, 76100 Israel

Corresponding Author

*beb2@st-andrews.ac.uk

Table of Contents

I **Experimental Procedures**……………………………………………………………………………………………S3

1.1 Construct Design, Expression and Purification……………………………………………………………S3

1.2 EPR Sample Preparation…………………………………………………………………………………………S3

1.3 Pulse EPR Measurement Parameters……………………………………………………………………………S4

1.4 Continuous-Wave EPR Measurement Parameters…………………………………………………………S7

1.5 Pulse Dipolar EPR Data Processing and Validations……………………………………………………S7

1.6 Continuous-Wave EPR Data Processing……………………………………………………………………..S8

1.7 Analytical Cooperative Binding Model………………………………………………………………………S9

1.8 In Silico Modelling of Metal Templated Dimer………………………………………………………S12

II **Results and Discussion**……………………………………………………………………………………………S13

2.1 Screening of Dimer Formation for Different Constructs and Templates…………………………S13

2.2 Inversion Recovery Measurements………………………………………………………………………….S15

2.3 Validated PELDOR and RIDME Measurements……………………………………………………………S19

2.3.1 Tris Buffer Series…………………………………………………………………………………………………S19

2.3.2 Phosphate Buffer Series…………………………………………………………………………………………S30

2.4 Continuous-Wave EPR Measurements………………………………………………………………………..S45

2.5 Estimating K_D and Cooperativity (α) Parameters……………………………………………………..S50

2.5.1 Exploratory Simulations………………………………………………………………………………………S50

2.5.2 Reproducibility of Phosphate Buffer Series…………………………………………………………..S53

2.5.3 Scaled Fits………S54

2.6 In Silico Modelling of Metal Templated Dimer………………………………………………………S60

2.7 Author contributions……………………………………………………………………………………………….S61

III **References**………S62

I Experimental Procedures:

1.1 Construct Design, Expression, and Purification:

Constructs of *Streptococcus sp.* group G, protein G, B1 domain (GB1) were designed, expressed, purified, and labelled with MTSL nitroxide spin label (labelling efficiency ≥ 95%), as previously described.[1,2] For completeness the protein sequences for the I6H/N8H/K28C and I6C/K28H/Q32H constructs are given below in figures S1 and S2, respectively.

I6H/N8H/K28C GB1 protein sequence:

6 8 28

MQYKLHLHGKTLKGETTTEAVDAATAECVFKQYANDNGVDGEWTTDDATKTFTVTE

Figure S1. Full amino acid sequence of the I6H/N8H/K28C GB1 construct used in this work, with each histidine residue of the double-histidine motif shown in red, and the cysteine residue shown in blue. Residue numbers are indicated above the sequence.

I6C/K28H/Q32H GB1 protein sequence:

6 28 32

MQYKLCLNGKTLKGETTTEAVDAATAEHVFKHYANDNGVDGEWTTDDATKTFTVTE

Figure S2. Full amino acid sequence of the I6C/K28H/Q32H GB1 construct used in this work, with each histidine residue of the double-histidine motif shown in red, and the cysteine residue shown in blue. Residue numbers are indicated above the sequence.

1.2 EPR Sample Preparation:

Unless otherwise stated, all material was exchanged into deuterated buffer A (150 mM NaCl, 42.4 mM Na_2HPO_4 and 7.6 mM KH_2PO_4, pH 7.4) or buffer B (150 mM NaCl, 20 mM Tris-HCl, pH 7.4), respectively, by first freeze-drying and redissolving into D_2O. Cu^{II} and Zn^{II} labelling of double-histidine motifs was performed by using $CuCl_2$ and $ZnCl_2$ in D_2O and diluting to nominal stock concentrations of either 0.5 or 3.0 mM, depending on sample. $CuCl_2$ and $ZnCl_2$ were added directly to the protein before addition of buffer. For all relaxation induced dipolar modulation enhancement (RIDME) and pulse electron-electron double resonance (PELDOR) samples, a total volume of 70 µL was used, with a fixed GB1 protein monomer concentration of 75 µM. As cryoprotectant 50% (v/v) ethylene glycol-d_6 (Deutero) was used, and samples were transferred to 3 mm OD quartz tubes and flash frozen in liquid nitrogen until measured. Continuous-wave (CW)

EPR samples were freshly prepared to a final volume of 20 µL in disposable 20 µL capacity micropipettes (BlauBrand®), before each EPR measurement. $CuCl_2$ concentration series of 0.1, 0.5, 1.0, 1.5, and 2.5 mM were prepared in either Tris-HCl (20 mM Tris, 50 mM NaCl, pH 7.4), PBS (8.1 mM Na_2HPO_4, 1.5 mM KH_2PO_4, 2.7 mM KCl, 137 mM NaCl, pH 7.3), or MOPS (20 mM MOPS, 50 mM NaCl, pH 7.4) buffers. Additional concentration series were performed (as stated above) for Tris-HCl and PBS buffers, in presence of two molar equivalents of imidazole. A final $CuCl_2$ concentration series (0.05, 0.10, 0.15, 0.20, 0.30, 0.40 mM) was prepared in PBS buffer, in presence of half a molar equivalent of GB1 K28H/Q32H.

1.3 Pulse EPR Measurement Parameters:

All pulse EPR experiments were performed using a Bruker ELEXSYS 580 pulse EPR spectrometer including the second frequency option (E580-400U), operating at 34 GHz (Q-band) equipped with a 3 mm cylindrical resonator in TE012 mode (QT-II). Temperatures were maintained using a cryogen-free variable temperature cryostat (Cryogenic Ltd) operating in the 1.8-300 K temperature range. All measurements of the electron spin longitudinal relaxation times (T_1) of Cu^{II}, and all 5-pulse dead-time free RIDME measurements were performed at 30 K, while all 4-pulse dead-time free PELDOR measurements were performed at 50 K. Metal-nitroxide RIDME and nitroxide-nitroxide PELDOR measurements were performed using a high-power 150 W travelling-wave tube (TWT; Applied Systems Engineering) in a critically- or over-coupled resonator mode, as stated.

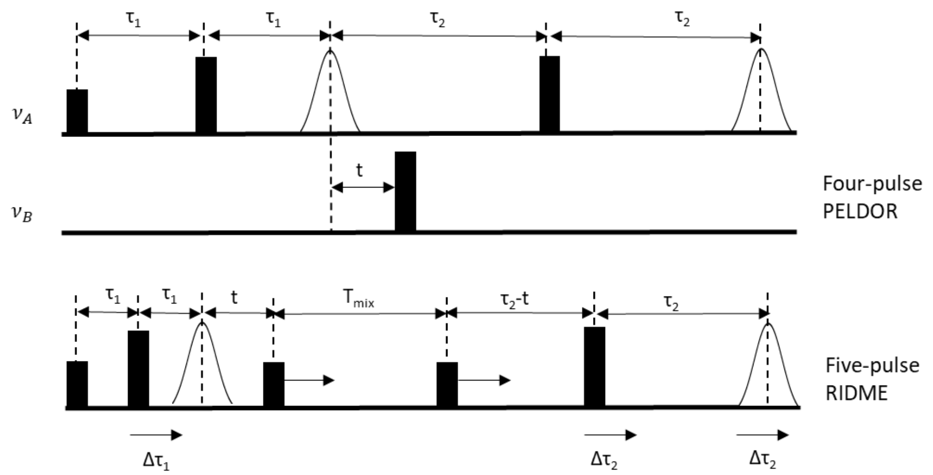

Figure S3. The PELDOR[3] (top) and RIDME[4] (bottom) pulse sequences.

All PELDOR measurements used the 4-pulse double electron-electron resonance (DEER) sequence (π/2(ν_A) – τ_1 – π(ν_A) – (τ_1+ t) – π(ν_B) – (τ_1- t) – π(ν_A) – τ_2 – echo), given in figure S3 top row. Unless otherwise stated, pulse lengths of 16 and 32 ns (π/2 and π) for detection, and 12 ns for the ELDOR π pump pulse were used. A detection position at the maximum of the nitroxide spectrum was used, with a frequency offset (pump – detection frequency) of +80 MHz. Unless otherwise stated, each trace was acquired using a shot repetition time (SRT) of 3.5-5 ms, a τ_1 of 380 ns, a τ_2 of 7000 ns with a dipolar evolution time increment of 12 ns over 598 points, 20 shots-per-point, and varying number of scans, as stated. Deuterium ESEEM was suppressed using a 16-step τ-averaging cycle, and unwanted echoes were suppressed using a 2-step phase cycle, for a total of 32 steps per scan.

All RIDME measurements used the pulse sequence (π/2 – τ_1– π – (τ_1+ t) – π/2 – T_{mix} – π/2 – (τ_2- t) – π – τ_2 – echo), given in figure S3 bottom row, detection pulse lengths of 12 and 24 ns (π/2 and π) and a detection position at the maximum of the nitroxide spectrum. Unless otherwise stated, each trace was acquired using a SRT of 25-30 ms, a τ_1 of 400 ns, a τ_2 of 1300 ns with a dipolar evolution time increment of 12 ns over 122 points, 2 shots-per-point, and varying number of scans, as stated. Deuterium ESEEM was suppressed using a 16-step tau-averaging cycle, and unwanted echoes were suppressed using an 8-step phase cycle, for a total of 128 steps per scan. Each measurement was acquired with a short (T_{ref}) and long (T_{mix}) mixing time, of 5 and 200 μs to allow suppression and observation of the dipolar coupling, respectively.

All inversion recovery (IR) measurements used the pulse sequence (π – T – π/2 – τ_1– π – τ_1–echo). Unless otherwise stated, all IR measurements were performed using detection pulse lengths of 16 and 32 ns (π/2 and π), and an inversion π-pulse length of 32 ns. The detection position was consistently placed at the maximum of the Cu^{II} spectrum. Each trace was acquired using an SRT of 1 ms, with 512 points, 5 shots-per-point, and varying number of scans, as stated. A tau of 200 ns was used, with an approximate time-window length of 600 μs, incremented in steps of 1200 ns. Raw data was fitted with mono- or bi-exponential functions, shown in equations (S1) and (S2), respectively, to estimate T_1.

$$y = M_0 \left[1 - 2\lambda \times \exp\left(\frac{-x}{T_1}\right)\right] \qquad (S1)$$

$$y = M_0 \left[1 - 2\lambda \left(b \times \exp\left(\frac{-x}{T_{1A}}\right) + (1-b)\exp\left(\frac{-x}{T_{1B}}\right)\right)\right] \qquad (S2)$$

where: M_0 corresponds to the signal intensity at $T = \infty$, λ is the inversion efficiency of the π pump pulse, and b is the fractional contribution of the first component in the bi-exponential fit.

1.4 Continuous-Wave EPR Measurement Parameters:

All continuous-wave (CW) EPR measurements were performed using a Bruker EMX 10/12 spectrometer equipped with an ELEXSYS Super Hi-Q resonator, at X-band frequencies (9.8 GHz) and at 298 K. Measurements were performed over 2000-4000 points, sweep-widths of 200-400 mT, with a modulation amplitude of 1 mT, and a conversion time of 40 ms. The number of scans varied between samples, depending on the nominal $CuCl_2$ concentration.

1.5 Pulse Dipolar EPR Data Processing and Validations:

All RIDME and PELDOR data processing and validations were performed using either DeerAnalysis2015, DeerAnalysis2019, or Consensus Deer Analyzer 2.0 as stated. PDF outputs from CDA 2.0 are supplied in the underpinning data. RIDME traces were deconvoluted, (i.e., the traces with longer mixing times were divided by the traces with the shorter mixing time), to improve the quality of the background correction of the data[5] by suppressing a low frequency artefact attributed to dynamic decoupling.[6] Deconvoluted RIDME data was background corrected assuming a stretched exponential background function, with dimension 3-6. PELDOR data was background corrected assuming a background function corresponding to a homogenous distribution in 3 dimensions.

Data analysis was validated by systematic variation of processing parameters as implemented in the DeerAnalysis validation tool. Unless otherwise stated, background dimension and start-time parameters for data processing were determined by an initial validation run, consisting of 56 or 16 trials for RIDME and PELDOR, respectively. For initial RIDME validations, 8 iterations of background start position (between 5-30% of the total RIDME trace length), and 7 iterations of background dimension (between 3-6 in increments of 0.5) were performed. For initial PELDOR validations, 16 iterations of background start position (between 5-80% of the total PELDOR trace length) were performed. Subsequently, a second round of data processing was performed using the background dimension and start-time parameters from the initial validation. This was followed by a second round of validations, consisting of 896 or 800 trials for RIDME and PELDOR, respectively. The validation trials included 16 or 50 white noise iterations (noise level of 1.5), 8 or 16 iterations of background start position (as above) for RIDME and PELDOR, respectively, and an additional 7 iterations of background dimension (as above, apart from the data corresponding to the lower ratio samples, in which case it was varied 4-6 or 5-6) for RIDME data only. These validation trials were

also pruned, where trials exceeding the RMSD of the global minimum by ≥15% were discarded. The regularisation parameter was selected using the L-curve criterion.

The modulation depths from the PELDOR data were normalised by dividing by λ_{max}, ($\lambda_{max} = 0.25$), the approximate inversion efficiency of a rectangular pump pulse for a nitroxide radical at our Q-band setup.[1] The modulation depths from the deconvoluted RIDME data were normalised by dividing by Δ_{Tmix} corrected for deconvolution (Δ_{Tmix}^{corr}):[1]

$$\Delta_{Tmix}^{corr} = 1 - \frac{1 - \Delta_{Tmix}}{1 - \left(\frac{1}{2}\left(1 - exp\left(-\frac{T_{ref}}{T_1}\right)\right)\right)} \qquad (S3)$$

where T_{ref} is the shorter mixing time, and Δ_{Tmix} is defined as:

$$\Delta_{Tmix} = \frac{1}{2} \times \left(1 - exp\left(-\frac{T_{mix}}{T_1}\right)\right) \qquad (S4)$$

where T_1 is the longitudinal relaxation time of Cu^{II} for each sample, and T_{mix} is the longer mixing time. The expression (S3) accounts for the loss of dipolar coupling that arises from deconvolution. Error bars for the RIDME modulation depth quotients were calculated as previously described,[7] while error bars for the PELDOR modulation depth quotients were calculated by taking the RMSD of the third quartile of the imaginary component of the dipolar evolution function.

1.6 Continuous-Wave EPR Data Processing:

All CW-EPR spectra were firstly phase-corrected, before spectra of the capillary signal (filled with appropriate buffer) and sample spectra were baseline-corrected assuming a third-order polynomial background for subtraction. The baseline-corrected capillary signal spectra were subsequently subtracted from the phase-corrected, baseline-corrected sample spectra. These spectra were then integrated, baseline-corrected (assuming a third-order polynomial background for subtraction) and integrated a second time, to yield the corresponding double-integral spectra. The double integrals were calculated as the intensity values at a field position of 360 mT for all concentration series, except the series performed in presence of PBS buffer and K28H/Q32H GB1 protein, which used intensity values at a field position of 352.7 mT.

1.7 Analytical Cooperative Binding Model:

The following model is adapted from the supporting information of Mack *et al.*[8] wherein binding equilibria between a bivalent ligand (in our case Cu^{II}) and monomeric protein (in our case GB1) are mathematically modelled. There are two main assumptions of the model: i) the template is bivalent, and can bind two protein monomers simultaneously, ii) the two binding events are considered dependent, and this is subsumed into a cooperativity factor, α.

Let us begin by considering an initial association reaction for the GB1, Cu^{II} system (S5):

$$GB1 + Cu^{II} \rightleftharpoons GB1 - Cu^{II} \qquad (S5)$$

Where the dissociation constant (K_D) for a single binding event, for a monovalent template, is described as:

$$K_D = \frac{[GB1][Cu^{II}]}{[GB1 - Cu^{II}]} \qquad (S6)$$

Owing to the first assumption, that the template is bivalent, there are two ways to form the protein-template complex (i.e., the protein can bind at site A or B on the template), but only one way to dissociate into the constituent protein and template. This manifests as a statistical factor of 2 in the association constant K_A, and as a statistical factor of 1/2 in K_D. Therefore equation (S6) can be reframed to account for the bivalency of the template:

$$\frac{K_D}{2} = \frac{[GB1][Cu^{II}]}{[GB1 - Cu^{II}]} \qquad (S7)$$

This can be re-arranged to yield an expression for $[GB1 - Cu^{II}]$ given below in equation (S8):

$$[GB1 - Cu^{II}] = \frac{2[GB1][Cu^{II}]}{K_D} \qquad (S8)$$

The second binding event can be considered by the reaction scheme (S9):

$$GB1 + GB1 - Cu^{II} \rightleftharpoons GB1 - Cu^{II} - GB1 \qquad (S9)$$

Here, for a bivalent template, there is only one way to form the protein-template-protein complex, but two ways to dissociate into the constituent protein and protein-template (*i.e.,* protein can dissociate from either site A or site B on the template). This manifests as a statistical pre-factor of 1/2 in K_A, and as a statistical pre-factor of 2 in K_D. Owing to the second assumption, for the second binding event, the additional pre-factor α^{-1} accounts for cooperativity of binding. For $\alpha > 1$, cooperativity is positive, and the K_D will be reduced for the second binding event, while for $\alpha < 1$, cooperativity is negative, and K_D will be increased for the second binding event. In the case of $\alpha =$

1, the binding events are independent (*i.e.,* non-cooperative). Taking these considerations into account, we can write:

$$\frac{2K_D}{\alpha} = \frac{[GB1][GB1 - Cu^{II}]}{[GB1 - Cu^{II} - GB1]} \qquad (S10)$$

This can be re-arranged to yield an expression for $[GB1 - Cu^{II} - GB1]$ given below in equation (S11):

$$[GB1 - Cu^{II} - GB1] = \frac{\alpha[GB1][GB1 - Cu^{II}]}{2K_D} \qquad (S11)$$

Substitution of equation (S8) into equation (S11) yields an expression for $[GB1 - Cu^{II} - GB1]$ in terms of K_D, α, $[GB1]$, and $[Cu^{II}]$, given as

$$[GB1 - Cu^{II} - GB1] = \frac{\alpha[Cu^{II}][GB1]^2}{{K_D}^2} \qquad (S12)$$

With these definitions one can define total Cu^{II} concentration, $[Cu^{II}]_0$ as follows

$$[Cu^{II}]_0 = [Cu^{II}] + \frac{2[GB1][Cu^{II}]}{K_D} + \frac{\alpha[Cu^{II}][GB1]^2}{{K_D}^2} \qquad (S13)$$

Factorizing with respect to $[Cu^{II}]$ yields

$$[Cu^{II}]_0 = [Cu^{II}]\left(1 + \frac{2[GB1]}{K_D} + \frac{\alpha[GB1]^2}{{K_D}^2}\right) \qquad (S14)$$

Rearranging to give an expression for $[Cu^{II}]$ gives

$$[Cu^{II}] = \frac{[Cu^{II}]_0}{\left(1 + \frac{2[GB1]}{K_D} + \frac{\alpha[GB1]^2}{{K_D}^2}\right)} = \frac{[Cu^{II}]_0 {K_D}^2}{{K_D}^2 + 2[GB1]K_D + \alpha[GB1]^2} \qquad (S15)$$

This expression can then be substituted into equations (S8) and (S11), respectively, to yield equations (S16) and (S17) below:

$$[GB1 - Cu^{II}] = \frac{2[GB1][Cu^{II}]_0 K_D}{{K_D}^2 + 2[GB1]K_D + \alpha[GB1]^2} \qquad (S16)$$

$$[GB1 - Cu^{II} - GB1] = \frac{\alpha[Cu^{II}]_0[GB1]^2}{{K_D}^2 + 2[GB1]K_D + \alpha[GB1]^2} \qquad (S17)$$

With these definitions one can define total protein concentration, $[GB1]_0$ as follows:

$$[GB1]_0 = [GB1] + \frac{2[GB1][Cu^{II}]_0 K_D}{{K_D}^2 + 2[GB1]K_D + \alpha[GB1]^2} + \frac{2\alpha[Cu^{II}]_0[GB1]^2}{{K_D}^2 + 2[GB1]K_D + \alpha[GB1]^2} \qquad (S18)$$

This can be rearranged to yield a cubic equation in $[GB1]$

$$0 = ([GB1]_0 - [GB1])\left({K_D}^2 + 2[GB1]K_D + \alpha[GB1]^2\right) - 2[GB1][Cu^{II}]_0K_D - 2\alpha[Cu^{II}]_0[GB1]^2 \quad (S19)$$

Solving analytically yields the cubic equation

$$0 = [GB1]^3 + a[GB1]^2 + b[GB1] + c \quad (S20)$$

where:

$$a = \frac{2K_D}{\alpha} + 2[Cu^{II}]_0 - [GB1]_0 \quad (S21)$$

$$b = \frac{K_D}{\alpha}(K_D + 2[Cu^{II}]_0 - 2[GB1]_0) \quad (S22)$$

$$c = \frac{-(K_D)^2}{\alpha}[GB1]_0 \quad (S23)$$

The physically real root of this cubic equation is given as

$$[GB1] = -\frac{a}{3} + \sqrt[3]{\left(R + \sqrt{Q^3 + R^2}\right)} + \sqrt[3]{\left(R - \sqrt{Q^3 + R^2}\right)} \quad (S24)$$

where a is defined as above, and Q and R are defined below in (S25) and (S26), respectively:

$$Q = \frac{3b - a^2}{9} \quad (S25)$$

$$R = \frac{9ab - 27c - 2a^3}{54} \quad (S26)$$

The benefit of using this approach to analytically calculate the equilibrium concentration of $[GB1]$, is that it allows downstream bivariate fitting of both an apparent K_D and cooperativity factor α. Finally, using expressions (S16-S26) we can relate $[GB1 - Cu^{II}]$ and $[GB1 - Cu^{II} - GB1]$ to the modulation depths for RIDME ($\Delta_{Cu^{II}-R1}$) and DEER (Δ_{R1-R1}) as follows:

$$\frac{\Delta_{Cu^{II}-R1}}{\Delta_{Tmix}} = \frac{[GB1 - Cu^{II}]}{[GB1]_0} + \frac{[GB1 - Cu^{II} - GB1]}{[GB1]_0} \quad (S27)$$

$$\frac{\Delta_{R1-R1}}{\lambda_{max}} = \frac{[GB1 - Cu^{II} - GB1]}{[GB1]_0} \quad (S28)$$

where Δ_{Tmix} is defined above in (S4), and $\lambda_{max} = 0.25$, as discussed in section 1.5. Equations (S27) and (S28) can be rationalised by first considering that the RIDME experiment detects the R1 nitroxide, and so is sensitive to all GB1 protein (assuming quantitative spin labelling and absence of free spin label). Therefore, both Cu^{II}-bound monomeric GB1 and Cu^{II}-templated dimeric GB1 species will be detected and contribute to the modulation depth (i.e., dipolar coupling between the R1 nitroxide and the Cu^{II} centre). Similarly, the DEER experiment detects the R1 nitroxide, however only the Cu^{II}-templated dimeric GB1 species will contribute to the modulation depth (i.e., dipolar

coupling between pairs of R1 nitroxide only). Substituting equations (S15-S16) into the numerators of equations (S27-S28) respectively, gives modulation depths in terms of the thermodynamic parameters K_D and α, and total and equilibrium concentrations of Cu^{II} and GB1, respectively:

$$\frac{\Delta_{Cu^{II}-R1}}{\Delta_{Tmix}} = \frac{2[Cu^{II}]_0 K_D}{\alpha[GB1]^2 + 2[GB1]K_D + 2[Cu^{II}]_0\alpha[GB1] + {K_D}^2 + 2[Cu^{II}]_0 K_D} + \frac{\alpha[GB1][Cu^{II}]_0}{\alpha[GB1]^2 + 2[GB1]K_D + 2[Cu^{II}]_0\alpha[GB1] + {K_D}^2 + 2[Cu^{II}]_0 K_D} \quad (S29)$$

$$\frac{\Delta_{R1-R1}}{\lambda_{max}} = \frac{\alpha[GB1][Cu^{II}]_0}{\alpha[GB1]^2 + 2[GB1]K_D + 2[Cu^{II}]_0\alpha[GB1] + {K_D}^2 + 2[Cu^{II}]_0 K_D} \quad (S30)$$

1.8 In Silico Modelling of Metal Templated Dimer:

The I6H/N8H/K28H/Q32H GB1 monomer structure (PDB: 4WH4[9]) was used as a starting point to model the metal templated dimer. Firstly, histidine residues 6H and 8H were mutated to cysteine (C) and asparagine (N), respectively, using the mutagenesis tool in Pymol (The Pymol Molecular Graphics System, Version 2.0 Schrödinger, LLC). XPLOR molecular dynamics simulations were performed, as previously described,[1] to build on the Cu^{II}-IDA spin probe (at residue positions 28 and 32). The atoms of the IDA chelating agent were then removed, before a second I6C/K28H/Q32H GB1 monomer was coordinated around the Cu^{II} centre, in approximately square-planar geometry. MTSL spin labels (constituting the R1 sidechains) were then introduced at position 6 for each monomer in the putative dimer structure with either the MTSSLWizard[10] Pymol plugin or MMM2018.[11] For modelling of the nitroxide-nitroxide distance distributions in MTSSLWizard[10], the PDB of the putative dimer structure was used as a template. Conformers of the R1 sidechain were calculated using the 'painstaking' search setting. In MMM2018, the R1 sidechain was introduced using the 'site-scan' option, assuming labelling under ambient (298 K) temperature conditions. The corresponding *in silico* distance distributions (compared with experimental PELDOR data) are shown in the results and discussion (section 2.6).

II **Results and Discussion:**

2.1 Screening of Dimer Formation for Different Constructs and Templates:

As an initial screening of dimer formation by the two GB1 constructs used in this work (I6R1/K28H/Q32H and I6H/N8H/K28R1), nitroxide-nitroxide PELDOR measurements were performed in presence of metal ions: Cu^{II} and Zn^{II}, at a 1:2 metal:protein ratio. Figures S4-S5 show the PELDOR primary data for the I6H/N8H/K28R1 and I6R1/K28H/Q32H constructs, respectively. Background correction parameters for the I6R1/K28H/Q32H construct measurements are given in table S1. The low modulation depth (below 1%) for both Cu^{II} and Zn^{II} in figure S4 indicates that dimerization is not observed across the sheet-face of the protein. Conversely, significant modulation depths are observed for both Cu^{II} and Zn^{II} in figure S5, suggesting dimerization is favoured on the helix-face of the protein.

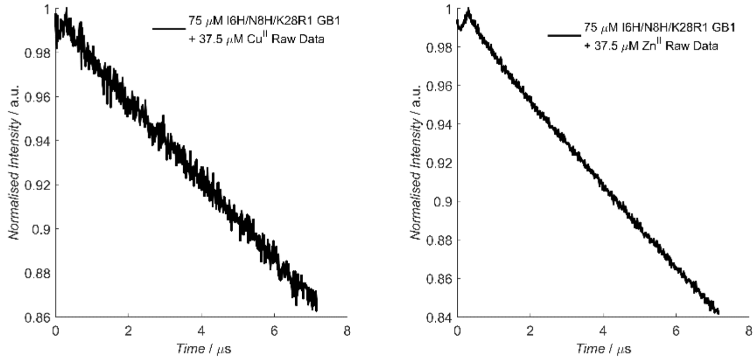

Figure S4. Q-band nitroxide-nitroxide PELDOR raw data for the construct I6H/N8H/K28R1, in presence of Cu^{II} (left) and Zn^{II} (right) at a metal:protein ratio of 1:2. These measurements used an ELDOR π-pulse length of 20 ns.

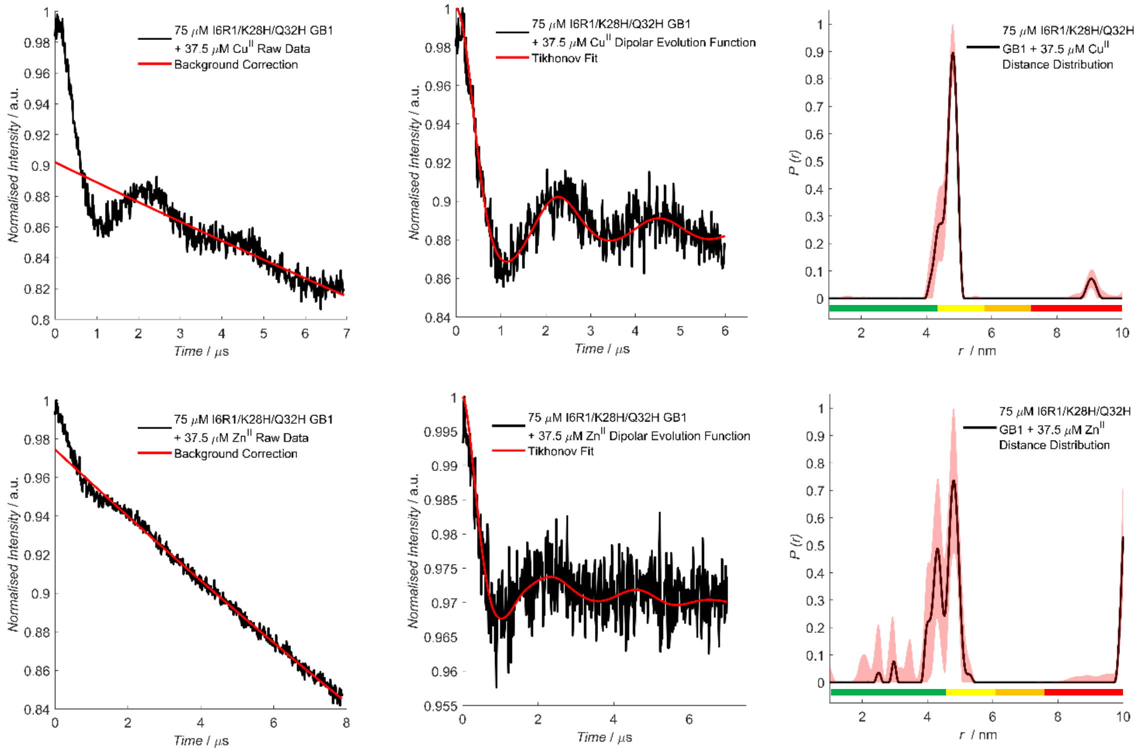

Figure S5. Validated Q-band nitroxide-nitroxide PELDOR data for the construct I6R1/K28H/Q32H, in presence of Cu^{II} (top row) and Zn^{II} (bottom row) at a metal:protein ratio of 1:2. PELDOR raw time trace, background corrected trace, and validated distance distribution are shown left-to-right, respectively. Colour bars indicate regions of the distance distributions that are shape reliable (green), mean distance and width reliable (yellow), mean distance reliable (orange), and no quantification possible (red). These measurements used an ELDOR π-pulse length of 20 ns, and the Zn^{II} sample was measured with a τ_2 = 8 µs.

Sample	Zero-time [ns]	Background Start [ns]	Background Cutoff [ns]	Regularization Parameter (α)	Modulation Depth (Δ)
6R1/28H/32H + Cu^{II}	232	1200	6000	10	0.098
6R1/28H/32H + Zn^{II}	276	5250	7000	10	0.025

Table S1. Background correction parameters and modulation depths corresponding to the Q-band nitroxide-nitroxide PELDOR data shown in figure S5.

The observed preference for dimer formation across the α-helical face of GB1 can be rationalised by considering steric arguments. The α-helix provides a convex surface on which to first bind metal template, and allow docking of a second GB1 monomer, while the β-sheet instead has residues occupying configurations approximately normal to the plane of the sheet. It has also been shown

extensively that Cu^{II}-chelates bind more efficiently to double-histidine motifs in an α-helical secondary structure, compared to a β-sheet secondary structure.[1,7] The non-negligible modulation depth observed in figure S4 likely arises from minor deviations from a three-dimensional homogeneous distribution in the sample, assumed for a glassy frozen solution, and the presence of excluded volumes limiting the closest approach of spin labels. This has been shown to induce a small apparent modulation depth after background correction.[5] Finally, the larger modulation depth observed in figure S5 for Cu^{II} compared to Zn^{II} can be explained owing to the tighter binding affinity of Cu^{II} for histidine residues, per the Irving-Williams series.

Since Zn^{II} is diamagnetic, and a larger propensity for dimerization was observed at the α-helical face when using Cu^{II} as the metal template, Cu^{II} and the I6R1/K28H/Q32H construct were taken forward for further characterization by pulse dipolar EPR.

2.2 Inversion Recovery Measurements:

Inversion recovery measurements were performed to estimate the longitudinal relaxation time (T_1) of the Cu^{II}, and the raw data is shown in figures S6-7, for the Tris and phosphate buffer conditions, respectively. The corresponding mono- and bi-exponential fits are shown as red and blue traces, respectively. The estimates of T_1 fitted under the mono- and bi-exponential approximations, as well as the reciprocal e-times are given in tables S2-3.

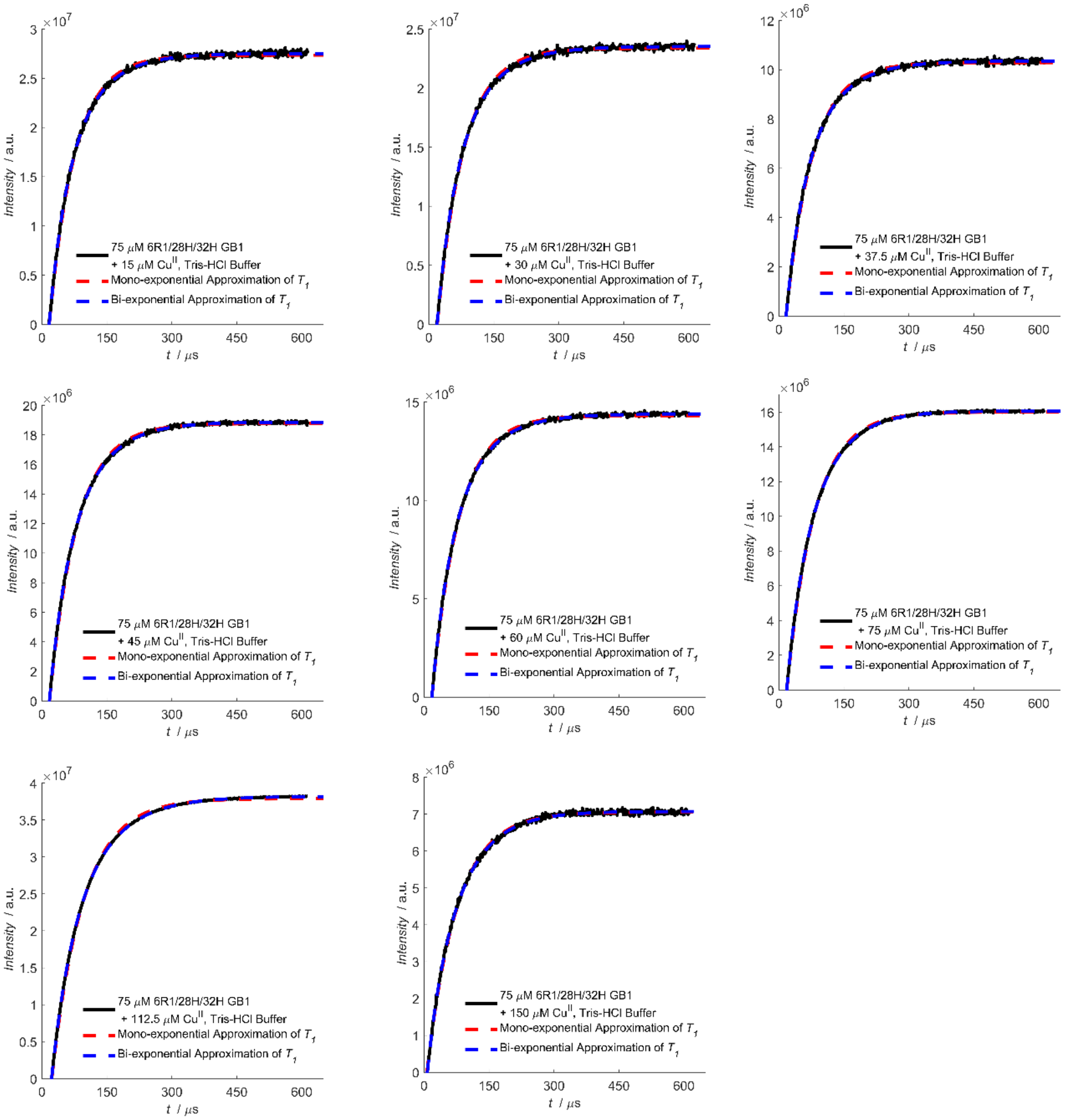

Figure S6. Inversion recovery data in Tris-HCl buffer (20 mM Tris-HCl, 150 mM NaCl, pH 7.4) for 15.0, 30.0, and 37.5 μM Cu^{II} (top row), 45.0, 60.0, and 75.0 μM Cu^{II} (middle row), and 112.5, and 150 μM Cu^{II} (bottom row) in presence of 75 μM I6R1/K28H/Q32H GB1 shown left-to-right, respectively. The experimental data is shown in black, with the mono-exponential and bi-exponential fits shown as red and blue dotted lines, respectively.

Sample	Mono-exponential T_1 [µs]	Bi-exponential T_1 [µs]	1/e time [µs]
75 µM 6R1/28H/32H + 15.0 µM Cu^{II}	59.1 ± 0.79	39.4 (0.48) / 72.7 (0.52)	32.8
75 µM 6R1/28H/32H + 30.0 µM Cu^{II}	61.0 ± 0.80	41.4 (0.50) / 83.0 (0.50)	35.2
75 µM 6R1/28H/32H + 37.5 µM Cu^{II}	60.9 ± 0.80	41.2 (0.51) / 82.9 (0.49)	34.6
75 µM 6R1/28H/32H + 45.0 µM Cu^{II}	63.6 ± 0.63	36.1 (0.31) / 76.4 (0.69)	37.6
75 µM 6R1/28H/32H + 60.0 µM Cu^{II}	61.6 ± 0.61	42.4 (0.48) / 80.7 (0.52)	36.4
75 µM 6R1/28H/32H + 75.0 µM Cu^{II}	63.6 ± 0.51	30.8 (0.21) / 72.0 (0.79)	40.0
75 µM 6R1/28H/32H + 112.5 µM Cu^{II}	72.7 ± 0.73	43.1 (0.38) / 92.3 (0.62)	43.6
75 µM 6R1/28H/32H + 150.0 µM Cu^{II}	69.2 ± 0.88	24.0 (0.12) / 74.5 (0.88)	27.4

Table S2: Mono- and bi-exponential T_1 estimates, and 1/e times for the inversion recovery data shown in figure S6.

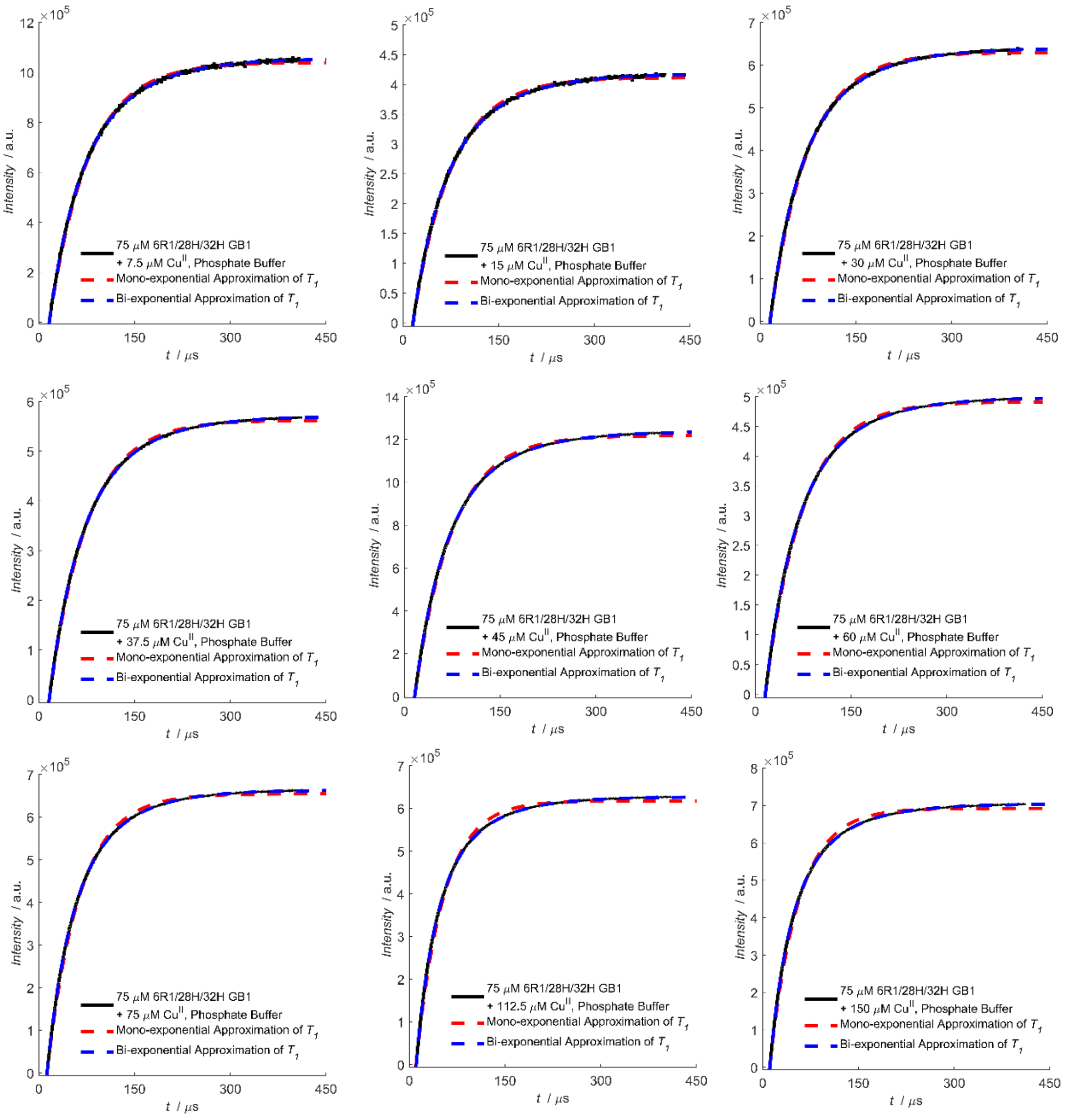

Figure S7. Inversion recovery data in phosphate buffer (42.4 mM Na_2HPO_4 and 7.6 mM KH_2PO_4, 150 mM NaCl, pH 7.4) for 7.5, 15.0, and 30.0 μM Cu^{II} (top row), 37.5, 45.0, and 60.0 μM Cu^{II} (middle row), and 75.0, 112.5, and 150 μM Cu^{II} (bottom row) in presence of 75 μM I6R1/K28H/Q32H GB1 shown left-to-right, respectively. The experimental data is shown in black, with the mono-exponential and bi-exponential fits shown as red and blue dotted lines, respectively.

Sample	Mono-exponential T_1 [µs]	Bi-exponential T_1 [µs]	1/e time [µs]
75 µM 6R1/28H/32H + 7.5 µM Cu^{II}	61.4 ± 0.38	41.0 (0.52) / 89.6 (0.48)	34.2
75 µM 6R1/28H/32H + 15.0 µM Cu^{II}	60.6 ± 0.39	40.7 (0.54) / 90.3 (0.46)	33.4
75 µM 6R1/28H/32H + 30.0 µM Cu^{II}	59.3 ± 0.36	40.7 (0.57) / 91.4 (0.43)	33.2
75 µM 6R1/28H/32H + 37.5 µM Cu^{II}	59.5 ± 0.31	41.1 (0.55) / 87.4 (0.45)	33.6
75 µM 6R1/28H/32H + 45.0 µM Cu^{II}	58.2 ± 0.33	40.3 (0.58) / 90.0 (0.42)	33.0
75 µM 6R1/28H/32H + 60.0 µM Cu^{II}	58.7 ± 0.34	39.1 (0.53) / 86.6 (0.47)	33.0
75 µM 6R1/28H/32H + 75.0 µM Cu^{II}	50.6 ± 0.37	30.3 (0.49) / 73.4 (0.51)	27.2
75 µM 6R1/28H/32H + 112.5 µM Cu^{II}	42.8 ± 0.43	25.8 (0.59) / 72.1 (0.41)	22.4
75 µM 6R1/28H/32H + 150.0 µM Cu^{II}	44.5 ± 0.45	26.6 (0.58) / 74.4 (0.42)	23.4

Table S3: Mono- and bi-exponential T_1 estimates, and 1/e times for the inversion recovery data shown in figure S7.

2.3 Validated PELDOR and RIDME Measurements:

2.3.1 Tris Buffer Series:

An 8-point pseudo-titration series (metal:protein ratios 1:5, 2:5, 1:2, 3:5, 4:5, 1:1, 3:2, and 2:1) was prepared in Tris-HCl buffer (20 mM Tris-HCl, 150 mM NaCl, pH 7.4) and Cu^{II}-nitroxide RIDME and nitroxide-nitroxide PELDOR measurements were performed. These RIDME measurements were performed using an over-coupled resonator mode. Validations were performed as described in section 1.5, with validated RIDME and PELDOR traces shown in figures S8-S15, and S16-S23, respectively. Colour bars indicate regions of the distance distributions that are shape reliable (green), mean distance and width reliable (yellow), mean distance reliable (orange), and no quantification possible (red). The background correction parameters, and modulation depths are given in tables S4-S5, respectively.

Cu^{II}-nitroxide RIDME:

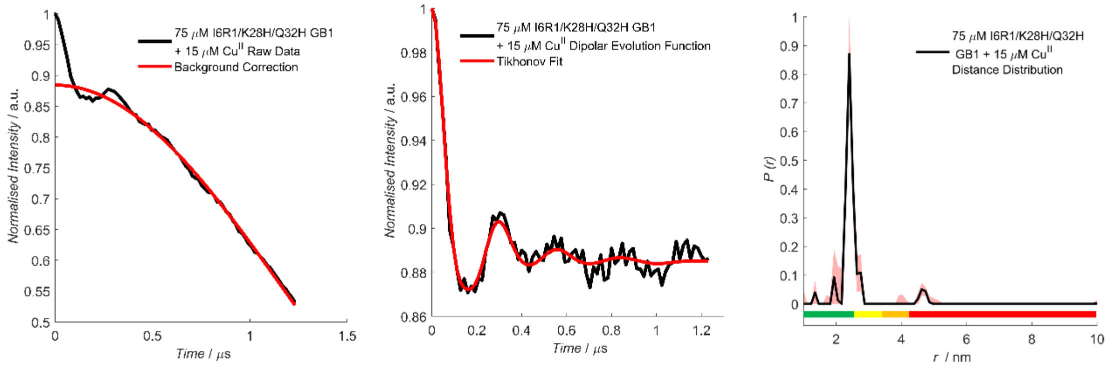

Figure S8. Validated Q-band Cu^{II}-nitroxide RIDME data for the construct I6R1/K28H/Q32H, in presence of Tris-HCl buffer, and Cu^{II} at a metal:protein ratio of 1:5. RIDME raw time trace, background corrected trace, and validated distance distribution are shown left-to-right, respectively.

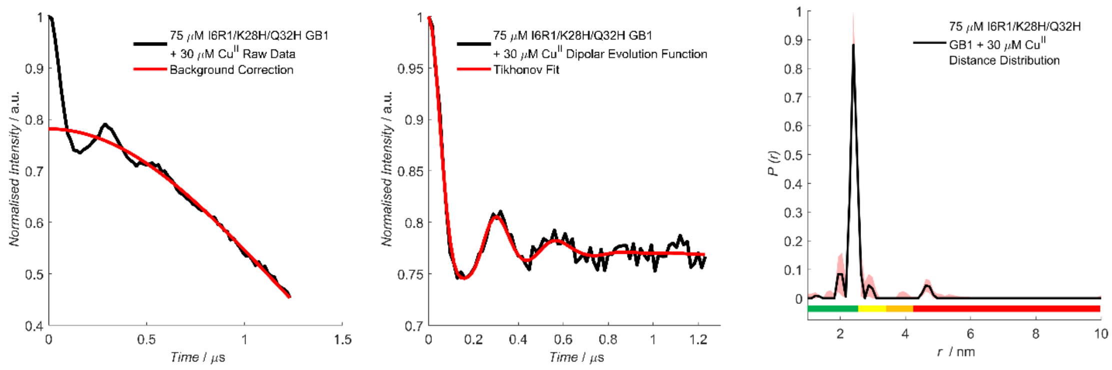

Figure S9. Validated Q-band Cu^{II}-nitroxide RIDME data for the construct I6R1/K28H/Q32H, in presence of Tris-HCl buffer, and Cu^{II} at a metal:protein ratio of 2:5. RIDME raw time trace, background corrected trace, and validated distance distribution are shown left-to-right, respectively.

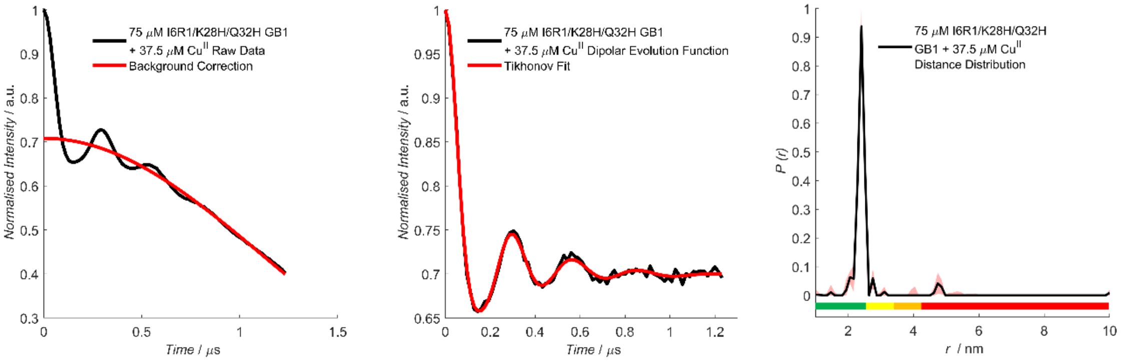

Figure S10. Validated Q-band Cu^{II}-nitroxide RIDME data for the construct I6R1/K28H/Q32H, in presence of Tris-HCl buffer, and Cu^{II} at a metal:protein ratio of 1:2. RIDME raw time trace, background corrected trace, and validated distance distribution are shown left-to-right, respectively.

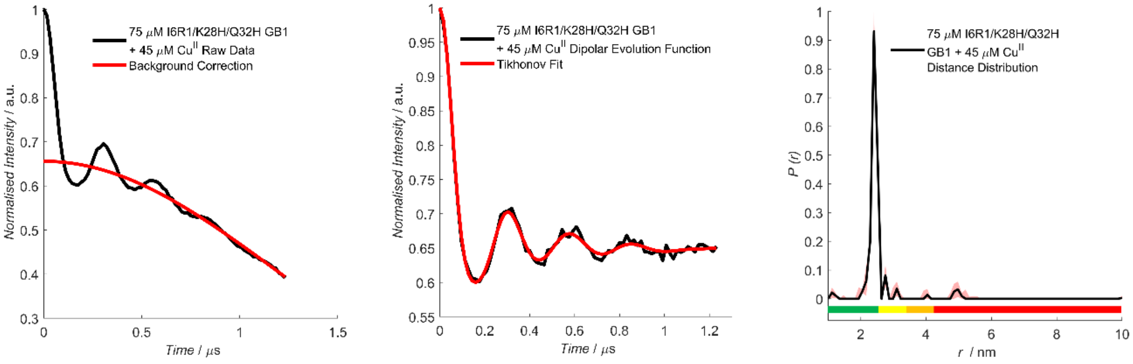

Figure S11. Validated Q-band Cu^{II}-nitroxide RIDME data for the construct I6R1/K28H/Q32H, in presence of Tris-HCl buffer, and Cu^{II} at a metal:protein ratio of 3:5. RIDME raw time trace, background corrected trace, and validated distance distribution are shown left-to-right, respectively.

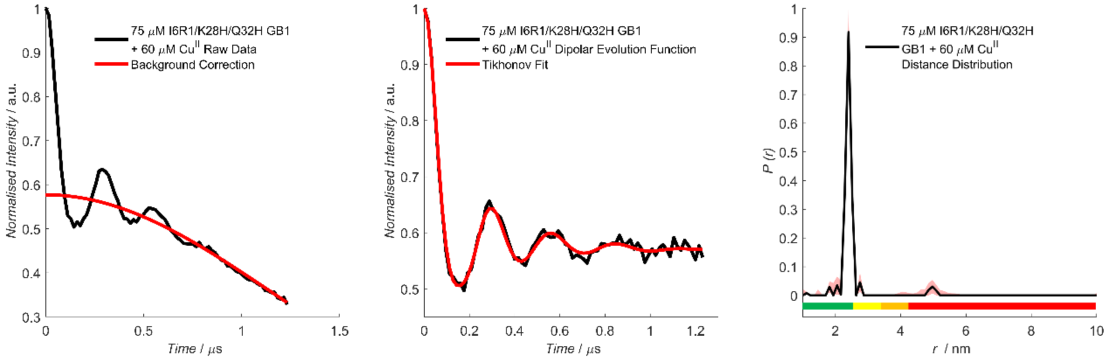

Figure S12. Validated Q-band Cu^{II}-nitroxide RIDME data for the construct I6R1/K28H/Q32H, in presence of Tris-HCl buffer, and Cu^{II} at a metal:protein ratio of 4:5. RIDME raw time trace, background corrected trace, and validated distance distribution are shown left-to-right, respectively.

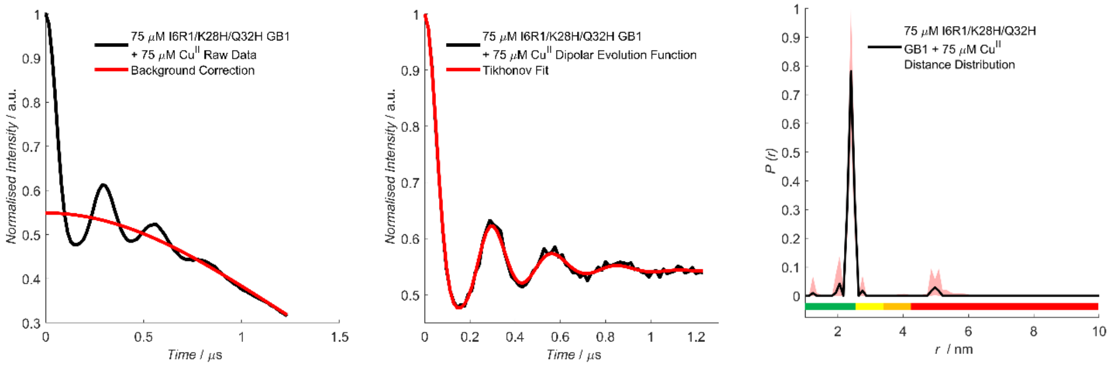

Figure S13. Validated Q-band Cu^{II}-nitroxide RIDME data for the construct I6R1/K28H/Q32H, in presence of Tris-HCl buffer, and Cu^{II} at a metal:protein ratio of 1:1. RIDME raw time trace, background corrected trace, and validated distance distribution are shown left-to-right, respectively.

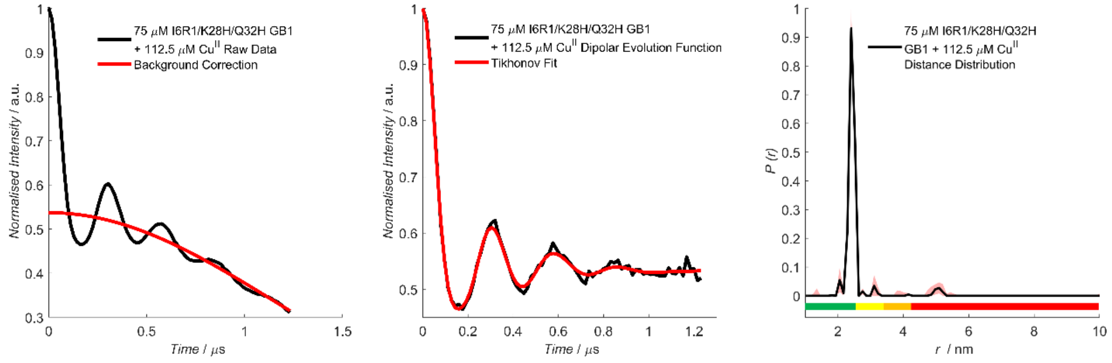

Figure S14. Validated Q-band Cu^{II}-nitroxide RIDME data for the construct I6R1/K28H/Q32H, in presence of Tris-HCl buffer, and Cu^{II} at a metal:protein ratio of 3:2. RIDME raw time trace, background corrected trace, and validated distance distribution are shown left-to-right, respectively.

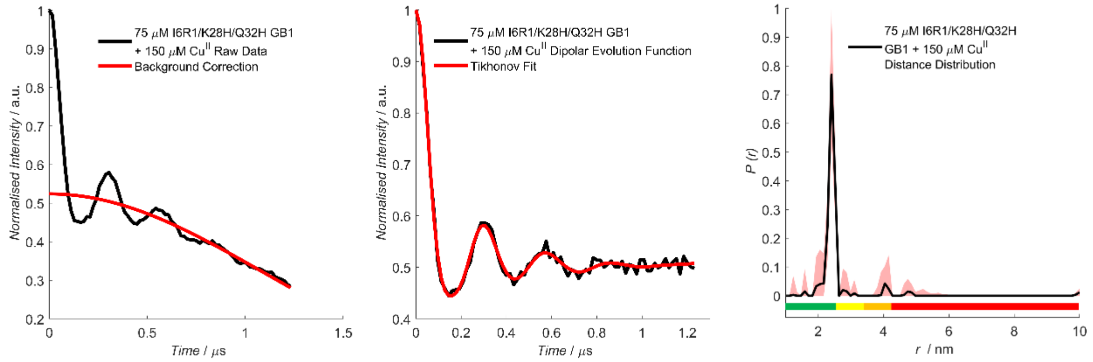

Figure S15. Validated Q-band Cu^{II}-nitroxide RIDME data for the construct I6R1/K28H/Q32H, in presence of Tris-HCl buffer, and Cu^{II} at a metal:protein ratio of 2:1. RIDME raw time trace, background corrected trace, and validated distance distribution are shown left-to-right, respectively.

Sample	Zero-time [ns]	Background Start [ns]	Background Cutoff [ns]	Background Dimension	Modulation Depth (Δ)	Regularization Parameter (α)
75 µM 6R1/28H/32H + 15.0 µM Cu^{II}	212	62	1232	6	0.116	1
75 µM 6R1/28H/32H + 30.0 µM Cu^{II}	213	150	1232	6	0.218	1
75 µM 6R1/28H/32H + 37.5 µM Cu^{II}	211	369	1232	6	0.292	0.1
75 µM 6R1/28H/32H + 45.0 µM Cu^{II}	210	370	1232	6	0.344	0.1
75 µM 6R1/28H/32H + 60.0 µM Cu^{II}	212	282	1232	6	0.423	0.1
75 µM 6R1/28H/32H + 75.0 µM Cu^{II}	210	370	1232	6	0.451	0.1
75 µM 6R1/28H/32H + 112.5 µM Cu^{II}	208	150	1232	6	0.463	0.1
75 µM 6R1/28H/32H + 150.0 µM Cu^{II}	214	370	1232	6	0.470	0.1

Table S4: Background correction parameters and modulation depths for the RIDME data shown in figures S8-S15.

Nitroxide-nitroxide PELDOR:

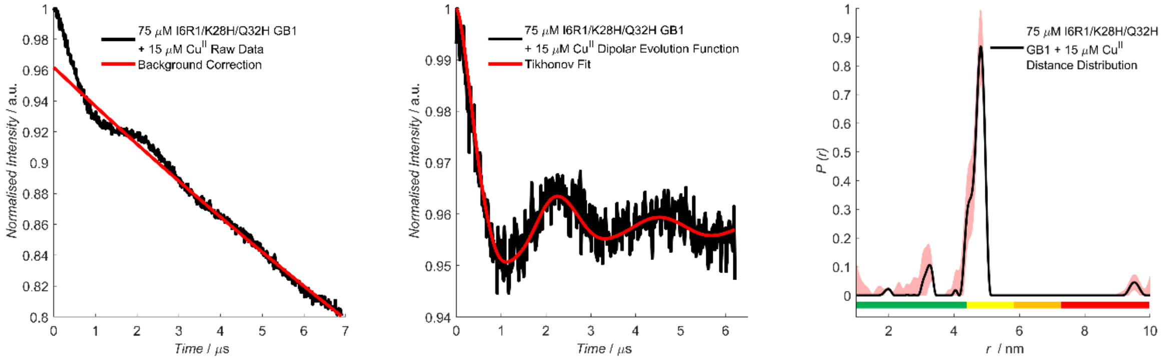

Figure S16. Validated Q-band nitroxide-nitroxide PELDOR data for the construct I6R1/K28H/Q32H, in presence of Tris-HCl buffer, and Cu^{II} at a metal:protein ratio of 1:5. PELDOR raw time trace, background corrected trace, and validated distance distribution are shown left-to-right, respectively.

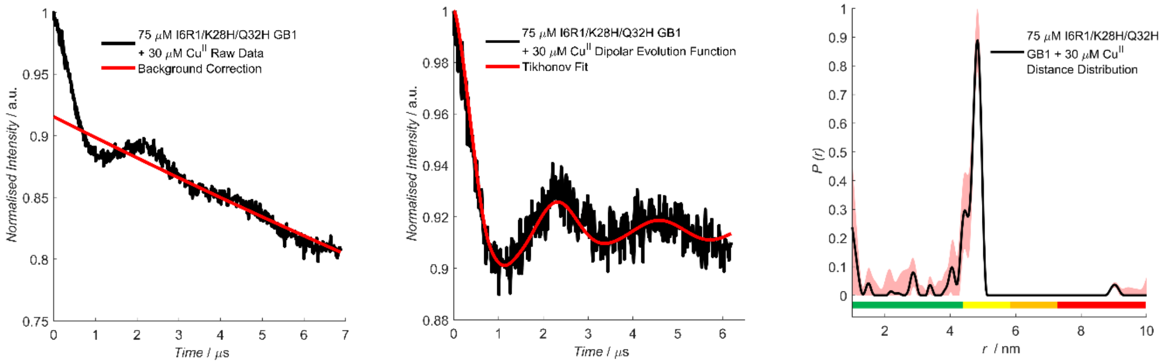

Figure S17. Validated Q-band nitroxide-nitroxide PELDOR data for the construct I6R1/K28H/Q32H, in presence of Tris-HCl buffer, and Cu^{II} at a metal:protein ratio of 2:5. PELDOR raw time trace, background corrected trace, and validated distance distribution are shown left-to-right, respectively.

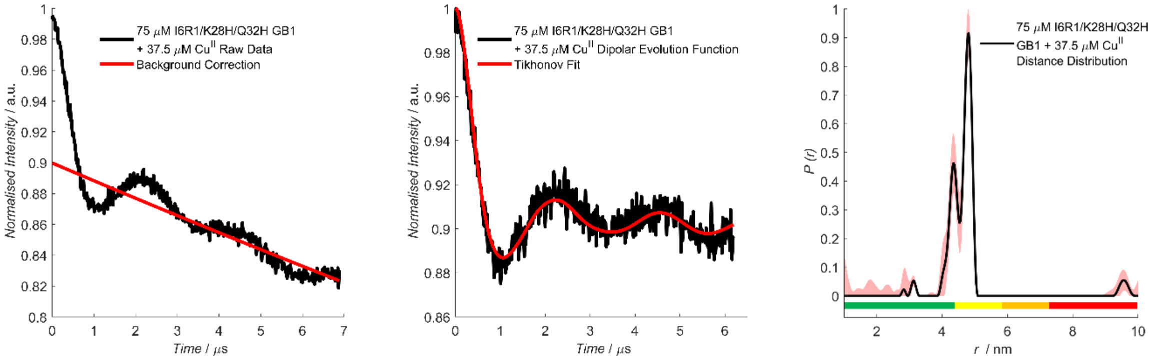

Figure S18. Validated Q-band nitroxide-nitroxide PELDOR data for the construct I6R1/K28H/Q32H, in presence of Tris-HCl buffer, and Cu^{II} at a metal:protein ratio of 1:2. PELDOR raw time trace, background corrected trace, and validated distance distribution are shown left-to-right, respectively.

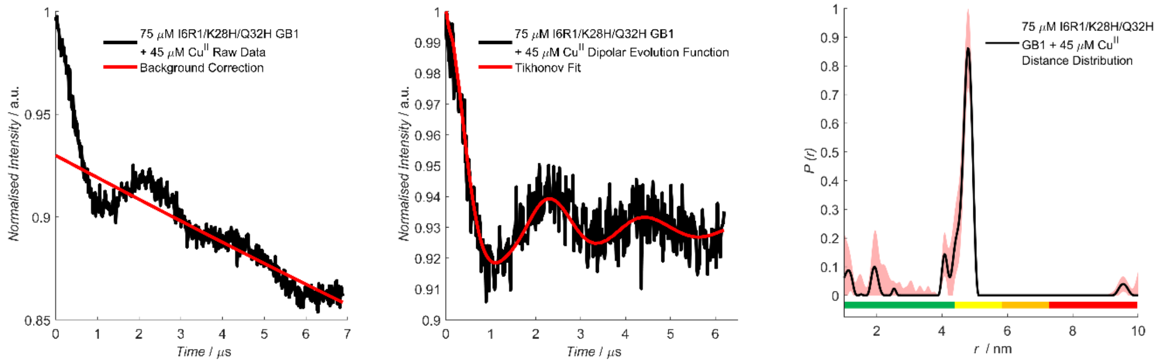

Figure S19. Validated Q-band nitroxide-nitroxide PELDOR data for the construct I6R1/K28H/Q32H, in presence of Tris-HCl buffer, and Cu^{II} at a metal:protein ratio of 3:5. PELDOR raw time trace, background corrected trace, and validated distance distribution are shown left-to-right, respectively.

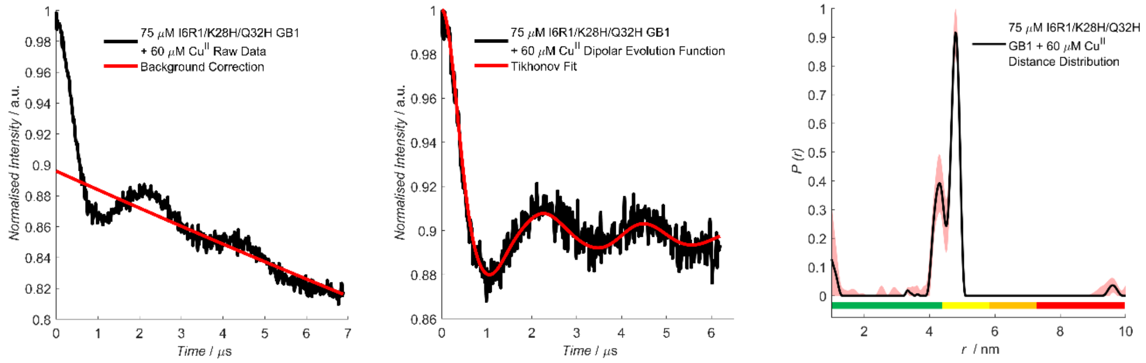

Figure S20. Validated Q-band nitroxide-nitroxide PELDOR data for the construct I6R1/K28H/Q32H, in presence of Tris-HCl buffer, and Cu^{II} at a metal:protein ratio of 4:5. PELDOR raw time trace, background corrected trace, and validated distance distribution are shown left-to-right, respectively.

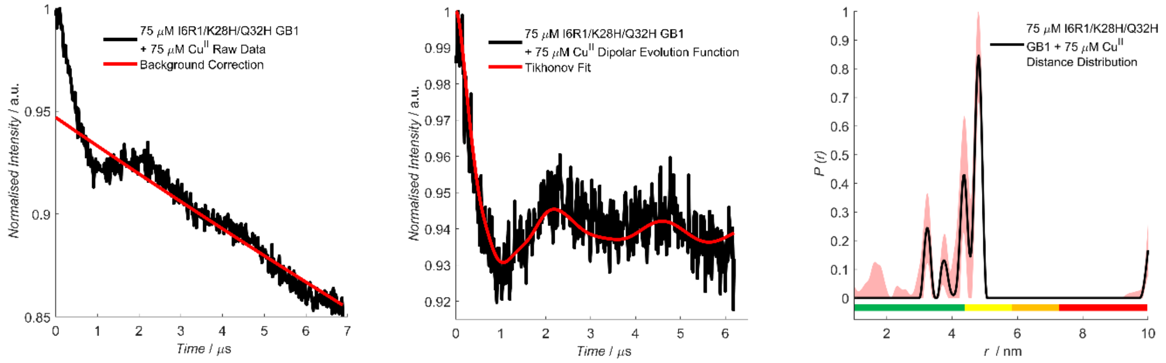

Figure S21. Validated Q-band nitroxide-nitroxide PELDOR data for the construct I6R1/K28H/Q32H, in presence of Tris-HCl buffer, and Cu^{II} at a metal:protein ratio of 1:1. PELDOR raw time trace, background corrected trace, and validated distance distribution are shown left-to-right, respectively.

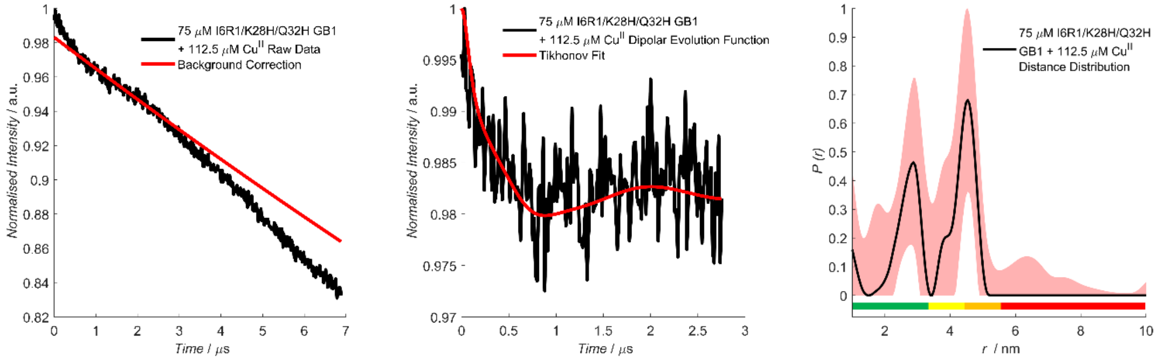

Figure S22. Validated Q-band nitroxide-nitroxide PELDOR data for the construct I6R1/K28H/Q32H, in presence of Tris-HCl buffer, and Cu^{II} at a metal:protein ratio of 3:2. PELDOR raw time trace, background corrected trace, and validated distance distribution are shown left-to-right, respectively.

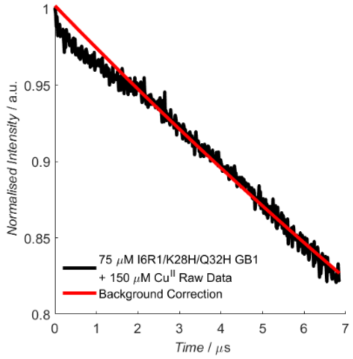

Figure S23. Q-band nitroxide-nitroxide PELDOR data for the construct I6R1/K28H/Q32H, in presence of Tris-HCl buffer, and Cu^{II} at a metal:protein ratio of 2:1. Only the PELDOR raw time trace is shown, indicating a monotonous background decay without modulated signal.

Sample	Zero-time [ns]	Background Start [ns]	Background Cutoff [ns]	Modulation Depth (Δ)	Regularization Parameter (α)
75 µM 6R1/28H/32H + 15.0 µM Cu^{II}	263	4034	6206	0.038	10
75 µM 6R1/28H/32H + 30.0 µM Cu^{II}	275	2168	6192	0.085	10
75 µM 6R1/28H/32H + 37.5 µM Cu^{II}	278	1548	6192	0.100	10
75 µM 6R1/28H/32H + 45.0 µM Cu^{II}	280	4954	6192	0.070	10
75 µM 6R1/28H/32H + 60.0 µM Cu^{II}	277	4025	6192	0.104	10
75 µM 6R1/28H/32H + 75.0 µM Cu^{II}	274	1548	6192	0.053	10
75 µM 6R1/28H/32H + 112.5 µM Cu^{II}	271	1236	2752	0.017	1000
75 µM 6R1/28H/32H + 150.0 µM Cu^{II}	315	1296	6848	-	-

Table S5: Background correction parameters and modulation depths for the PELDOR data shown in figures S16-S23.

As seen from figures S8-15, all RIDME distance distributions give a significant distance peak (i.e., above the noise floor) at approximately 2.5 nm, which is highly consistent with the expected Cu^{II}-nitroxide inter-spin distance[1,7]. A low intensity feature at approximately 5.0 nm present at low metal:protein ratios (1:5 and 2:5) likely arises due to a combination of imperfect background correction and limiting signal-to-noise ratio (SNR), being below the noise floor for all higher metal:protein ratios. However, this feature may also arise from non-negligible longitudinal relaxation of the nitroxide electron spins during the mixing block interval, leading to contributions to the detected echo from both the Cu^{II}-nitroxide (~2.5 nm) and nitroxide-nitroxide (~5.0 nm) interspin distances. Furthermore, this hypothesis is also supported by comparison with the nitroxide-nitroxide PELDOR data, wherein the interspin nitroxide-nitroxide distance across the Cu^{II}-bridged dimer is ~5.0 nm.

As shown in figures S16-21, the PELDOR distance distributions consistently show a significant bimodal peak, with a maximum around 5.0 nm, which would correspond approximately to the predicted nitroxide-nitroxide interspin distance in the Cu^{II}-templated GB1 dimer (i.e., double the Cu^{II}-nitroxide inter-spin distance). Speculatively, the bimodality of the distribution peaks may suggest that the dimer complex is flexible, with monomers able to bend towards each other, or that monomers can align in a parallel or antiparallel fashion around the Cu^{II} centre. Alternatively, the bimodality could arise due to heterogeneous coordination geometries around the Cu^{II}-centre, perhaps deviating from perfect square-planar geometry.[12] To validate this hypothesis would require measurement of PELDOR traces with a longer time window, to ensure the peak is in the shape reliable (green colour bar) region. As shown in figures S22-23, at higher metal:protein ratios the PELDOR SNR is limiting, necessitating the use of a larger regularization parameter, or precluding analysis altogether, respectively. This modulation depth behaviour is indicative of negative cooperativity of Cu^{II}-templated dimerization in Tris-HCl buffer.

To confirm the trend in PELDOR modulation depths (i.e., maximum at 4:5 metal:protein ratio) in Tris-HCl buffer, spot-check samples (i.e., replicates of 3:5 and 4:5 metal:protein ratios) were prepared and PELDOR measurements repeated. Negative cooperativity of dimer formation should yield the maximum PELDOR modulation depth close to a metal:protein ratio of 1:2. Validated data is shown in figures S24-25, with background correction parameters and modulation depths given in

table S6. It is observed that the modulation depths reverse ordering between the original and replicate samples, with the 3:5 metal:protein ratio sample having slightly higher modulation depth than the 4:5 metal:protein ratio. This is more consistent with negative cooperativity (i.e., maximum closer to metal:protein ratio of 1:2) and indicates the negative cooperativity observed is reproducible behaviour in Tris-HCl buffer.

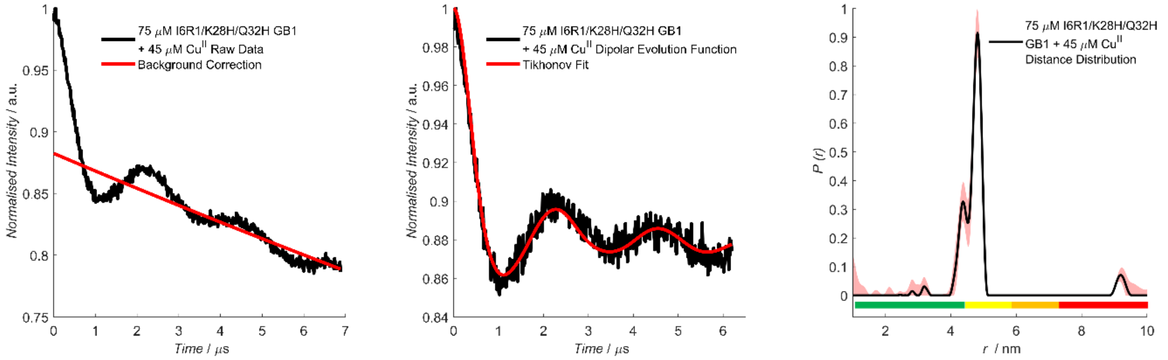

Figure S24. Validated Q-band nitroxide-nitroxide PELDOR data for the replicate spot check sample of construct I6R1/K28H/Q32H, in presence of Tris-HCl buffer, and Cu^{II} at a metal:protein ratio of 3:5. PELDOR raw time trace, background corrected trace, and validated distance distribution are shown left-to-right, respectively.

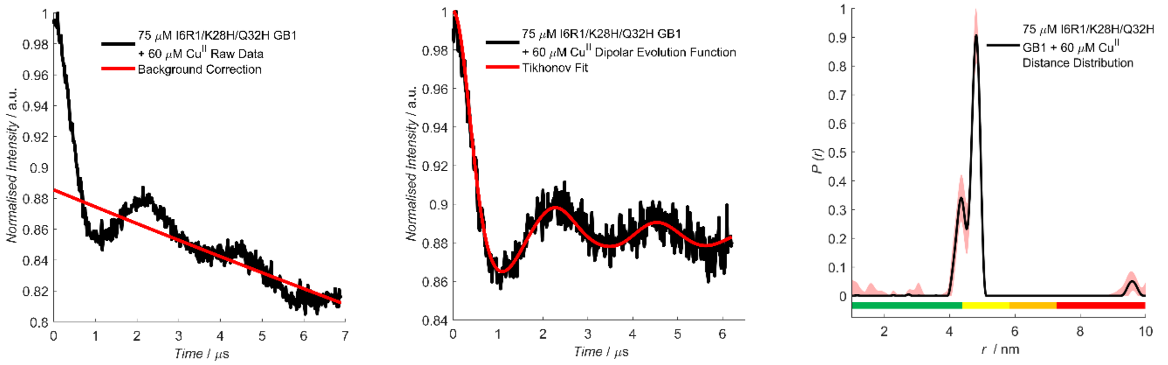

Figure S25. Validated Q-band nitroxide-nitroxide PELDOR data for the replicate spot check sample of construct I6R1/K28H/Q32H, in presence of Tris-HCl buffer, and Cu^{II} at a metal:protein ratio of 4:5. PELDOR raw time trace, background corrected trace, and validated distance distribution are shown left-to-right, respectively.

Sample	Zero-time [ns]	Background Start [ns]	Background Cutoff [ns]	Modulation Depth (Δ)	Regularization Parameter (α)
75 µM 6R1/28H/32H + 45.0 µM Cu^{II}	275	1548	6192	0.117	10
75 µM 6R1/28H/32H + 60.0 µM Cu^{II}	264	2482	6206	0.115	10

Table S6: Background correction parameters and modulation depths for the PELDOR data shown in figures S24-S25.

2.3.2 Phosphate Buffer Series:

A 9-point pseudo-titration series (metal:protein ratios 1:10, 1:5, 2:5, 1:2, 3:5, 4:5, 1:1, 3:2, and 2:1) was prepared in phosphate buffer (150 mM NaCl, 42.4 mM Na_2HPO_4 and 7.6 mM KH_2PO_4, pH 7.4) and Cu^{II}-nitroxide RIDME and nitroxide-nitroxide PELDOR measurements were performed. These RIDME measurements were performed using a critically coupled resonator mode. RIDME validations were performed as described in section 1.5. These PELDOR validations were performed in one-step over 800 trials; 50 white noise iterations, and 16 iterations of background start position (between 5-80% of total PELDOR trace length). Validated RIDME and PELDOR traces are shown in figures S26-S34, and S35-S43, respectively. Colour bars indicate regions of the distance distributions that are shape reliable (green), mean distance and width reliable (yellow), mean distance reliable (orange), and no quantification possible (red). The background correction parameters, and modulation depths are given in tables S7-S8, respectively.

Cu^{II}-nitroxide RIDME:

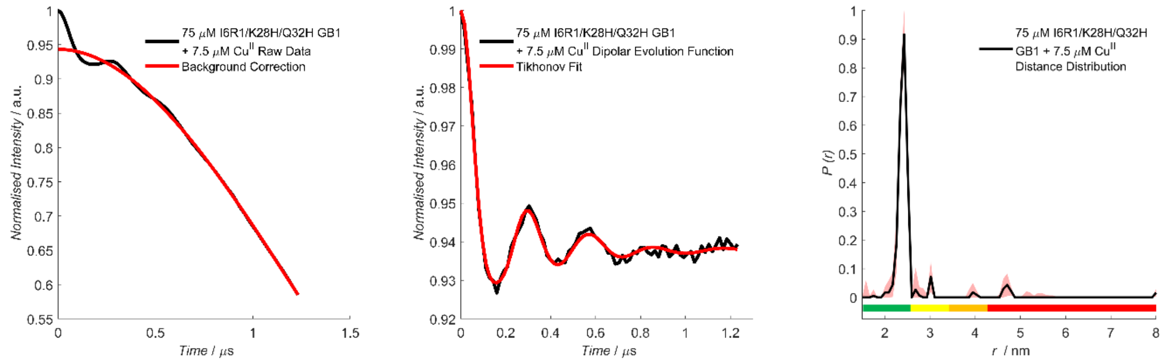

Figure S26. Validated Q-band Cu^{II}-nitroxide RIDME data for the construct I6R1/K28H/Q32H, in presence of phosphate buffer, and Cu^{II} at a metal:protein ratio of 1:10. RIDME raw time trace, background corrected trace, and validated distance distribution are shown left-to-right, respectively.

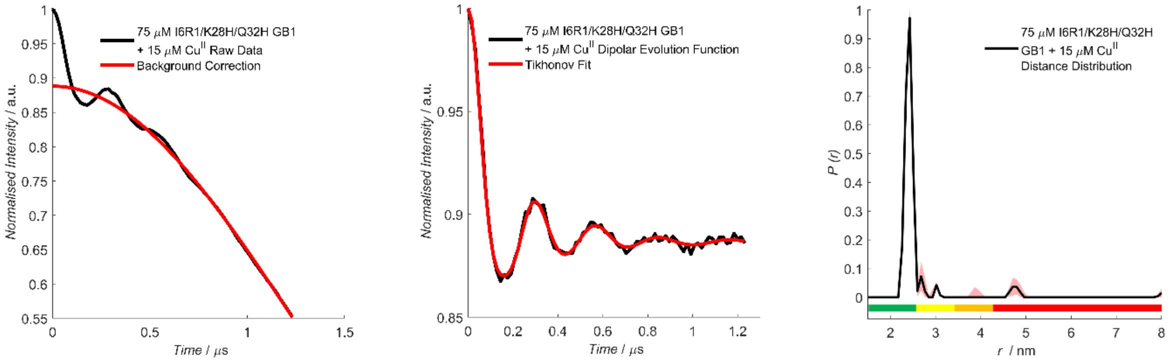

Figure S27. Validated Q-band Cu^{II}-nitroxide RIDME data for the construct I6R1/K28H/Q32H, in presence of phosphate buffer, and Cu^{II} at a metal:protein ratio of 1:5. RIDME raw time trace, background corrected trace, and validated distance distribution are shown left-to-right, respectively.

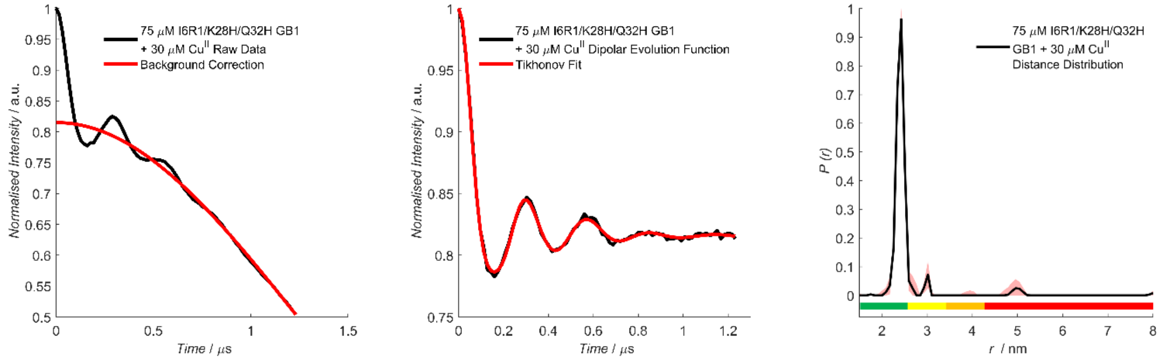

Figure S28. Validated Q-band Cu^{II}-nitroxide RIDME data for the construct I6R1/K28H/Q32H, in presence of phosphate buffer, and Cu^{II} at a metal:protein ratio of 2:5. RIDME raw time trace, background corrected trace, and validated distance distribution are shown left-to-right, respectively.

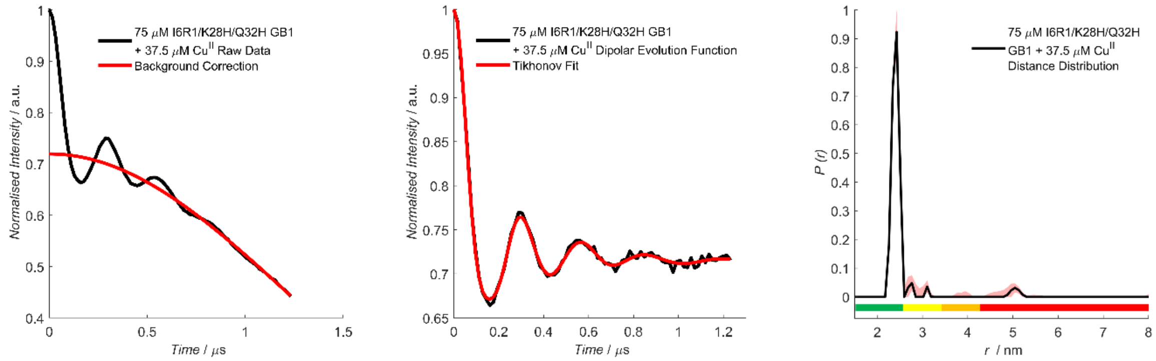

Figure S29. Validated Q-band Cu^{II}-nitroxide RIDME data for the construct I6R1/K28H/Q32H, in presence of phosphate buffer, and Cu^{II} at a metal:protein ratio of 1:2. RIDME raw time trace, background corrected trace, and validated distance distribution are shown left-to-right, respectively.

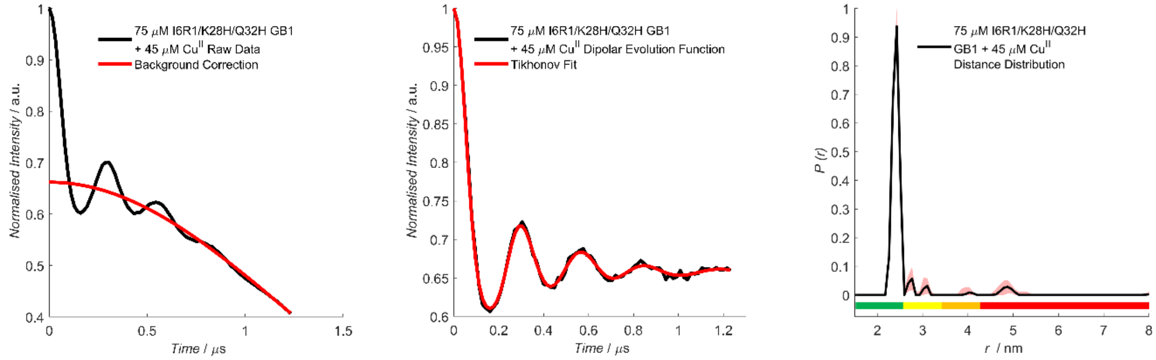

Figure S30. Validated Q-band Cu^{II}-nitroxide RIDME data for the construct I6R1/K28H/Q32H, in presence of phosphate buffer, and Cu^{II} at a metal:protein ratio of 3:5. RIDME raw time trace, background corrected trace, and validated distance distribution are shown left-to-right, respectively

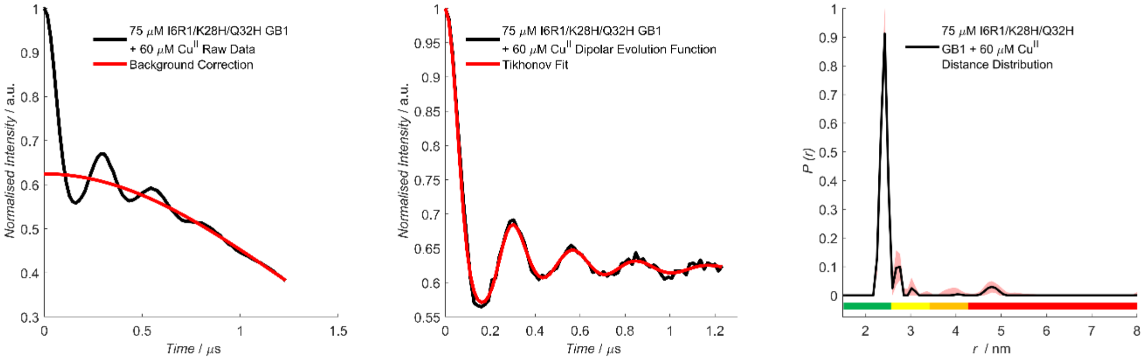

Figure S31. Validated Q-band Cu^{II}-nitroxide RIDME data for the construct I6R1/K28H/Q32H, in presence of phosphate buffer, and Cu^{II} at a metal:protein ratio of 4:5. RIDME raw time trace, background corrected trace, and validated distance distribution are shown left-to-right, respectively.

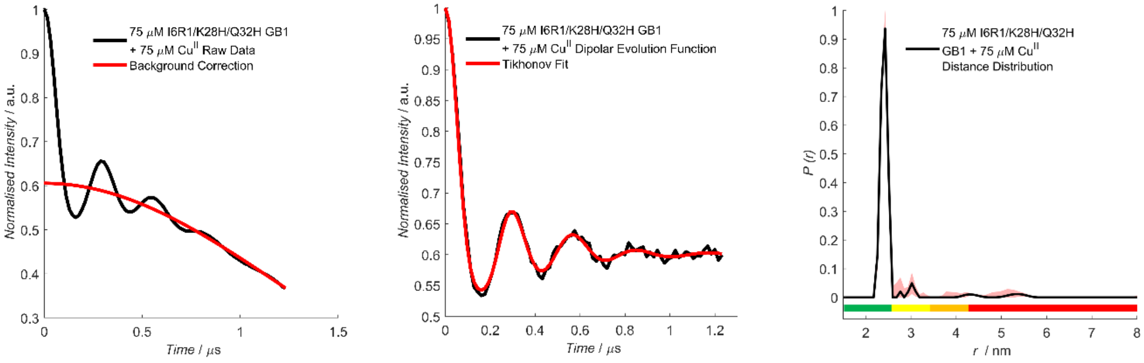

Figure S32. Validated Q-band Cu^{II}-nitroxide RIDME data for the construct I6R1/K28H/Q32H, in presence of phosphate buffer, and Cu^{II} at a metal:protein ratio of 1:1. RIDME raw time trace, background corrected trace, and validated distance distribution are shown left-to-right, respectively.

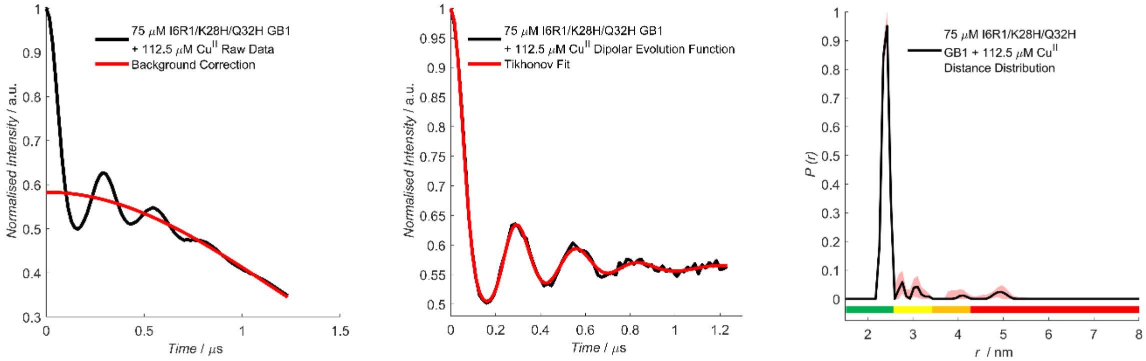

Figure S33. Validated Q-band Cu^{II}-nitroxide RIDME data for the construct I6R1/K28H/Q32H, in presence of phosphate buffer, and Cu^{II} at a metal:protein ratio of 3:2. RIDME raw time trace, background corrected trace, and validated distance distribution are shown left-to-right, respectively.

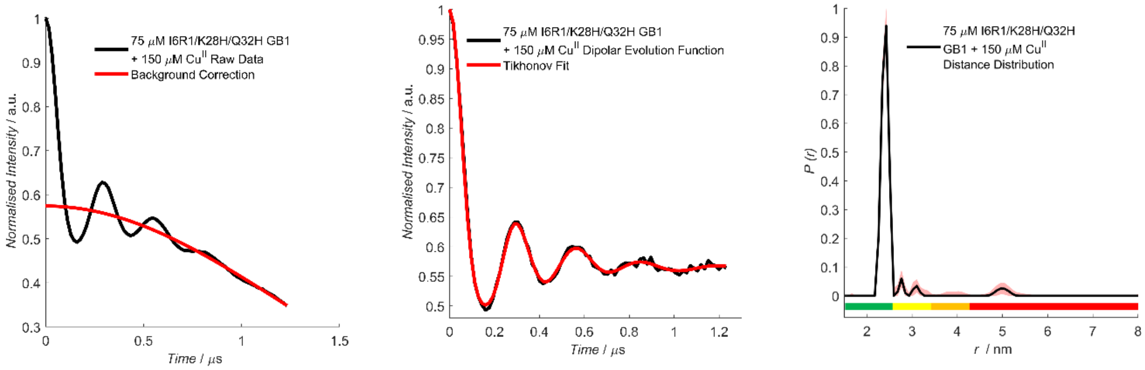

Figure S34. Validated Q-band Cu^{II}-nitroxide RIDME data for the construct I6R1/K28H/Q32H, in presence of phosphate buffer, and Cu^{II} at a metal:protein ratio of 2:1. RIDME raw time trace, background corrected trace, and validated distance distribution are shown left-to-right, respectively.

Sample	Zero-time [ns]	Background Start [ns]	Background Cutoff [ns]	Background Dimension	Modulation Depth (Δ)	Regularization Parameter (α)
75 µM 6R1/28H/32H + 7.5 µM Cu^{II}	210	370	1232	6	0.057	0.1
75 µM 6R1/28H/32H + 15.0 µM Cu^{II}	207	106	1232	6	0.112	0.1
75 µM 6R1/28H/32H + 30.0 µM Cu^{II}	210	62	1232	6	0.185	0.1
75 µM 6R1/28H/32H + 37.5 µM Cu^{II}	207	370	1232	6	0.281	0.1
75 µM 6R1/28H/32H + 45.0 µM Cu^{II}	206	194	1232	6	0.338	0.1
75 µM 6R1/28H/32H + 60.0 µM Cu^{II}	207	106	1232	6	0.375	0.1
75 µM 6R1/28H/32H + 75.0 µM Cu^{II}	206	370	1232	6	0.395	0.1
75 µM 6R1/28H/32H + 112.5 µM Cu^{II}	207	370	1232	6	0.417	0.1
75 µM 6R1/28H/32H + 150.0 µM Cu^{II}	206	150	1232	6	0.426	0.1

Table S7: Background correction parameters and modulation depths for the RIDME data shown in figures S26-S34.

Nitroxide-nitroxide PELDOR:

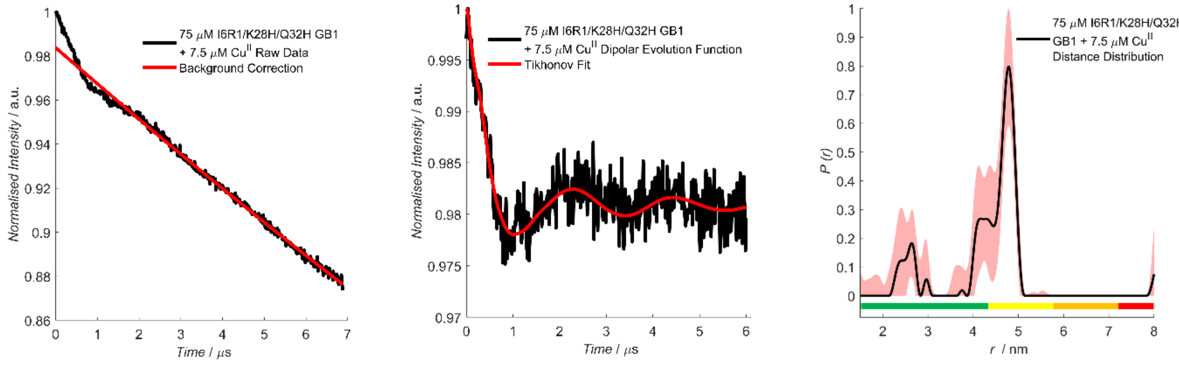

Figure S35. Validated Q-band nitroxide-nitroxide PELDOR data for the construct I6R1/K28H/Q32H, in presence of phosphate buffer, and Cu^{II} at a metal:protein ratio of 1:10. PELDOR raw time trace, background corrected trace, and validated distance distribution are shown left-to-right, respectively.

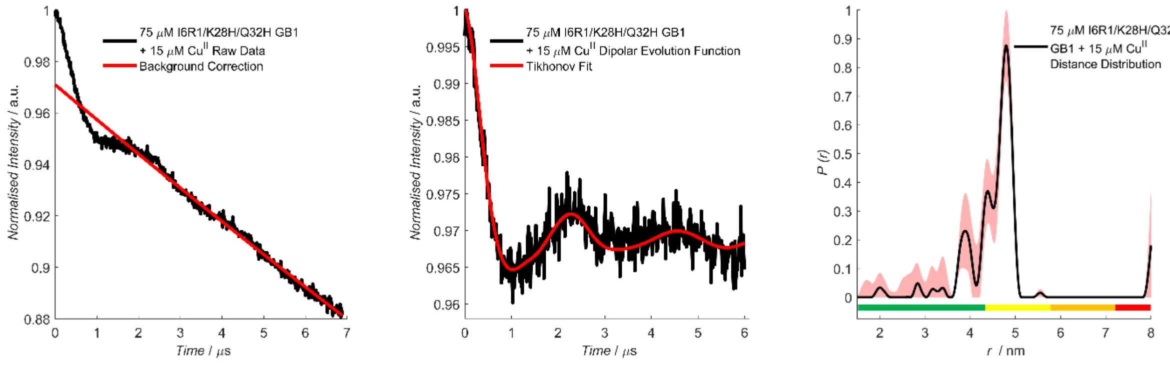

Figure S36. Validated Q-band nitroxide-nitroxide PELDOR data for the construct I6R1/K28H/Q32H, in presence of phosphate buffer, and Cu^{II} at a metal:protein ratio of 1:5. PELDOR raw time trace, background corrected trace, and validated distance distribution are shown left-to-right, respectively.

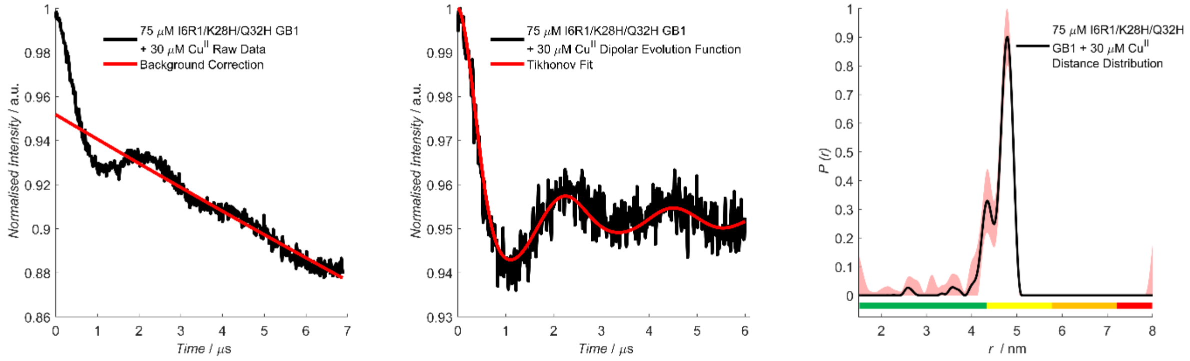

Figure S37. Validated Q-band nitroxide-nitroxide PELDOR data for the construct I6R1/K28H/Q32H, in presence of phosphate buffer, and Cu^{II} at a metal:protein ratio of 2:5. PELDOR raw time trace, background corrected trace, and validated distance distribution are shown left-to-right, respectively.

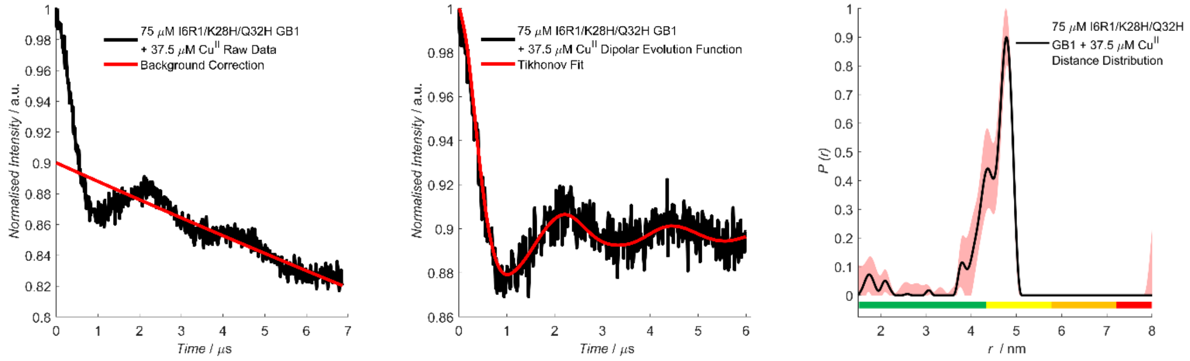

Figure S38. Validated Q-band nitroxide-nitroxide PELDOR data for the construct I6R1/K28H/Q32H, in presence of phosphate buffer, and Cu^{II} at a metal:protein ratio of 1:2. PELDOR raw time trace, background corrected trace, and validated distance distribution are shown left-to-right, respectively.

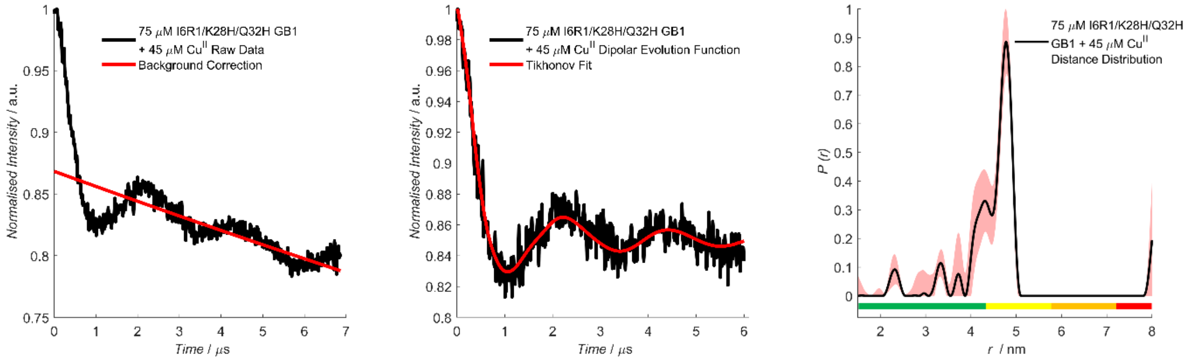

Figure S39. Validated Q-band nitroxide-nitroxide PELDOR data for the construct I6R1/K28H/Q32H, in presence of phosphate buffer, and Cu^{II} at a metal:protein ratio of 3:5. PELDOR raw time trace, background corrected trace, and validated distance distribution are shown left-to-right, respectively.

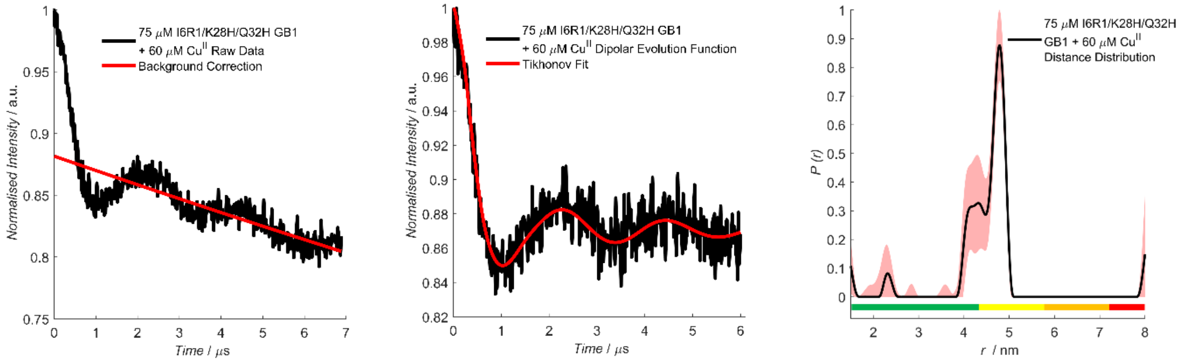

Figure S40. Validated Q-band nitroxide-nitroxide PELDOR data for the construct I6R1/K28H/Q32H, in presence of phosphate buffer, and Cu^{II} at a metal:protein ratio of 4:5. PELDOR raw time trace, background corrected trace, and validated distance distribution are shown left-to-right, respectively.

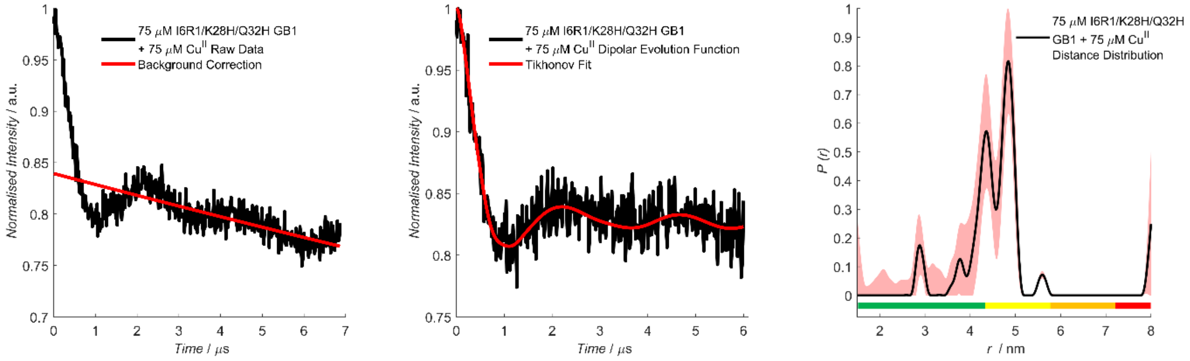

Figure S41. Validated Q-band nitroxide-nitroxide PELDOR data for the construct I6R1/K28H/Q32H, in presence of phosphate buffer, and Cu^{II} at a metal:protein ratio of 1:1. PELDOR raw time trace, background corrected trace, and validated distance distribution are shown left-to-right, respectively.

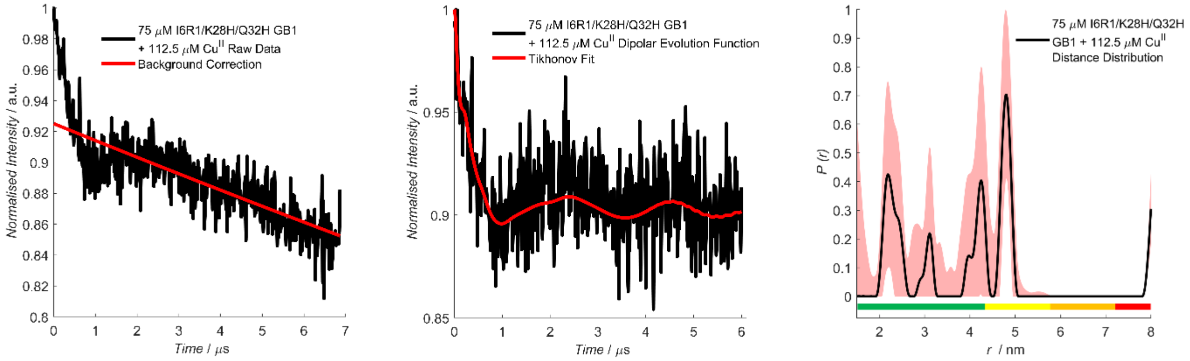

Figure S42. Validated Q-band nitroxide-nitroxide PELDOR data for the construct I6R1/K28H/Q32H, in presence of phosphate buffer, and Cu^{II} at a metal:protein ratio of 3:2. PELDOR raw time trace, background corrected trace, and validated distance distribution are shown left-to-right, respectively.

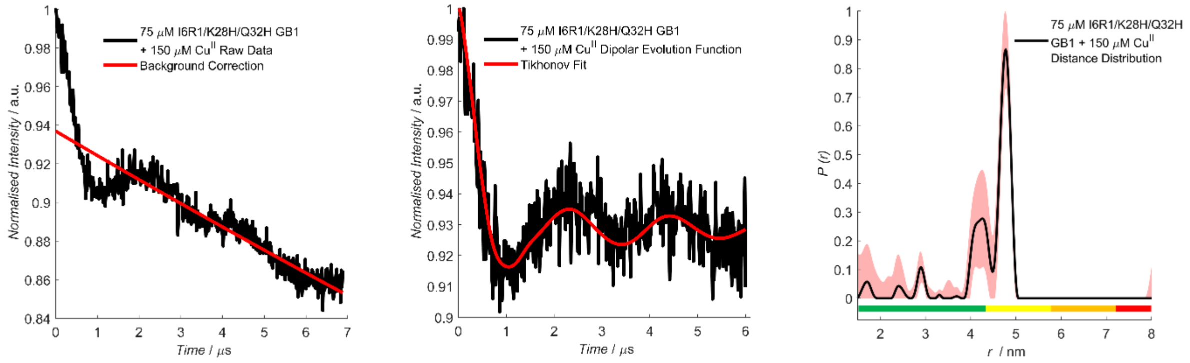

Figure S43. Validated Q-band nitroxide-nitroxide PELDOR data for the construct I6R1/K28H/Q32H, in presence of phosphate buffer, and Cu^{II} at a metal:protein ratio of 2:1. PELDOR raw time trace, background corrected trace, and validated distance distribution are shown left-to-right, respectively.

Sample	Zero-time [ns]	Background Start [ns]	Background Cutoff [ns]	Modulation Depth (Δ)	Regularization Parameter (α)
75 µM 6R1/28H/32H + 7.5 µM Cu^{II}	278	3900	6000	0.016	10
75 µM 6R1/28H/32H + 15.0 µM Cu^{II}	278	3900	6000	0.029	10
75 µM 6R1/28H/32H + 30.0 µM Cu^{II}	270	4800	6000	0.048	10
75 µM 6R1/28H/32H + 37.5 µM Cu^{II}	287	3600	6000	0.100	10
75 µM 6R1/28H/32H + 45.0 µM Cu^{II}	290	3600	6000	0.132	10
75 µM 6R1/28H/32H + 60.0 µM Cu^{II}	273	1500	6000	0.118	10
75 µM 6R1/28H/32H + 75.0 µM Cu^{II}	288	3300	6000	0.161	10
75 µM 6R1/28H/32H + 112.5 µM Cu^{II}	288	900	6000	0.075	10
75 µM 6R1/28H/32H + 150.0 µM Cu^{II}	277	4500	6000	0.063	10

Table S8: Background correction parameters and modulation depths for the PELDOR data shown in figures S35-S43.

Validated RIDME and PELDOR distance distributions are highly conserved between Tris-HCl and phosphate buffers, with a significant peak in the RIDME distributions of ~2.5 nm, and a significant peak in the PELDOR distributions of ~5.0 nm. However, further insight can be gained upon comparison of tables S4 and S7, which indicate that the Cu^{II}-nitroxide RIDME modulation depths are consistently higher at identical Cu^{II} concentrations in presence of Tris-HCl buffer, compared to phosphate buffer. This suggests that the affinity of Cu^{II} binding for double histidine (dH) motifs is higher in Tris-HCl buffer, and could rationalise the observed negative cooperativity for templated dimer formation (i.e., Cu^{II} binds tightly to the dH site and therefore more GB1 molecules will have Cu^{II}-bound dH sites which outcompetes the formation of dimer, wherein only one GB1 molecule has an occupied dH site). This is further supported by the declining trend observed in PELDOR

modulation depths at and above stoichiometric metal:protein ratios in Tris-HCl buffer, as confirmed by the replicate sample measurements.

Instead, comparison of table S5 and S8 indicates that in phosphate buffer the PELDOR modulation depth is maximised at stoichiometric metal:protein ratios and even at higher ratios, declines more softly than observed in Tris-HCl buffer. Given that precipitation of Cu^{II} by phosphate buffer will reduce the effective Cu^{II} concentration, the observed positive cooperativity of dimer formation in phosphate buffer can be rationalised (i.e., less Cu^{II} is available to coordinate the dH sites, resulting in less competition for the formation of templated dimer, wherein fewer GB1 molecules will have an occupied dH site, resulting in preferential binding to Cu^{II} already bound to a GB1 molecule and dimer formation).

To confirm the phosphate buffer series PELDOR modulation depths were reproducible and consistently indicated positive cooperativity of dimer formation, replicate measurements of the series were performed. The validated PELDOR traces are shown in figures S44-S52, with background correction parameters and modulation depths given in table S9. The observed modulation depth trend is highly consistent between the original and replicated measurement series.

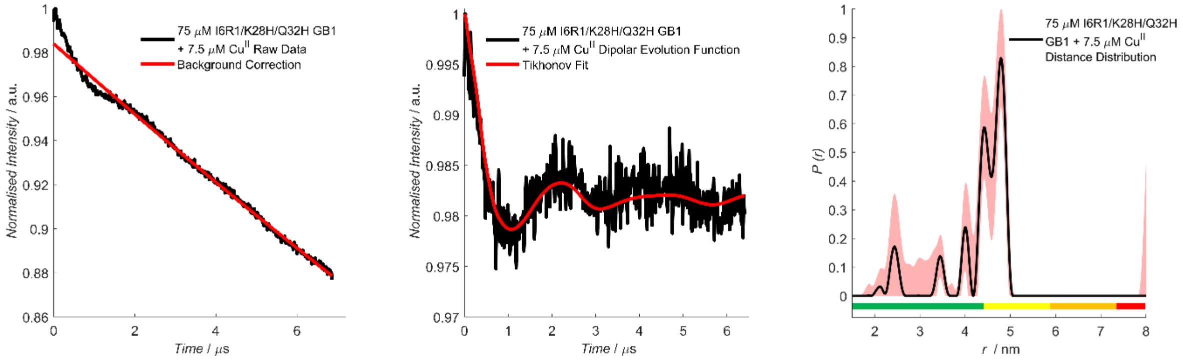

Figure S44. Validated repeat Q-band nitroxide-nitroxide PELDOR data for the construct I6R1/K28H/Q32H, in presence of phosphate buffer, and Cu^{II} at a metal:protein ratio of 1:10. PELDOR raw time trace, background corrected trace, and validated distance distribution are shown left-to-right, respectively.

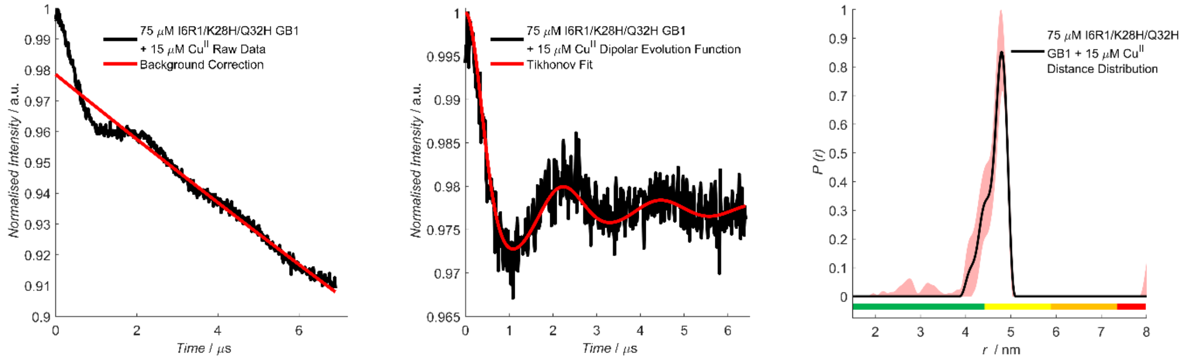

Figure S45. Validated repeat Q-band nitroxide-nitroxide PELDOR data for the construct I6R1/K28H/Q32H, in presence of phosphate buffer, and Cu^{II} at a metal:protein ratio of 1:5. PELDOR raw time trace, background corrected trace, and validated distance distribution are shown left-to-right, respectively.

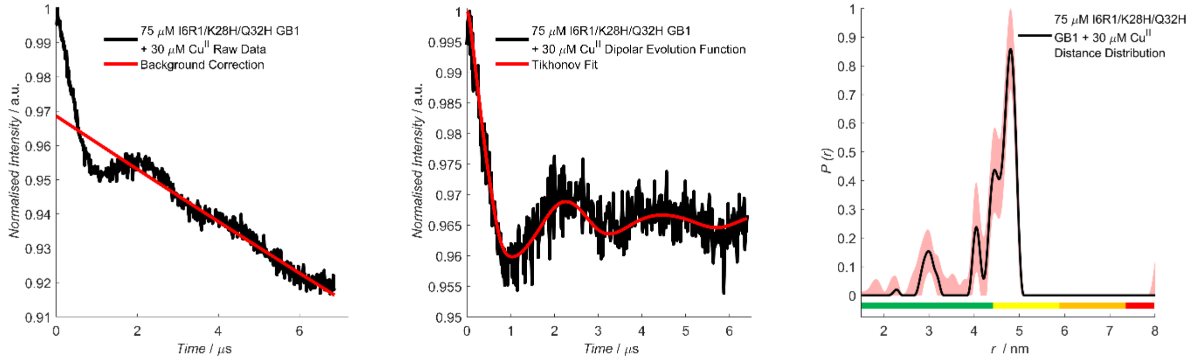

Figure S46. Validated repeat Q-band nitroxide-nitroxide PELDOR data for the construct I6R1/K28H/Q32H, in presence of phosphate buffer, and Cu^{II} at a metal:protein ratio of 2:5. PELDOR raw time trace, background corrected trace, and validated distance distribution are shown left-to-right, respectively.

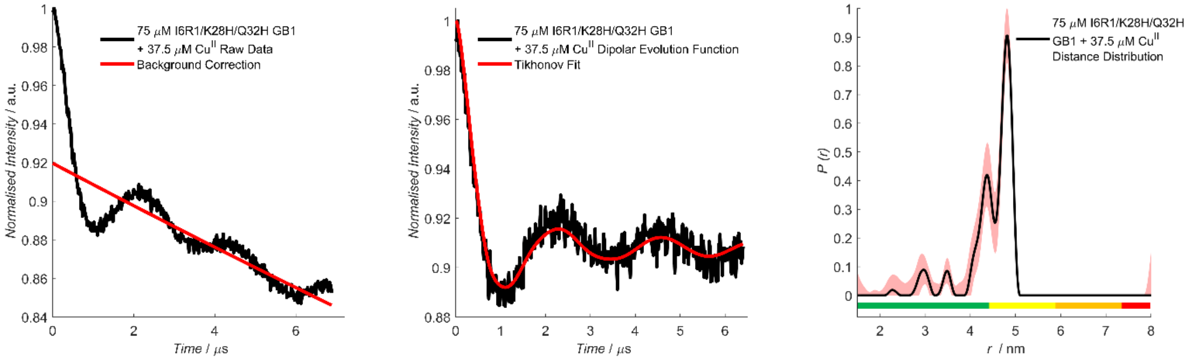

Figure S47. Validated repeat Q-band nitroxide-nitroxide PELDOR data for the construct I6R1/K28H/Q32H, in presence of phosphate buffer, and Cu^{II} at a metal:protein ratio of 1:2. PELDOR raw time trace, background corrected trace, and validated distance distribution are shown left-to-right, respectively.

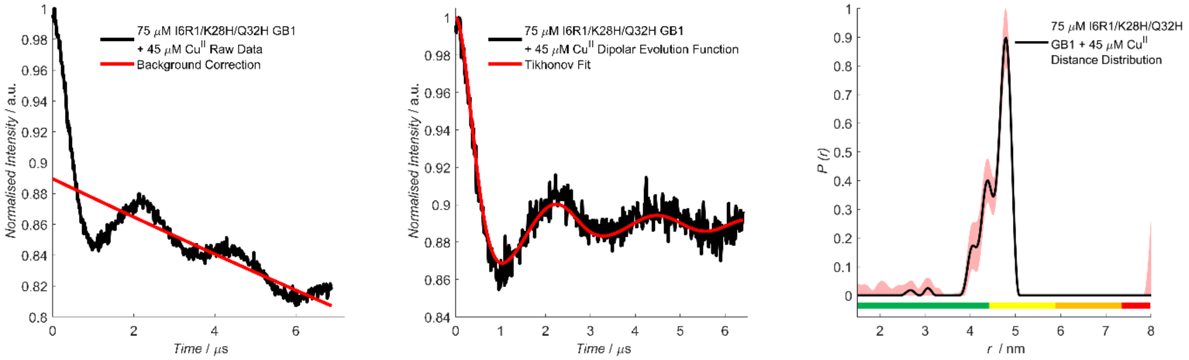

Figure S48. Validated repeat Q-band nitroxide-nitroxide PELDOR data for the construct I6R1/K28H/Q32H, in presence of phosphate buffer, and Cu^{II} at a metal:protein ratio of 3:5. PELDOR raw time trace, background corrected trace, and validated distance distribution are shown left-to-right, respectively.

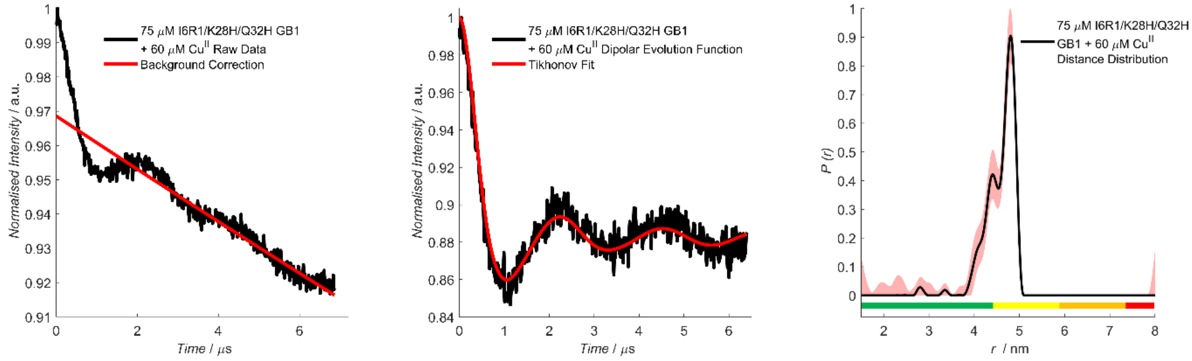

Figure S49. Validated repeat Q-band nitroxide-nitroxide PELDOR data for the construct I6R1/K28H/Q32H, in presence of phosphate buffer, and Cu^{II} at a metal:protein ratio of 4:5. PELDOR raw time trace, background corrected trace, and validated distance distribution are shown left-to-right, respectively.

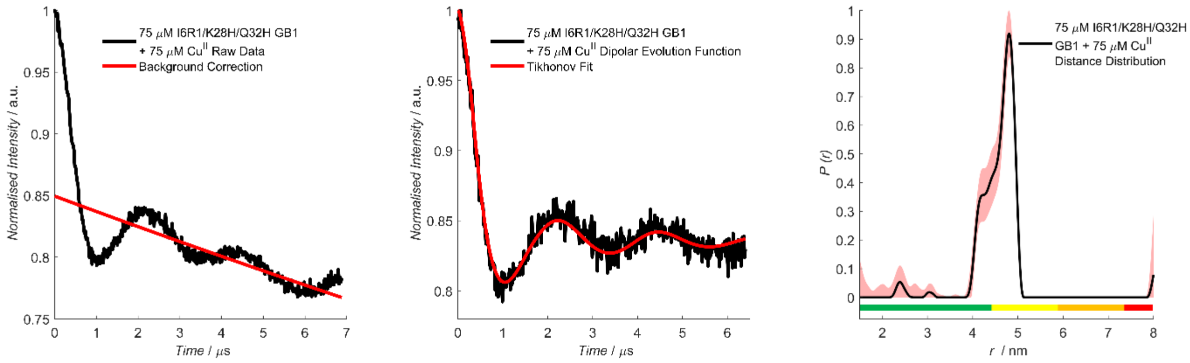

Figure S50. Validated repeat Q-band nitroxide-nitroxide PELDOR data for the construct I6R1/K28H/Q32H, in presence of phosphate buffer, and Cu^{II} at a metal:protein ratio of 1:1. PELDOR raw time trace, background corrected trace, and validated distance distribution are shown left-to-right, respectively.

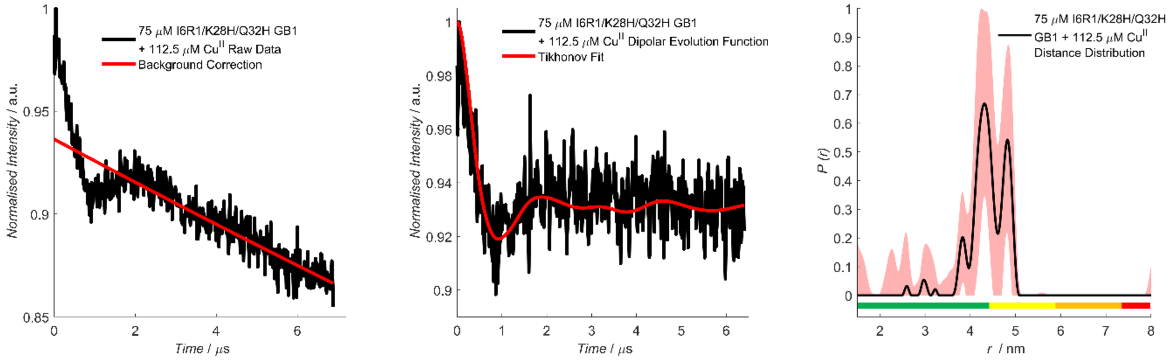

Figure S51. Validated repeat Q-band nitroxide-nitroxide PELDOR data for the construct I6R1/K28H/Q32H, in presence of phosphate buffer, and Cu^{II} at a metal:protein ratio of 3:2. PELDOR raw time trace, background corrected trace, and validated distance distribution are shown left-to-right, respectively.

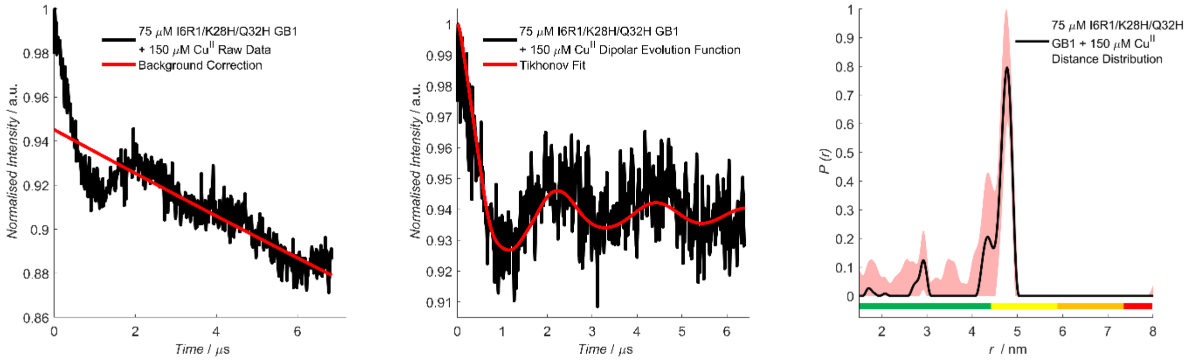

Figure S52. Validated repeat Q-band nitroxide-nitroxide PELDOR data for the construct I6R1/K28H/Q32H, in presence of phosphate buffer, and Cu^{II} at a metal:protein ratio of 2:1. PELDOR raw time trace, background corrected trace, and validated distance distribution are shown left-to-right, respectively.

Sample	Zero-time [ns]	Background Start [ns]	Background Cutoff [ns]	Modulation Depth (Δ)	Regularization Parameter (α)
75 µM 6R1/28H/32H + 7.5 µM Cu^{II}	300	4480	6400	0.016	10
75 µM 6R1/28H/32H + 15.0 µM Cu^{II}	254	5120	6400	0.021	10
75 µM 6R1/28H/32H + 30.0 µM Cu^{II}	293	4800	6400	0.031	10
75 µM 6R1/28H/32H + 37.5 µM Cu^{II}	280	4160	6400	0.080	10
75 µM 6R1/28H/32H + 45.0 µM Cu^{II}	275	3520	6400	0.107	10
75 µM 6R1/28H/32H + 60.0 µM Cu^{II}	273	4800	6400	0.110	10
75 µM 6R1/28H/32H + 75.0 µM Cu^{II}	269	2880	6400	0.151	10
75 µM 6R1/28H/32H + 112.5 µM Cu^{II}	275	3200	6400	0.064	10
75 µM 6R1/28H/32H + 150.0 µM Cu^{II}	303	3520	6400	0.055	10

Table S9: Background correction parameters and modulation depths for the PELDOR data shown in figures S44-S52.

2.4 Continuous Wave EPR Measurements:

Continuous-wave EPR measurements were performed to determine the suitability of buffers for use in this study. Concentration series of 0.1, 0.5, 1.0, 1.5, and 2.5 mM $CuCl_2$ were prepared in Good's buffers: Tris-HCl (20 mM Tris-HCl, 50 mM NaCl, pH 7.4), and MOPS (20 mM MOPS, 50 mM NaCl, pH 7.4), along with PBS (8.1 mM Na_2HPO_4, 1.5 mM KH_2PO_4, 2.7 mM KCl, 137 mM NaCl, pH 7.3), and compared against a concentration series prepared in H_2O. Results are shown in figures S53-S56, respectively, and indicate that only Tris-HCl does not cause complete precipitation of $CuCl_2$.

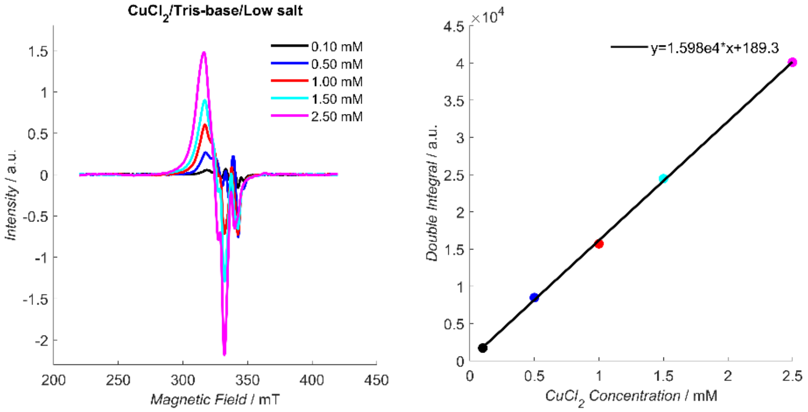

Figure S53. Continuous-wave EPR data for the $CuCl_2$ concentration series (0.1, 0.5, 1.0, 1.5, 2.5 mM $CuCl_2$) in presence of Tris-HCl buffer (20 mM Tris, 50 mM NaCl, pH 7.4). The baseline corrected spectra and corresponding linear fit of the double integrals are shown in left and right panels, respectively.

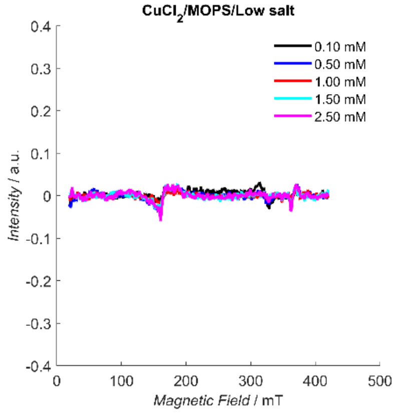

Figure S54. Continuous-wave EPR data for the $CuCl_2$ concentration series (0.1, 0.5, 1.0, 1.5, 2.5 mM $CuCl_2$) in presence of MOPS buffer (20 mM MOPS, 50 mM NaCl, pH 7.4).

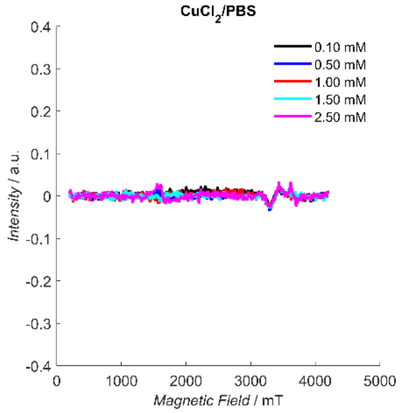

Figure S55. Continuous-wave EPR data for the $CuCl_2$ concentration series (0.1, 0.5, 1.0, 1.5, 2.5 mM $CuCl_2$) in presence of PBS buffer (8.1 mM Na_2HPO_4, 1.5 mM KH_2PO_4, 2.7 mM KCl, 137 mM NaCl, pH 7.3).

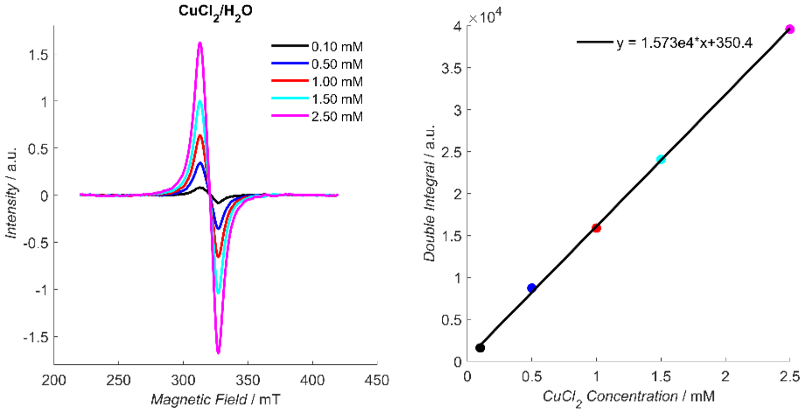

Figure S56. Continuous-wave EPR data for the $CuCl_2$ concentration series (0.1, 0.5, 1.0, 1.5, 2.5 mM $CuCl_2$) in presence of H_2O. The baseline corrected spectra and corresponding linear fit of the double integrals are shown in left and right panels, respectively.

Following this observation, Tris-HCl buffer was chosen for this study: Tris-HCl interacts strongly with Cu^{II} at pH 7.4[13] and therefore likely retains Cu^{II} in solution through chelation, even at alkaline pH. Further, this is supported by the line-shape change in presence of Tris-HCl buffer and indicates nitrogen coordination of the Cu^{II} centre. Indeed, the observation of $CuCl_2$ precipitating in presence of PBS and MOPS is not unexpected, since phosphate is known to strongly interact with Cu^{II}, forming insoluble Cu^{II} phosphate,[14] and MOPS buffer only weakly interacts with Cu^{II}.[15] However, additional $CuCl_2$ concentration series for Tris-HCl and PBS buffers, in presence of two molar equivalents of imidazole (as a mimic of double-histidine motifs present in proteins) indicated partial recovery (~5-15%) of soluble Cu^{II} for PBS buffer. This is likely because imidazole chelates Cu^{II} more strongly than phosphate, and so retains Cu^{II} in solution. Results are shown in figures S57-58, respectively, and comparison of the double integral values from all series are given in table S10.

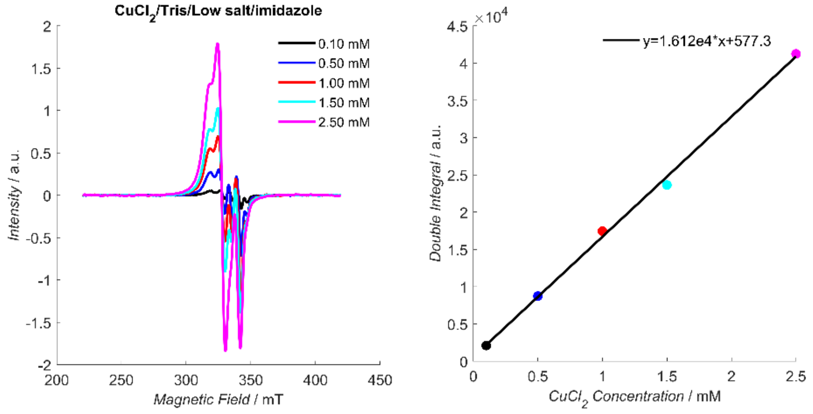

Figure S57. Continuous-wave EPR data for the $CuCl_2$ concentration series (0.1, 0.5, 1.0, 1.5, 2.5 mM $CuCl_2$) in presence of Tris-HCl buffer (20 mM Tris, 50 mM NaCl, pH 7.4) and two molar equivalents of imidazole. The baseline corrected spectra and corresponding linear fit of the double integrals are shown in left and right panels, respectively.

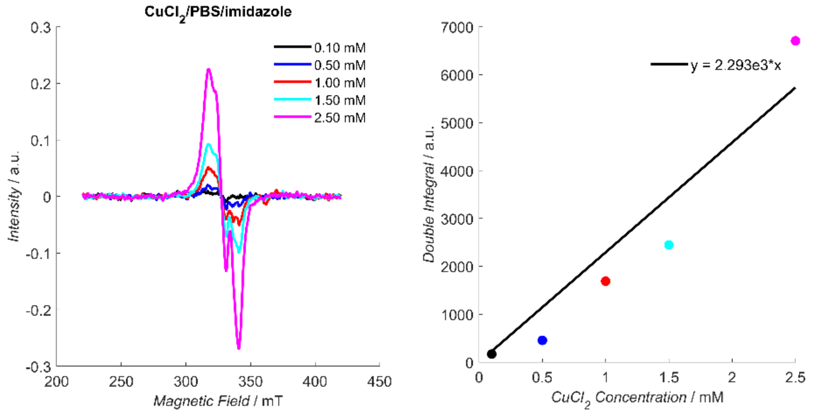

Figure S58. Continuous-wave EPR data for the $CuCl_2$ concentration series (0.1, 0.5, 1.0, 1.5, 2.5 mM $CuCl_2$) in presence of PBS buffer (8.1 mM Na_2HPO_4, 1.5 mM KH_2PO_4, 2.7 mM KCl, 137 mM NaCl, pH 7.3) and two molar equivalents of imidazole. The baseline corrected spectra and corresponding linear fit of the double integrals are shown in left and right panels, respectively.

$CuCl_2$ Concentration / mM	H_2O (relative DI / %)		PBS (relative DI / %)		Tris-HCl (relative DI / %)		MOPS (relative DI / %)
		imidazole		imidazole		imidazole	
0.1	100	71	0	11	108*	138*	0
0.5	100	83	0	5	97	100	0
1.0	100	83	0	11	99	110*	0
1.5	100	85	0	10	102*	98	0
2.5	100	90	0	17	101*	106*	0

Table S10: Relative double integral values of $CuCl_2$ concentration series performed in different buffers and in presence or absence of imidazole, compared with a $CuCl_2$ concentration series in presence of H_2O. *The relative double integrals higher than 100% arise from the imperfect baseline correction of the single integral.

Indeed, this finding of partial recovery of solubility was also reproduced with a $CuCl_2$ concentration series (0.05, 0.10, 0.15, 0.20, 0.30, 0.40 mM) measured in presence of PBS buffer and half a molar equivalent of K28H/Q32H GB1 protein, shown in figure S59. Comparison of relative double integral values with the $CuCl_2$ concentration series in presence of H_2O, given in table S11, indicate that recovery is improved in presence of protein (~60-100%) compared to free imidazole (~5-15%), and the observation of ~60% signal recovery at higher $CuCl_2$ concentrations is consistent with the optimal scaling factor of Cu^{II} concentration (0.65) for the phosphate buffer RIDME and PELDOR measurement series (section 2.5.3).

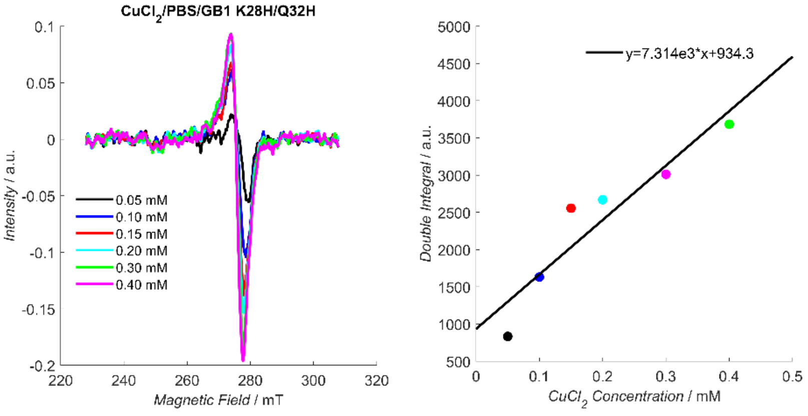

Figure S59. Continuous-wave EPR data for the $CuCl_2$ concentration series (0.05, 0.10, 0.15, 0.20, 0.30, 0.40 mM $CuCl_2$) in presence of PBS buffer (8.1 mM Na_2HPO_4, 1.5 mM KH_2PO_4, 2.7 mM KCl, 137 mM NaCl, pH 7.3) and half a molar equivalent of K28H/Q32H GB1 protein. The baseline corrected spectra and corresponding linear fit of the double integrals are shown in left and right panels, respectively.

$CuCl_2$ Concentration / mM	PBS (relative DI / %)
	GB1 protein
0.05	104*
0.10	101*
0.15	107*
0.20	84
0.30	63
0.40	58

Table S11: Relative double integral values of $CuCl_2$ concentration series performed in presence of PBS buffer and half a molar equivalent of K28H/Q32H GB1 protein, compared with a $CuCl_2$ concentration series in presence of H_2O. *The relative double integrals higher than 100% arise from the imperfect baseline correction of the single integral.

Interestingly, the partial recovery of solubility in presence of PBS buffer and dH GB1 protein suggests that the Cu^{II} binding equilibrium can be modulated by changing the buffer conditions. The hypothesis is that in Tris-HCl buffer, the majority of Cu^{II} remains soluble, and so the apparent dissociation constant (K_D) is lower (i.e., tighter binding) for the dH motif, which subsequently outcompetes the formation of Cu^{II}-templated dimer (i.e., one GB1 has Cu^{II} bound at the dH motif, and one GB1 has an unoccupied dH motif). Instead, in PBS buffer, the effective Cu^{II} concentration reduces (i.e., a non-negligible amount of Cu^{II} is precipitated) and therefore the apparent K_D increases for the dH motif. Subsequently, fewer GB1 molecules will have Cu^{II} bound at the dH motif, and therefore enhances the propensity for Cu^{II}-templated dimer formation. This should manifest as a strongly negative cooperativity of Cu^{II}-templated dimer formation in Tris-HCl buffer, and as a positive cooperativity of Cu^{II}-templated dimer formation in PBS buffer.

2.5 Estimating K_D and Cooperativity (α) Parameters:

2.5.1 Exploratory Simulations:

Exploratory simulations were performed to determine the robustness of the cooperative binding model to extract apparent K_D and cooperativity parameters from Cu^{II}-nitroxide RIDME and nitroxide-nitroxide PELDOR modulation depths. First, simulations were performed using a fixed cooperativity and changing the K_D value, to observe how the curve shapes changed with respect to the RIDME (i.e., loading of Cu^{II} at the dH site) and PELDOR (i.e., formation of the templated dimer) modulation depths, shown in the left and right panel of figure S60, respectively.

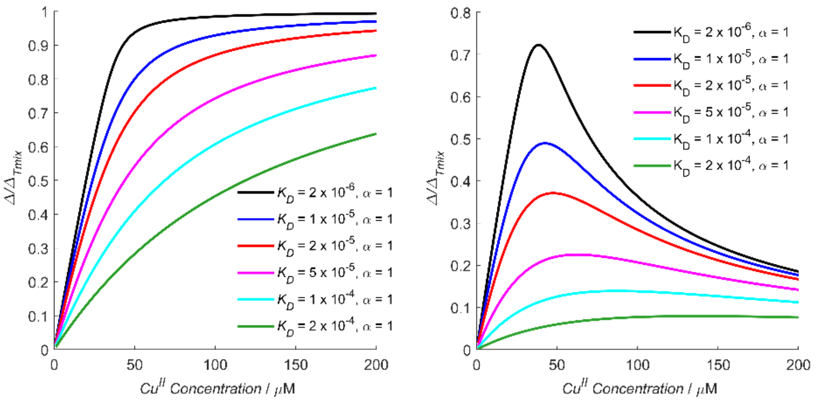

Figure S60. Simulated binding isotherms for RIDME (left) and PELDOR (right) modulation depths, assuming a fixed protein concentration and cooperativity parameter of 75 µM and 1.0, respectively, with varying K_D values and Cu^{II} concentration incremented from 1-200 µM in steps of 0.1 µM.

It is observed from figure S60 that the hyperbolic RIDME modulation depth curve rises more steeply and plateaus at lower concentrations of Cu^{II} for tighter binding affinity (i.e., lower K_D values), resulting in loss of resolution of curvature and approximating a step-function, consistent with expectation. While for the PELDOR modulation depth curve, tighter binding affinity both increases the amplitude of the global maximum towards unity and shifts the maximum to lower concentrations of Cu^{II}. Lower binding affinity results in a broadening of the global maximum and a shallower decay as Cu^{II} concentration is increased. This is consistent with previous literature detailing a non-cooperative (i.e., $\alpha = 1$) two-site binding model.[7]

Next, the influence of the cooperativity parameter on the shape of the binding isotherms was investigated, assuming a fixed K_D value of 2×10^{-5}, with the corresponding curves shown in figure S61. Once again, the RIDME modulation depth curve rises more steeply and plateaus at lower Cu^{II} concentrations for higher cooperativity factors. For the PELDOR modulation depth curve only the amplitude of the global maximum is affected by changing the cooperativity factor, with a slight broadening of the maximum at very negative cooperativity values, highly consistent with the original observations by Mack *et al*.[8]

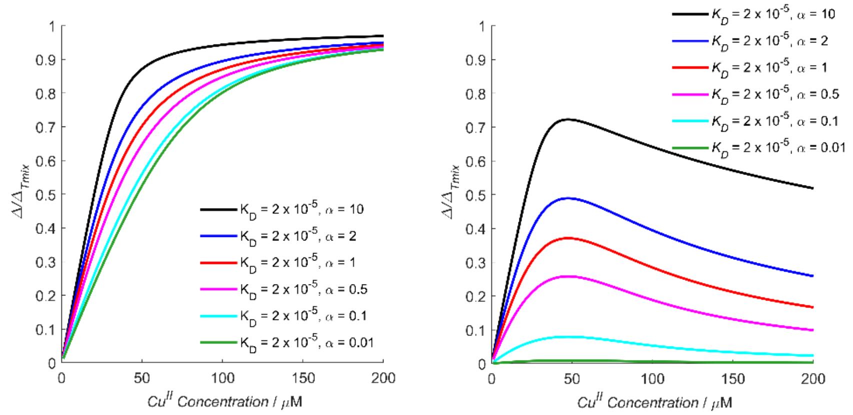

Figure S61. Simulated binding isotherms for RIDME (left) and PELDOR (right) modulation depths, assuming a fixed protein concentration and K_D of 75 μM and 2 $\times$ 10^{-5}, respectively, with varying cooperativity values and Cu^{II} concentration incremented from 1-200 μM in steps of 0.1 μM.

Finally, it was necessary to determine whether the binding model could successfully distinguish between i) high binding affinity and negative cooperativity, and ii) low binding affinity and positive cooperativity, even when the product of K_D and α^{-1} is constant. The expectation is that this disparity will influence the shape of the isotherms in different ways, and the resulting curves are shown in figure S62. It is observed that for case i), the behaviour of the RIDME modulation depth profile is consistent with overall lower K_D, with a steeper initial flank and earlier plateau, while the PELDOR modulation depth curve shows a steeper decay at lower K_D and negative cooperativity. For case ii), the behaviour of the RIDME modulation depth curve is distinct, with an initial steep rise before a slower rise and gradual plateau, while the PELDOR modulation depth curve shows a slower rise and subsequent decay for higher K_D and positive cooperativity.

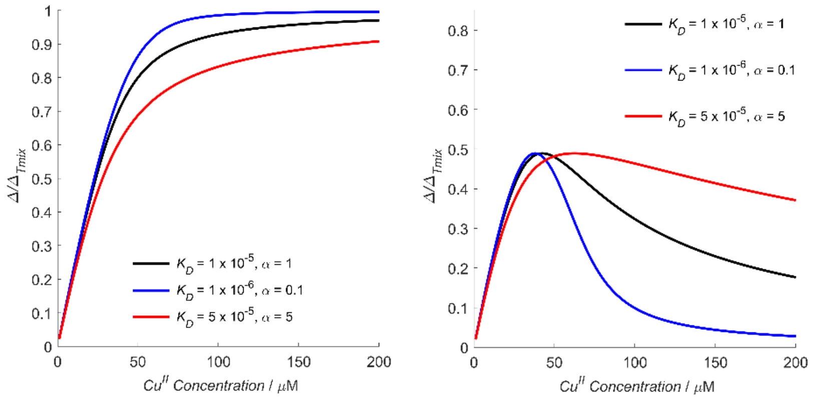

Figure S62. Simulated binding isotherms for RIDME (left) and PELDOR (right) modulation depths, assuming a fixed product $K_D \times \alpha^{-1}$ of 1×10^{-5}, with protein concentration of 75 μM and Cu^{II} concentration incremented from 1-200 μM in steps of 0.1 μM.

2.5.2 Reproducibility of Phosphate Buffer Series:

To check the reproducibility of the fitted α and K_D parameters, PELDOR measurements of the phosphate buffer series were repeated by averaging overnight to improve signal-to-noise ratio (SNR) before reprocessing. The corresponding fit and error contour are shown in figure S63. Both fitted parameters are consistent with the original measurement series, showing positive cooperativity and reduced binding affinity compared to the Tris-HCl buffer series (see figure 3 main text).

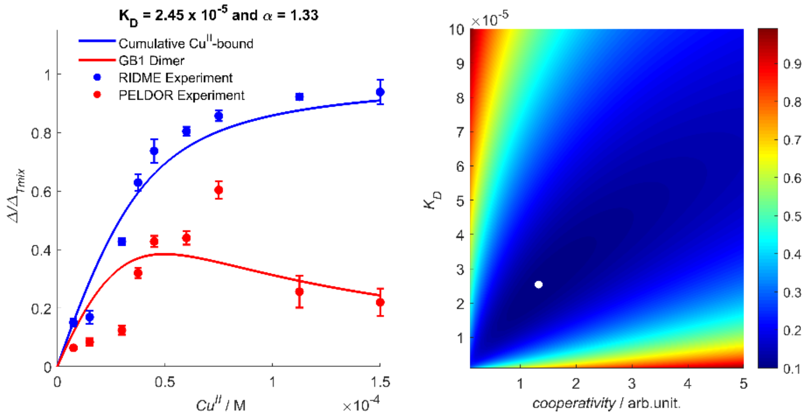

Figure S63. Fitted PELDOR and RIDME modulation depths as a function of Cu^{II} concentration (red and blue lines, respectively) from repeated phosphate buffer series PELDOR measurements, with experimental PELDOR and RIDME modulation depths (red and blue dots, respectively) in presence of phosphate buffer (left). Corresponding error contour, with the fitted parameters indicated by a white dot (right).

2.5.3 Scaled Fits:

To determine the robustness of fitted parameters to scaling of the experimental Cu^{II} concentrations, and the utility of scaling to improve the overall fits, datasets were scaled by factors 1.0 (unscaled), 0.95, 0.90, 0.85, 0.80, and 0.75 for the Tris buffer series, as well as 0.70, 0.65, 0.60, 0.55, 0.50, and 0.45 for the phosphate buffer series. Scaled fits of the Tris buffer series, original phosphate buffer series, and repeat phosphate buffer series datasets are shown in figures S64-68, with the K_D, cooperativity factor, and scaling factor indicated in the plot titles. The corresponding RMSD values are tabulated in tables S12-S14.

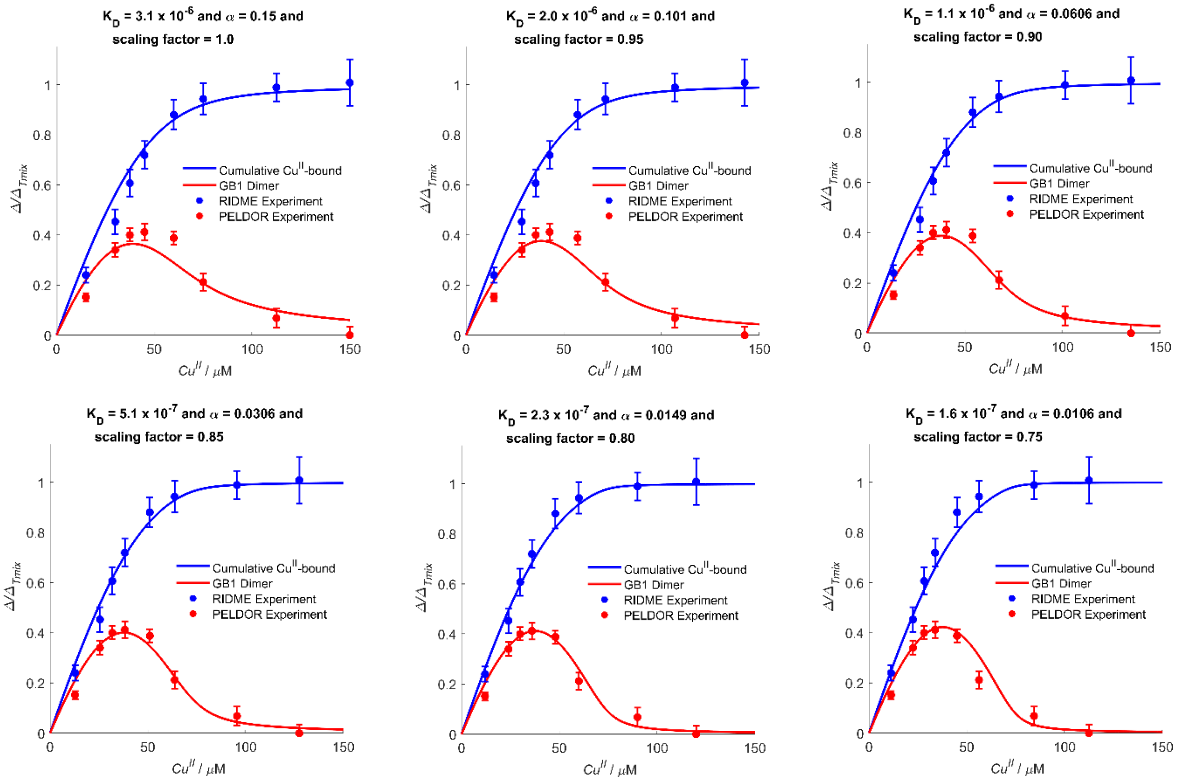

Figure S64: Scaled fits of the Tris buffer series dataset, scaled by factors 1.0, 0.95, 0.90 (top row, left-to-right) and 0.85, 0.80, 0.75 (bottom row, left-to-right).

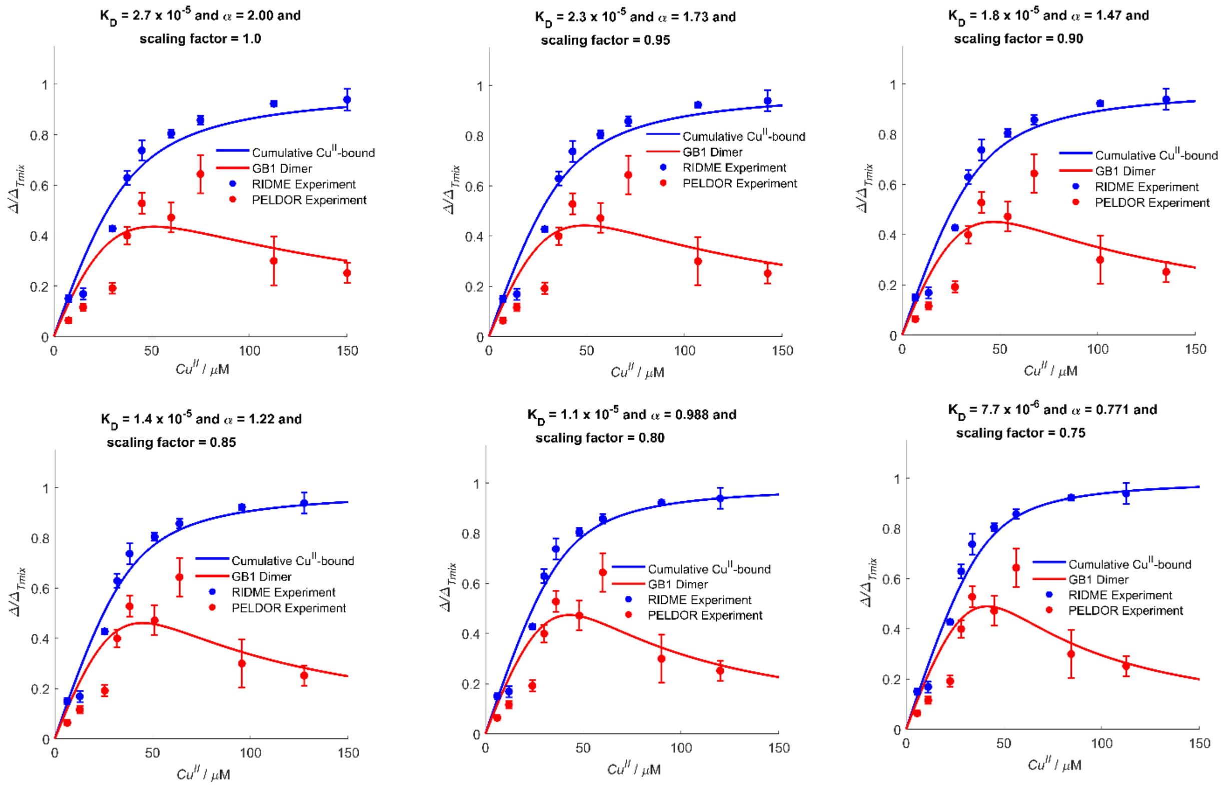

Figure S65. Scaled fits of the original phosphate buffer series dataset, scaled by factors 1.0, 0.95, 0.90 (top row, left-to-right) and 0.85, 0.80, 0.75 (bottom row, left-to-right).

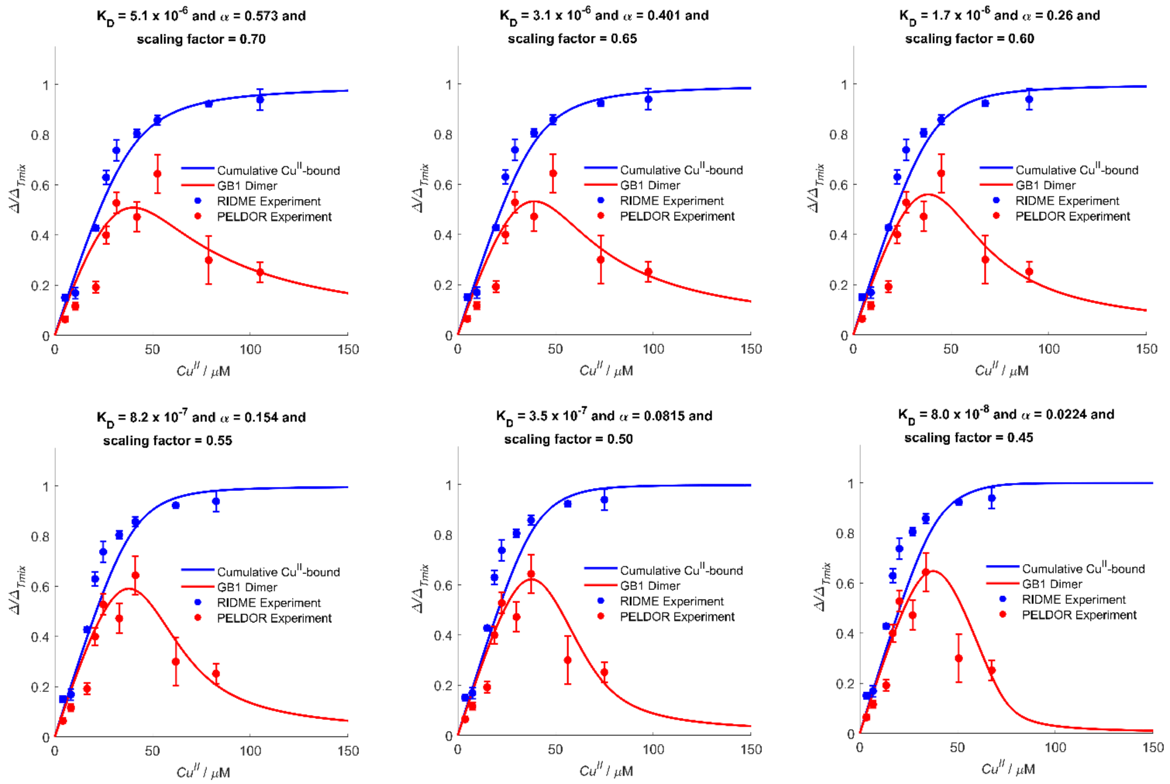

Figure S66. Scaled fits of the original phosphate buffer series dataset, scaled by factors 0.70, 0.65, 0.60 (top row, left-to-right) and 0.55, 0.50, 0.45 (bottom row, left-to-right).

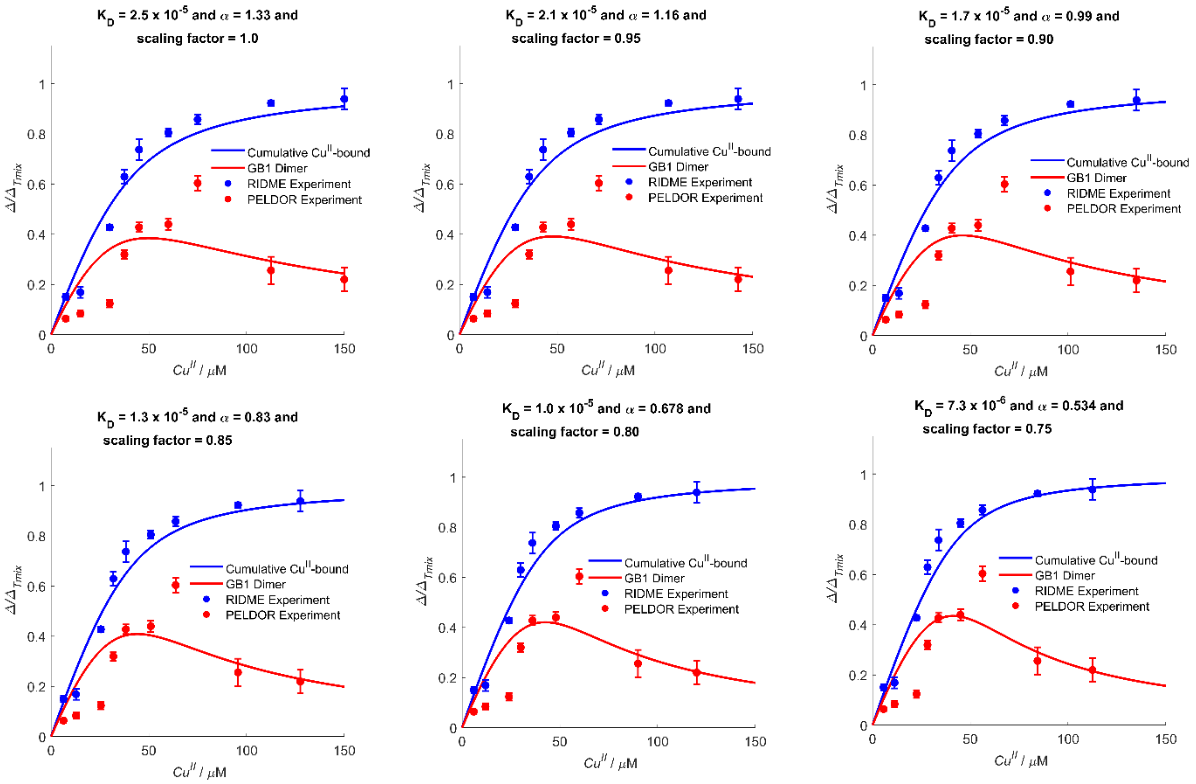

Figure S67: Scaled fits of the repeat phosphate buffer series dataset, scaled by factors 1.0, 0.95, 0.90 (top row, left-to-right) and 0.8, 0.80, 0.75 (bottom row, left-to-right).

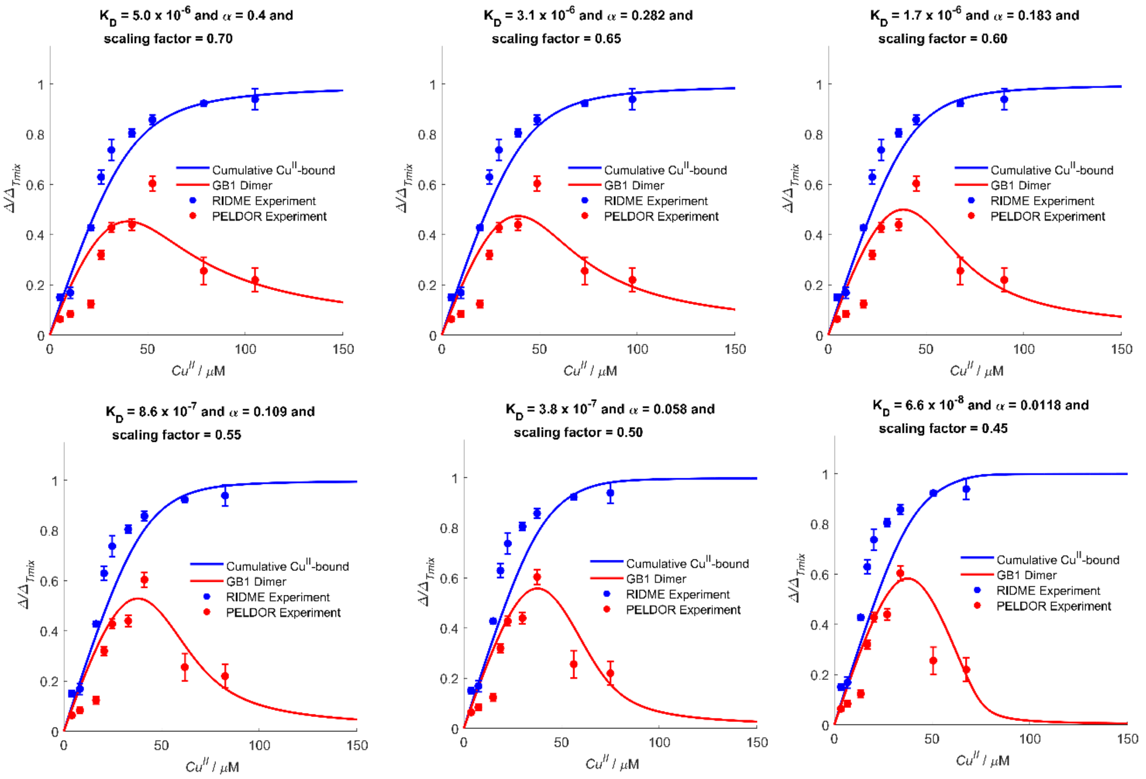

Figure S68: Scaled fits of the repeat phosphate buffer series dataset, scaled by factors 0.70, 0.65, 0.60 (top row, left-to-right) and 0.55, 0.50, 0.45 (bottom row, left-to-right).

Scaling Factor	RMSD / a.u.
1.00	2.28×10^{-2}
0.95	1.62×10^{-2}
0.90	1.05×10^{-2}
0.85	6.80×10^{-3}
0.80	7.10×10^{-3}
0.75	1.34×10^{-2}

Table S12: Global minimum RMSD values of Tris buffer measurement series in dependence of different scaling factors, shown in figure S64.

Scaling Factor	RMSD / a.u.
1.00	8.32×10^{-2}
0.95	7.81×10^{-2}
0.90	7.27×10^{-2}
0.85	6.71×10^{-2}
0.80	6.16×10^{-2}
0.75	5.64×10^{-2}
0.70	5.22×10^{-2}
0.65	4.98×10^{-2}
0.60	5.11×10^{-2}
0.55	5.91×10^{-2}
0.50	7.91×10^{-2}
0.45	1.19×10^{-1}

Table S13: Global minimum RMSD values of the original phosphate buffer measurement series in dependence of different scaling factors, shown in figures S65 and S66.

Scaling Factor	RMSD / a.u.
1.00	8.87×10^{-2}
0.95	8.47×10^{-2}
0.90	8.06×10^{-2}
0.85	7.66×10^{-2}
0.80	7.28×10^{-2}
0.75	6.95×10^{-2}
0.70	6.73×10^{-2}
0.65	6.70×10^{-2}
0.60	7.03×10^{-2}
0.55	7.99×10^{-2}
0.50	1.01×10^{-1}
0.45	1.39×10^{-1}

Table S14: Global minimum RMSD values of the repeat phosphate buffer measurement series in dependence of different scaling factors, shown in figure S67 and S68.

As seen from tables S12-S14, the scaling of the experimental Cu^{II} concentrations does improve the quality of fit of all series, compared to the unscaled dataset. The Tris buffer series dataset shows a global minimum at the scaling factor 0.85, while both phosphate buffer series datasets show a global minimum at the scaling factor 0.65. This would be consistent with the fitted maximum being at ~0.5-

0.6 equivalents, and furthermore indicates that a larger percentage of the Cu^{II} precipitates from solution for the phosphate buffer series, compared to the Tris buffer series, driving the observed positive cooperativity. For the scaling factor of 0.65, it can be seen from figures S64-68 that the poor fit of PELDOR modulation depths biases the Cu^{II} concentration to lower values to the extent that the hyperbolic fit of the RIDME modulation depths is significantly compromised. While the precipitation of Cu^{II} from solution has the desired effect of increasing cooperativity of templated dimer formation, it clearly makes the fitting more volatile, as evidenced by the significant variations in the K_D and cooperativity (α) parameters with respect to the scaling factor.

2.6 *In Silico Modelling of Metal Templated Dimer:*

The nitroxide-nitroxide PELDOR distance was modelled *in silico* as described in section 1.8. A comparison of the experimental distributions (for the I6R1/K28H/Q32H construct in presence of either Tris-HCl or phosphate buffer) with the modelled distributions from MMM2018[11] and MTSSLWizard[10] are shown in figure S69. Despite minor differences, the simulated distributions agree reasonably with both experimental distributions, and confirms the presence of a Cu^{II}-templated dimer structure in solution.

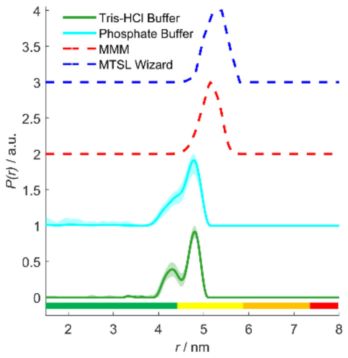

Figure S69. Experimental distance distributions for Tris-HCl and phosphate buffer (green and cyan traces, respectively) stacked with MMM (red) and MTSSLWizard (blue) simulations of the nitroxide-nitroxide distance distribution. The experimental data with the highest modulation-to-noise ratios were chosen from each series.

However, the experimental distances (~4.8 nm in both buffers are slightly shorter than the distances predicted by *in silico* modelling (~5.2-5.4 nm). This difference of 0.4-0.6 nm in the modal distance may be due to a small deviation from the square planar geometry of the metal coordination.[12] Additionally, the experimental distance distributions are bimodal, with shoulders observed at

~4.3 nm, and as alluded to in section 2.3.1, this may arise from differences in the coordination geometry of the metal centre, or indeed heterogeneous populations of parallel and antiparallel arrangements of monomers within the templated dimers.

2.7 Author contributions

Maria Oranges: Conceptualization (equal), investigation-sample preparation (equal), investigation-pulse EPR (lead), data curation (equal), formal analysis (equal), methodology (supporting), writing-original draft (lead), writing-review & editing (equal)

Joshua L. Wort: Conceptualization (equal), investigation-pulse EPR (supporting), data curation (equal), formal analysis (equal), methodology (lead), software (lead), supervision (supporting), writing-original draft (supporting), writing-review & editing (equal)

Miki Fukushima: Investigation-sample preparation (supporting), investigation-CW EPR (lead), data curation (supporting)

Edoardo Fusco: Investigation-pulse EPR (supporting), data curation (supporting)

Katrin Ackermann: Conceptualization (equal), investigation-sample preparation (equal), investigation-CW EPR (supporting), formal analysis (supporting), supervision (supporting), writing-review & editing (supporting)

Bela E. Bode: Conceptualization (equal), investigation-pulse EPR (supporting), formal analysis (equal), funding acquisition (lead), methodology (supporting), supervision (lead), writing-original draft (supporting), writing-review & editing (equal)

III **References:**

(1) Wort, J. L.; Ackermann, K.; Giannoulis, A.; Stewart, A. J.; Norman, D. G.; Bode, B. E. Sub-Micromolar Pulse Dipolar EPR Spectroscopy Reveals Increasing Cu^{II}-Labelling of Double-Histidine Motifs with Lower Temperature. *Angew. Chemie - Int. Ed.* **2019**, *58*, 11681–11685. https://doi.org/10.1002/anie.201904848.

(2) Lawless, M. J.; Ghosh, S.; Cunningham, T. F.; Shimshi, A.; Saxena, S. On the Use of the Cu^{2+}-Iminodiacetic Acid Complex for Double Histidine Based Distance Measurements by Pulsed ESR. *Phys. Chem. Chem. Phys.* **2017**, *19* (31), 20959–20967. https://doi.org/10.1039/c7cp02564e.

(3) Pannier, M.; Veit, S.; Godt, A.; Jeschke, G.; Spiess, H. W. Dead-Time Free Measurement of Dipole-Dipole Interactions between Electron Spins. *J. Magn. Reson.* **2000**, *142*, 331–340. https://doi.org/10.1006/jmre.1999.1944.

(4) Milikisyants, S.; Scarpelli, F.; Finiguerra, M. G.; Ubbink, M.; Huber, M. A Pulsed EPR Method to Determine Distances between Paramagnetic Centers with Strong Spectral Anisotropy and Radicals: The Dead-Time Free RIDME Sequence. *J. Magn. Reson.* **2009**, *201* (1), 48–56. https://doi.org/10.1016/j.jmr.2009.08.008.

(5) Ritsch, I.; Hintz, H.; Jeschke, G.; Godt, A.; Yulikov, M. Improving the Accuracy of Cu(II)-Nitroxide RIDME in the Presence of Orientation Correlation in Water-Soluble Cu(II)-Nitroxide Rulers. *Phys. Chem. Chem. Phys.* **2019**, *21* (19), 9810–9830. https://doi.org/10.1039/c8cp06573j.

(6) Abdullin, D.; Suchatzki, M.; Schiemann, O. Six-Pulse RIDME Sequence to Avoid Background Artifacts. *Appl. Magn. Reson.* **2021**. https://doi.org/10.1007/s00723-021-01326-1.

(7) Wort, J. L.; Ackermann, K.; Norman, D. G.; Bode, B. E. A General Model to Optimise CuIIlabelling Efficiency of Double-Histidine Motifs for Pulse Dipolar EPR Applications. *Phys. Chem. Chem. Phys.* **2021**, *23* (6), 3810–3819. https://doi.org/10.1039/d0cp06196d.

(8) Mack, E. T.; Perez-Castillejos, R.; Suo, Z.; Whitesides, G. M. Exact Analysis of Ligand-Induced Dimerization of Monomeric Receptors. *Anal. Chem.* **2008**, *80* (14), 5550–5555. https://doi.org/10.1021/ac800578w.

(9) Cunningham, T. F.; Putterman, M. R.; Desai, A.; Horne, W. S.; Saxena, S. The Double-Histidine Cu^{2+}-Binding Motif: A Highly Rigid, Site-Specific Spin Probe for Electron Spin Resonance Distance Measurements. *Angew. Chemie - Int. Ed.* **2015**, *54* (21), 6330–6334.

https://doi.org/10.1002/anie.201501968.
(10) Hagelueken, G.; Ward, R.; Naismith, J. H.; Schiemann, O. MtsslWizard: In Silico Spin-Labeling and Generation of Distance Distributions in PyMOL. *Appl. Magn. Reson.* **2012**, *42* (3), 377–391. https://doi.org/10.1007/s00723-012-0314-0.
(11) Ghosh, S.; Saxena, S.; Jeschke, G. Rotamer Modelling of Cu(II) Spin Labels Based on the Double-Histidine Motif. *Appl. Magn. Reson.* **2018**, *49* (11), 1281–1298. https://doi.org/10.1007/s00723-018-1052-8.
(12) Salgado, E. N.; Lewis, R. A.; Mossin, S.; Rheingold, A. L.; Tezcan, F. A. Control of Protein Oligomerization Symmetry by Metal Coordination: C 2 and C 3 Symmetrical Assemblies through Cu^{II} and Ni^{II} Coordination. *Inorg. Chem.* **2009**, *48*, 2726–2728. https://doi.org/10.1021/ic9001237.
(13) Ferreira, C. M. H.; Pinto, I. S. S.; Soares, E. V.; Soares, H. M. V. M. (Un)Suitability of the Use of PH Buffers in Biological, Biochemical and Environmental Studies and Their Interaction with Metal Ions – a Review. *RSC Adv.* **2015**, *5* (39), 30989–31003. https://doi.org/10.1039/C4RA15453C.
(14) Sokołowska, M.; Pawlas, K.; Bal, W. Effect of Common Buffers and Heterocyclic Ligands on the Binding of Cu(II) at the Multimetal Binding Site in Human Serum Albumin. *Bioinorg. Chem. Appl.* **2010**, *2010*, 1–7. https://doi.org/10.1155/2010/725153.
(15) Xiao, C.-Q.; Huang, Q.; Zhang, Y.; Zhang, H.-Q.; Lai, L. Binding Thermodynamics of Divalent Metal Ions to Several Biological Buffers. *Thermochim. Acta* **2020**, *691*, 178721. https://doi.org/10.1016/j.tca.2020.178721.
