# Supplementary material for: Pulse Dipolar Electron Paramagnetic Resonance Spectroscopy Reveals Buffer-Modulated Cooperativity of Metal-Templated Protein Dimerization
Source: J Phys Chem Lett. 2022 Aug 17;13(33):7847–52. doi: 10.1021/acs.jpclett.2c01719 (PMC9421889; doi:10.1021/acs.jpclett.2c01719)
Supplement: Supplementary file 2 — jz2c01719_si_002.pdf [file jz2c01719_si_002.pdf]

jz-2022-01719p.R1

Name: Peer Review Information for "Pulse Dipolar Electron Paramagnetic Resonance Spectroscopy Reveals Buffer Modulated Cooperativity of Metal Templated Protein Dimerization."

First Round of Reviewer Comments

Reviewer: 1

Comments to the Author

In this manuscript; the authors showed that they can follow self-assembly of proteins which occurred owing to the binding of paramagnetic copper ions. They used the well-studied GB1 protein, which was labeled in one place with nitroxide spin-label. The addition of Cu(II) to the solution affect the dimerization process. Using titration experiments and EPR measurements they evaluated the Kd values.

There are several novel things in this work:

the first thing is that it was conducted in an intelligent way. DEER experiments were used to follow nitroxide-nitroxide distance in the dimer, while RIDME follow Cu-NO distance. This allows to accurately evaluate the degree of assembly, and the Kd.

Second, the experiments were performed at two different buffers, which showed an effect of the buffer on the assembly process, and Cu(II) binding.

Overall this is a well performed work, and the novelty is high. therefore, I recommend publication.

Reviewer: 2

Comments to the Author

In this work the authors examine the metal templated dimerization using pulsed dipolar ESR spectroscopy. The authors systematically measure the modulation depths in DEER and RIDME as a function of Cu(II) concentration. These modulation depths are analyzed to infer details about binding, such as affinity of dimerization and cooperativity. Metal-templated biopolymerization is an attractive methodology to create functional polymers that for example exist in regular 2D or 3D arrays, and/or to place functional groups at regular positions. The method leverages the defined structure of proteins with coordination chemistry to create oligomers. The use of pulsed dipolar spectroscopy in this arena is both emerging as well as interesting given the difficulties of getting structural/mechanistic details on such systems. The work also logically builds on developments from the Bode group that relate modulation depths to thermodynamics parameters. Beyond the measurement of affinity parameters the authors potentially show that buffer can be used to modulate assembly. For all these reasons this is a

very interesting and novel paper and I am supportive of publication. On the other hand I have one major concern and also some .aspects of presentation that are important to address:

(a) One concern that the buffer (especially in the case of Tris) acts as a competitive binder to Cu(II) and the amount of Cu(II) available for binding to the protein is not constant but would vary with concentration. In particular I refer the authors to the following paper (https://doi.org/10.1042/bj2210559) The binding model seems to ignore this reality, which may well impact both the measured dissociation constants as as inference about cooperativity.

(b) The SI provide details of the kinetic model (Eq S5-S26). However, there is no clear equation that relates the modulation depth to the kinetic parameters. These should be outlined. In addition, I would add a few lines in the main explaining how the data in Figure 2a and 2b is used to obtain the thermodynamic parameters. Although the authors done much in this arena, such conceptual details are needed to enhance readability.

(c) Although this is pedantic, for reasons outlined above I would resist the use of Dissociation Constant as the descriptor for Kd. The authors are measuring apparent dissociation constants.

(d) In the main text, the authors should describe how the choice of the two buffers was made.

(e) There is some precedence of the use of PDS ESR and even Cu(II) based templated measurements to understand biopolymerization. (Chem. Comm. 55 (2019) 7752; J. Phys. Chem. B 118 (2014) 9881)

Reviewer: 3

Comments to the Author

Using DEER and RIDME complementarily to study the extract cooperativity and KD parameters of a metal-templated dimerized-protein system is quite interesting. Indeed, this work showcase the robustness and accuracy of PDS in monitoring equilibria processes of cooperativity model.

1. Effect of buffers and heterocyclic ligands on the binding of Cu(II) should be checked carefully in all titration/pseudo-titration experiments. As the authors mentioned, quite a lot articles have discussed about it in details. The authors also showed that the solution-retained Cu(II) varied significantly with different additive factors (line 43-54, page 7). It is suggesting that the Cu(II) in solution is under dynamic equilibrium while the total soluble Cu(II) is reducing continuously with precipitation. The corrected soluble Cu(II) may lose its effectiveness.

2. The conclusion “in presence of phosphate buffer, Cu(II)-templated dimerization demonstrated apparent positive cooperativity, while in Tris-HCl buffer, this templated dimerization displayed strongly negative cooperativity behaviour” was based on the binding affinity calculations in figure 3 and 4. Indeed, “Positive cooperativity in metal-templated dimerization is considered to be rare”. The quantification of Cu(II) concentration for all the important samples should be taken into consideration before further calculation. RMSD method is not sufficient to rule out all the other possibilities, i.e. the soluble Cu(II) concentration in the 150[illegible]M sample (figure 2) under PDS-measuring condition is overestimated.

3. Also the authors mentioned, “metal-induced stabilization of the α-helix motif” has been already studied in several works, especially for GB1 model system measured by PDS. The intention of using I6H/N8H/K28R1 construct in this draft is not clear, since it is not relevant to the main part of this work, nor the [illegible]-sheet motif has not been discussed neither.

Author's Response to Peer Review Comments:

Please see attached file

Dr Bela Bode
Biomolecular Sciences Research Complex
Centre of Magnetic Resonance

Manuscript: "Pulse dipolar EPR spectroscopy reveals buffer modulated cooperativity of metal templated protein dimerization."

St Andrews, 20th July 2022

Dear Professor [redacted],

We are submitting the revised manuscript ***"Pulse dipolar EPR spectroscopy reveals buffer modulated cooperativity of metal templated protein dimerization"*** authored by Dr Maria Oranges, Dr Joshua L. Wort, Ms Miki Fukushima, Mr Edoardo Fusco, Dr Katrin Ackermann and Dr Bela E. Bode for your kind consideration for publication as a Letter in *The Journal of Physical Chemistry Letters*.

We have carefully considered and responded to all reviewer comments and believe to have addressed them in full. Please find comments and responses overleaf. We hope the manuscript is now acceptable for publication.

This manuscript has just been posted as a pre-print on chemRxiv: https://doi.org/10.26434/chemrxiv-2022-ht2gb

This research was funded, in whole or in part, by the Wellcome Trust. A CC BY or equivalent license is applied to the Author Accepted Manuscript arising from this submission, in accordance with the grant's open access conditions.

Sincerely yours,

[redacted]

Responses to reviewer comments.

Comments by editors and reviewers are given in black and responses in blue.

Editor

1. Title: In both the main manuscript file and the Supporting Information, set the title in title case, with the first letter of each principal word capitalized.

This was corrected.

2. TOC Graphic: Provide a TOC image per journal guidelines (2 in x 2 in; on the same page as the abstract) with the heading “TOC Graphic” above the graphic. The graphic should be in the form of a structure, graph, drawing, photograph, or scheme—or a combination. Non-scientific cartoon-like images or caricatures are discouraged. https://pubsapp.acs.org/paragonplus/submission/toc_abstract_graphics_guidelines.pdf

This has now been included.

3. Title: Title must match (including punctuation) in three places: (1) manuscript file, (2) supporting information, and (3) ACS Paragon Plus.

This has now been corrected.

4. Title: Using acronyms in title is discouraged. Please spell out all acronyms in the title of the manuscript and Supporting Information.

The abbreviation EPR has been replaced.

5. References: In both the main file and the supporting information, fix the style of all references to use JPCL formatting (check all references carefully). ***JPC Letters reference formatting requires that journal references should contain: () around numbers, author names, article title (titles entirely in title case or entirely in lower case), abbreviated journal title (italicized), year (bolded), volume (italicized), and pages (first-last). Book references should contain author names, book title (in the same pattern), publisher, city, and year

This has been changed and double-checked.

Reviewer: 1

Recommendation: This paper represents a significant new contribution and should be published as is.

Comments:

In this manuscript; the authors showed that they can follow self-assembly of proteins which occurred owing to the binding of paramagnetic copper ions. They used the well-studied GB1 protein, which was labeled in one place with nitroxide spin-label. The addition of Cu(II) to the solution affect the dimerization process. Using titration experiments and EPR measurements they evaluated the Kd values.

There are several novel things in this work:

the first thing is that it was conducted in an intelligent way. DEER experiments were used to follow nitroxide-nitroxide distance in the dimer, while RIDME follow Cu-NO distance. This allows to accurately evaluate the degree of assembly, and the Kd.

Second, the experiments were performed at two different buffers, which showed an effect of the buffer on the assembly process, and Cu(II) binding.

Overall this is a well performed work, and the novelty is high. therefore, I recommend publication.

We thank the reviewer for their appreciation of our work and experiment design.

Additional Questions:

Urgency: High

Significance: Top 10%

Novelty: Top 10%

Scholarly Presentation: Top 10%

Is the paper likely to interest a substantial number of physical chemists, not just specialists working in the authors' area of research?: Yes

Reviewer: 2

Recommendation: This paper may be publishable, but major revision is needed; I would like to be invited to review any future revision.

Comments:

In this work the authors examine the metal templated dimerization using pulsed dipolar ESR spectroscopy. The authors systematically measure the modulation depths in DEER and RIDME as a function of Cu(II) concentration. These modulation depths are analyzed to infer details about binding, such as affinity of dimerization and cooperativity. Metal-templated biopolymerization is an attractive methodology to create functional polymers that for example exist in regular 2D or 3D arrays, and/or to place functional groups at regular positions. The method leverages the defined structure of proteins with coordination chemistry to create oligomers. The use of pulsed dipolar spectroscopy in this arena is both emerging as well as interesting given the difficulties of getting structural/mechanistic details on such systems. The work also logically builds on developments from the Bode group that relate modulation depths to thermodynamics parameters. Beyond the measurement of affinity parameters the authors potentially show that buffer can be used to modulate assembly. For all these reasons this is a very interesting and novel paper and I am supportive of publication. On the other hand I have one major concern and also some .aspects of presentation that are important to address:

We thank the reviewer for their support and appreciation of the relevance of our work and address individual comments below.

(a) One concern that the buffer (especially in the case of Tris) acts as a competitive binder to Cu(II) and the amount of Cu(II) available for binding to the protein is not constant but would vary with concentration. In particular I refer the authors to the following paper

(https://doi.org/10.1042/bj2210559) The binding model seems to ignore this reality, which may well impact both the measured dissociation constants as as inference about cooperativity.

The reviewer is entirely correct in the fact that the availability of copper(II) is strongly buffer dependent and unfortunately there are no known buffers that do not compete with aqueous copper(II) in some way. However, we need to use a buffered system for protein stability. Even the protein itself is known to have less specific binding sites and the problem of knowing the free copper(II) concentration in solution is therefore not straightforward. All our dissociation constants and cooperativities are "apparent" and we have adapted the text to be clearer in this.

(b) The SI provide details of the kinetic model (Eq S5-S26). However, there is no clear equation that relates the modulation depth to the kinetic parameters. These should be outlined. In addition, I would add a few lines in the main explaining how the data in Figure 2a and 2b is used to obtain the thermodynamic parameters. Although the authors done much in this arena, such conceptual details are needed to enhance readability.

We thank the reviewer for highlighting this. We attempted to avoid unnecessary repetition from previous work but have adapted this section to reflect that the modulation depths report on the fraction of nitroxide dipolarly coupled to either copper(II) (RIDME) or another nitroxide (DEER). We have included a definition of Δ in Figure 2 and updated the section to make the workflow more easily traceable.

(c) Although this is pedantic, for reasons outlined above I would resist the use of Dissociation Constant as the descriptor for Kd. The authors are measuring apparent dissociation constants.

We fully agree, as stated in response to the 1st comment.

(d) In the main text, the authors should describe how the choice of the two buffers was made.

We agree that the selection criteria (rather than the full set of experiments underpinning selection) and choice should be described in the main text and have added this.

(e) There is some precedence of the use of PDS ESR and even Cu(II) based templated measurements to understand biopolymerization. (Chem. Comm. 55 (2019) 7752; J. Phys. Chem. B 118 (2014) 9881)

Thank you for highlighting this, we have included these references.

Additional Questions:

Urgency: High

Significance: High

Novelty: Top 10%

Scholarly Presentation: Moderate

Is the paper likely to interest a substantial number of physical chemists, not just specialists working in the authors' area of research?: Yes

Reviewer: 3

Recommendation: This paper is probably publishable, but major revision is needed; I do not need to see future revisions.

Comments:

Using DEER and RIDME complementarily to study the extract cooperativity and KD parameters of a metal-templated dimerized-protein system is quite interesting. Indeed, this work showcase the robustness and accuracy of PDS in monitoring equilibria processes of cooperativity model.

We thank the reviewer for their appreciation for our experiments as a showcase for the robustness of PDS and being an interesting showcase of metal-templated dimerization.

1. Effect of buffers and heterocyclic ligands on the binding of Cu(II) should be checked carefully in all titration/pseudo-titration experiments. As the authors mentioned, quite a lot articles have discussed about it in details. The authors also showed that the solution-retained Cu(II) varied significantly with different additive factors (line 43-54, page 7). It is suggesting that the Cu(II) in solution is under dynamic equilibrium while the total soluble Cu(II) is reducing continuously with precipitation. The corrected soluble Cu(II) may lose its effectiveness.

We fully agree with reviewer 3 (as also indicated for reviewer 2) that the free copper(II) concentration will be strongly influenced by all buffers and binding to low affinity protein sites. We do not think that a true concentration can be established from our data but that the apparent K_D and cooperativity are important system descriptors that we can reliably extract. We adapted the text to emphasise this.

2. The conclusion “in presence of phosphate buffer, Cu(II)-templated dimerization demonstrated apparent positive cooperativity, while in Tris-HCl buffer, this templated dimerization displayed strongly negative cooperativity behaviour” was based on the binding affinity calculations in figure 3 and 4. Indeed, “Positive cooperativity in metal-templated dimerization is considered to be rare”. The quantification of Cu(II) concentration for all the important samples should be taken into consideration before further calculation. RMSD method is not sufficient to rule out all the other possibilities, i.e. the soluble Cu(II) concentration in the 150�M sample (figure 2) under PDS-measuring condition is overestimated.

Unfortunately, the reviewer makes no suggestion of how to reliably quantify the true free copper(II) concentration. In *lieu* of a reliable quantification, this approach is futile. The RMSD method is at least internally consistent and indicates that the free copper(II) seems to be severely reduced in phosphate buffer. We tried to make this even clearer and more transparent.

3. Also the authors mentioned, “metal-induced stabilization of the α-helix motif” has been already studied in several works, especially for GB1 model system measured by PDS. The intention of using I6H/N8H/K28R1 construct in this draft is not clear, since it is not relevant to the main part of this work, nor the �-sheet motif has not been discussed neither.

We did not see significant dimer formation in the construct the reviewer mentions. We think this is an important result that should be shared with the community and is not related to stabilisation of secondary structure elements. We have rephrased this section to make this point more clearly.

Additional Questions:

Urgency: Moderate

Significance: Moderate

Novelty: Moderate

Scholarly Presentation: High

Is the paper likely to interest a substantial number of physical chemists, not just specialists working in the authors' area of research?: Yes

jz-2022-01719p.R2

Name: Peer Review Information for "Pulse Dipolar Electron Paramagnetic Resonance Spectroscopy Reveals Buffer Modulated Cooperativity of Metal Templated Protein Dimerization."

Second Round of Reviewer Comments

Reviewer: 2

Comments to the Author

I am satisfied by the response from the authors. I recommend publication.

Reviewer: 1

Comments to the Author

I have no comments

Reviewer: 3

Comments to the Author

The revised mauscript was significantly improved and my concerns have been carefully addressed.

In page 11 and SI section (2.4), the authors studied the EPR spectra of CuCl2 in different buffers at pH 7.4. Indeed, the copper salt behaves differently in the different buffers above neutral pH due to the hydrolysis of Cu(II). I therefore recommend the authors to take caution in the making the respective statement. In addition, I recommond the authors to use buffer like MES around pH 6.5 to avoid the hydrolysis of copper for better comprison, since metal driven self-assembly is a common phenonmenon and GB1 itself has several copper binding sites. The obervation by the authors are true but the impact of metal binding sites for copper has to be commented since copper is very special transition metal in addition to the buffer effect. Overall, this is a solid work and I recommend for publication in JPC Letters.

Author's Response to Peer Review Comments:

see attached file

Dr Bela Bode
Biomolecular Sciences Research Complex
Centre of Magnetic Resonance

Manuscript: "Pulse Dipolar Electron Paramagnetic Resonance Spectroscopy Reveals Buffer Modulated Cooperativity of Metal Templated Protein Dimerization."

St Andrews, 5th August 2022

Dear Professor [redacted]

We are submitting the revised manuscript ***"Pulse Dipolar Electron Paramagnetic Resonance Spectroscopy Reveals Buffer Modulated Cooperativity of Metal Templated Protein Dimerization"*** authored by Dr Maria Oranges, Dr Joshua L. Wort, Ms Miki Fukushima, Mr Edoardo Fusco, Dr Katrin Ackermann and Dr Bela E. Bode for your kind consideration for publication as a Letter in *The Journal of Physical Chemistry Letters*.

We have carefully considered reviewer 3's further comments. Please find comments and responses overleaf. We hope the manuscript is now acceptable for publication.

This manuscript has just been posted as a pre-print on chemRxiv: https://doi.org/10.26434/chemrxiv-2022-ht2gb

This research was funded, in whole or in part, by the Wellcome Trust. A CC BY or equivalent license is applied to the Author Accepted Manuscript arising from this submission, in accordance with the grant's open access conditions.

Sincerely yours,

[redacted]

Reviewer: 2
"I am satisfied by the response from the authors. I recommend publication."

Reviewer: 1
"I have no comments."

Response: both reviewers recommend publication as is and we are grateful for their positive assessment.

Reviewer: 3
"The revised mauscript was significantly improved and my concerns have been carefully addressed."

Response: We are happy to read the appraisal by reviewer 3.

"In page 11 and SI section (2.4), the authors studied the EPR spectra of CuCl2 in different buffers at pH 7.4. Indeed, the copper salt behaves differently in the different buffers above neutral pH due to the hydrolysis of Cu(II). I therefore recommend the authors to take caution in the making the respective statement. In addition, I recommond the authors to use buffer like MES around pH 6.5 to avoid the hydrolysis of copper for better comprison, since metal driven self-assembly is a common phenonmenon and GB1 itself has several copper binding sites. The obervation by the authors are true but the impact of metal binding sites for copper has to be commented since copper is very special transition metal in addition to the buffer effect. Overall, this is a solid work and I recommend for publication in JPC Letters."

Even after reading this comment multiple times, we are not entirely certain what the reviewer aims to convey. The "hydrolysis of copper(II)" (we suppose some unspecified complexes of copper?) and "respective statement" remain too vague for us to identify the specific issue the reviewer wants to raise. Their recommendation to use a morpholine-based buffer that is effective at mildly acidic pH seems to aim at using a weakly complexing buffering agent at a pH range that allows a larger solubility of aqueous copper(II) ions. While this may be an interesting regime to test if fine tuning the apparent cooperativity, it would not expand the scope of our proof-of-principle that the buffer conditions strongly influence observed cooperativity. Considering that each single sample (titration point) takes about 24 h of pulse EPR (16 h for DEER and 6 h for RIDME plus set up time) and a day instrument time is normally charged at £800, we do not see how the mere confirmation of buffer induced differences would justify the extra cost.

Thus, we briefly discuss the potential of using a weakly complexing buffer at mildly acidic pH and the competition of low affinity binding sites on the protein surface (this was in the supplement but not explicitly mentioned in the main text).
"It is important to note that additional low-affinity Cu^{II}-binding sites on the protein surface, precipitation in alkaline pH, and complexation by buffer components are all potentially limiting the available Cu^{II} for ligation by one or two dHis motifs. In this context, the study of e.g., morpholine-based buffers effective in mildly acidic conditions (such as MES) may be interesting to maximise Cu^{II} availability."
